# Trends of Non‐Hodgkin Lymphoma Incidence Among Adults in the United States From 2000 to 2020

**DOI:** 10.1002/cnr2.70269

**Published:** 2025-06-30

**Authors:** Armin Aslani, Morvarid Najafi, Seyed Ehsan Mousavi, Hanieh Marandi, Zahra Yekta, Seyed Aria Nejadghaderi

**Affiliations:** ^1^ Social Determinants of Health Research Center, Department of Community Medicine Faculty of Medicine, Tabriz University of Medical Sciences Tabriz Iran; ^2^ Center for Orthopedic Trans‐Disciplinary Applied Research Tehran University of Medical Sciences Tehran Iran; ^3^ Neurosciences Research Center, Aging Research Institute Tabriz University of Medical Sciences Tabriz Iran; ^4^ School of Medicine Urmia University of Medical Sciences Urmia Iran; ^5^ Calaveras County Department of Health Calaveras County California USA; ^6^ HIV/STI Surveillance Research Center, and WHO Collaborating Center for HIV Surveillance, Institute for Futures Studies in Health Kerman University of Medical Sciences Kerman Iran; ^7^ Knowledge Hub for Migrant and Refugee Health, Institute for Futures Studies in Health Kerman University of Medical Sciences Kerman Iran

**Keywords:** hematological neoplasms, incidence, non‐Hodgkin Lymphoma, SEER

## Abstract

**Background:**

Non‐Hodgkin lymphoma (NHL) comprises a broad range of hematologic cancers originating from lymphoid tissues. It ranks among the 10 most frequently diagnosed cancers in the United States (US).

**Aims:**

This study aims to analyze trends in the incidence of adult NHL in the US from 2000 to 2020, considering factors such as age, sex, race/ethnicity, and histological subtypes. Additionally, the impact of the COVID‐19 pandemic on these incidence trends was explored.

**Methods and Results:**

Data from the Surveillance, Epidemiology, and End Results program were utilized to examine the age‐standardized incidence rates (ASIR) of NHL. Joinpoint regression modeling was applied to calculate the annual percent change (APC) and the average annual percent change (AAPC) of ASIRs over the period from 2000 to 2020. From 2000 to 2019, a total of 962 535 NHL were reported among all ages in the US. They were mostly B‐cell NHL (93.59%), in Non‐Hispanic Whites (73.31%), and individuals aged 70–79 (25.94%). The overall ASIRs were 55.58 (55.43, 55.73) for men and 36.00 (35.89, 36.11) for women. There was a notable decline in ASIRs following the onset of the COVID‐19 pandemic from 2019 to November 2020, with a percentage change of −10.52% (−11.60, −9.45). The overall AAPC for adult NHL was 0.45% (0.33, 0.62) in men and 0.38% (0.21, 0.57) in women, indicating a minimal yet significant increase.

**Conclusion:**

NHL incidence increased over 2000–2019. These trends in incidence rates exhibited variation across different races, sexes, age groups, and histological subtypes. COVID‐19 led to a decrease in NHL incidence.

## 1 Introduction

Non‐Hodgkin lymphoma (NHL) encompasses a wide variety of hematologic cancers, consisting of over 70 distinct subtypes that originate from lymphoid tissue cells [1]. For each subtype of NHL, a distinct cell of origin may be identified according to immunophenotypic, genetic, and clinical features [1]. Diffuse large B‐cell lymphoma (DLBCL) is the most prevalent subtype of NHL in Western countries and is characterized by its aggressive nature. In contrast, follicular lymphoma, the second most common subtype, tends to follow a more indolent course [1]. The pathogenesis of NHL is a result of the accumulation of genetic lesions resulting from chromosomal translocations, somatic mutations, genome damage, and infection with oncogenic viruses [2, 3].

NHL can develop at any age and is notably among the more prevalent cancers in children, teenagers, and young adults [4]. However, the likelihood of developing NHL increases with age, with 57% of cases diagnosed in individuals over the age of 65 [5]. A male predominance is seen in the incidence rates of almost all subtypes of NHL [6], which may be attributed to differences in environmental exposures and intrinsic disparities in immunological and hormonal factors between men and women [7, 8]. Whites are also more commonly affected than African Americans [9]. Additionally, NHL tends to cluster in families and certain geographical locations, requiring investigations into genetic susceptibility, lifestyle factors, and environmental exposures [10]. Currently, NHL represents 4.1% of all new cancer cases in the United States (US), with over 80 000 new cases projected for 2024 [10]. Several prior studies have documented the incidence of NHL across different age groups, sexes, racial/ethnic backgrounds, and histological subtypes in the US [4, 9, 11, 12]. However, these studies relied on earlier versions of the SEER database and did not assess the impact of the COVID‐19 pandemic. Our study specifically examines the incidence rates of NHL using the latest SEER data, providing updated insights across various demographic categories and histological subtypes. We also included data from the COVID‐19 pandemic and its effect on NHL.

## 2 Methods

### 2.1 Data Source

The SEER program was used in this study [13]. In this study, we estimated the incidence rates and annual percent changes (APCs) of NHL using the SEER 22 database [14]. The access to this database was established under the terms of the SEER research data use agreement for the 1975–2020 Data [15]. In this study, we specifically utilized data from the SEER program covering the years 2000–2020.

### 2.2 Definitions

The APC, which indicates trends in cancer rates over time, is determined by assuming that cancer rates change by a constant percentage relative to the rate of the previous year. To summarize the average APCs over multiple years, the average annual percent change (AAPC) is calculated as a weighted average of the APCs derived from the joinpoint model, with the weights corresponding to the length of each APC interval. This study analyzed three primary categories based on race/ethnicity: Non‐Hispanic White (NHW), Non‐Hispanic Black (NHB), and Hispanic. Due to the limited number of cases, data from American Indian/Alaska Native, Native Hawaiian, and Asian/Pacific Islander populations were only included in the overall estimates.

We used the Lymphoid Neoplasms Recode 2021 revision for classification [16], which is the preferred classification system according to SEER 22 [13]: “2(a). Non‐Hodgkin lymphoma, B‐cell; 2(a)1. Precursor Non‐Hodgkin lymphoma, B‐cell; 2(a)2. Mature Non‐Hodgkin lymphoma, B‐cell; 2(a)3. Non‐Hodgkin lymphoma, B‐cell, NOS; 2(b). Non‐Hodgkin lymphoma, T‐cell; 2(b)1. Precursor Non‐Hodgkin lymphoma, T‐cell; 2(b)2. Mature Non‐Hodgkin lymphoma, T‐cell; and 2(c). Non‐Hodgkin lymphoma, unknown lineage”.

### 2.3 Statistical Analysis

We report overall trends using the age‐standardized incidence rate (ASIR), which reflects incidence adjusted for age across the population. For age‐specific trends, we report incidence rates for defined age groups and refer to these as ‘incidence rates’ to maintain clarity and conciseness [14, 17]. The cases included in the study are those with a diagnosis of malignant cancer with known age. The estimations of APCs, AAPCs [18], Jointpoint regression modeling, parallelism test and coincidence tests for ASIR [19, 20] were run by the Jointpoint Regression Program, version 5.0.2 [19]. To avoid bias in the cancer incidence estimates, data from the year 2020 was excluded from the joinpoint trend analysis and was only presented in illustrations [21]. The ASIRs of NHL and their APCs were calculated by fitting least‐squares regression lines to the natural logarithm of the ASIR, using diagnosis year as the regressor variable. Model selection was performed using the weighted Bayesian Information Criteria technique [17]. The 95% confidence intervals (CIs) were determined using the empirical quartile method [22].

## 3 Results

### 3.1 Non‐Hodgkin Lymphoma

**FIGURE 1:**
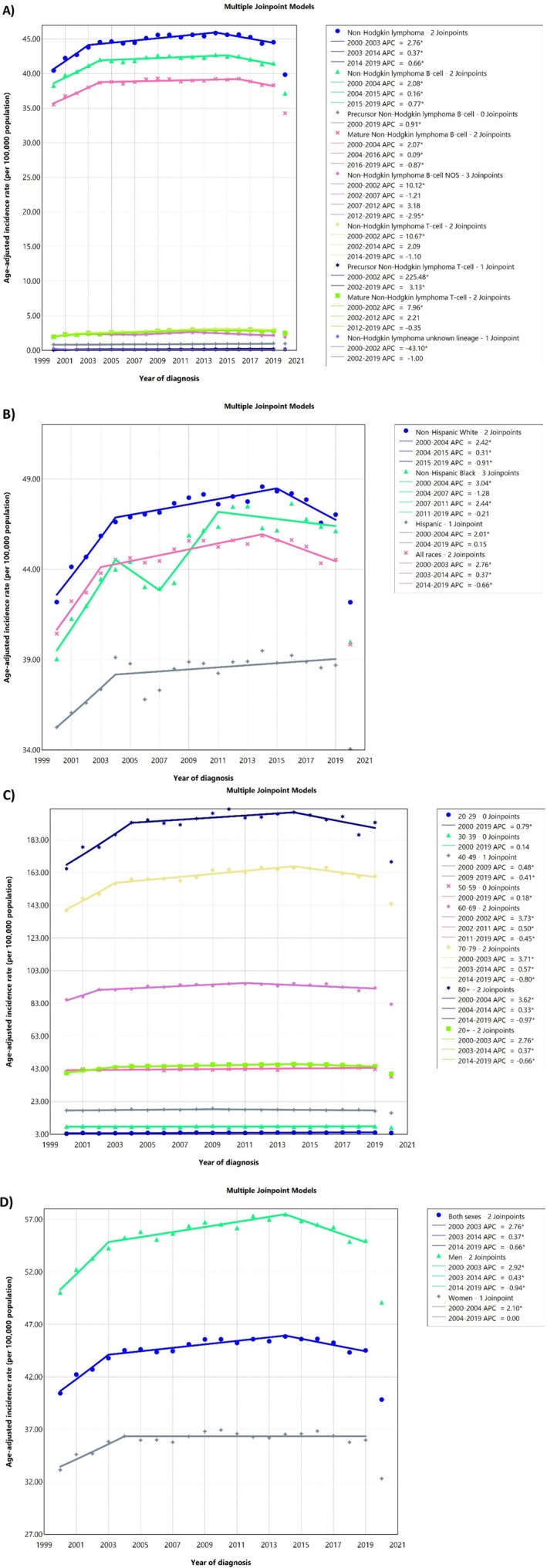
Delayed age‐adjusted incidence rate of adult non‐Hodgkin lymphoma over 2000–2019 and in 2020 in the United States, by cancer subtype (A), race/ethnicity (B), age (C), and sex (D). APC, annual percent change. * Represent *p* value less than 0.05.

From 2000 to 2019, a total of 962 535 cases of NHL were reported in the US across all age groups. B‐cell NHL was the most prevalent subtype, accounting for 93.59% of cases (Table 1). The majority of cases were identified among NHWs at 73.31%, with a significant proportion occurring in individuals aged 70–79 years (25.94%) (Table 1 and Figure 1). The ASIRs, per 100 000 population, were 55.58 (55.43, 55.73) for men and 36.00 (35.89, 36.11) for women. Both sexes had an increase in AAPCs (Men: 0.45% [0.33, 0.62]; Women: 0.38% [0.21, 0.57]) (Table 1 and Figure 1).

**TABLE 1:** Counts and age‐standardized rate of non‐Hodgkin lymphoma incidence per 100 000 and average annual percent change from 2000 to 2019 in the United States, by age, sex, and race.

Age group (years)	Men			Women		
	Case (%)	ASIR^a^ (95% CI)	AAPC (95% CI)	Case (%)	ASIR (95% CI)	AAPC (95% CI)
Race/ethnicities						
All	534 285 (55.51)	55.58 (55.43, 55.73)	0.45 (0.33, 0.62)	428 250 (44.49)	36 (35.89, 36.11)	0.38 (0.21, 0.57)
20–29	9839 (1.02)	4.61 (4.52, 4.7)	0.81 (0.48, 1.17)	6334 (0.66)	3.08 (3.01, 3.16)	0.73 (0.17, 1.34)
30–39	18 569 (1.93)	9.15 (9.02, 9.29)	−0.24 (−0.57, 0.1)	12 677 (1.32)	6.26 (6.15, 6.37)	0.68 (0.3, 1.08)
40–49	44 376 (4.61)	21.44 (21.24, 21.64)	−0.42 (−0.59, −0.25)	30 461 (3.16)	14.42 (14.26, 14.58)	0.63 (0.38, 0.87)
50–59	94 431 (9.81)	51.28 (50.95, 51.61)	0.11 (−0.06, 0.29)	67 721 (7.04)	34.96 (34.7, 35.23)	0.25 (0.04, 0.47)
60–69	136 810 (14.21)	112.99 (112.39, 113.59)	0.44 (0.24, 0.68)	101 800 (10.58)	75.18 (74.72, 75.64)	0.21 (0.07, 0.38)
70–79	137 258 (14.26)	199.59 (198.53, 200.65)	0.78 (0.65, 0.93)	112 421 (11.68)	128.73 (127.98, 129.49)	0.51 (0.29, 0.87)
≥ 80	93 002 (9.66)	259.58 (257.91, 261.25)	0.69 (0.52, 0.88)	96 836 (10.06)	154.49 (153.51, 155.47)	0.32 (0.07, 0.65)
Hispanic						
All	60 022 (53.85)	45.47 (45.06, 45.87)	0.18 (−0.11, 0.53)	51 439 (46.15)	32.82 (32.52, 33.11)	0.7 (0.26, 1.24)
20–29	2913 (2.61)	4.71 (4.54, 4.88)	1.72 (0.86, 2.67)	1810 (1.62)	3.25 (3.1, 3.4)	1.17 (0.19, 2.27)
30–39	4524 (4.06)	8.37 (8.12, 8.62)	−0.33 (−0.89, 0.29)	2990 (2.68)	5.88 (5.67, 6.09)	1.01 (0.25, 1.87)
40–49	8155 (7.32)	18.67 (18.26, 19.07)	−0.62 (−0.98, −0.25)	5728 (5.14)	13.34 (13, 13.69)	0.72 (0.08, 1.48)
50–59	12 160 (10.91)	41.78 (41.04, 42.53)	0.21 (−0.06, 0.53)	9918 (8.90)	32.64 (32, 33.29)	0.56 (0.24, 0.93)
60–69	13 736 (12.32)	90.19 (88.68, 91.72)	0.31 (−0.04, 0.76)	12 264 (11.00)	69.4 (68.17, 70.64)	0.41 (0.05, 0.84)
70–79	11 762 (10.55)	160.93 (158.02, 163.89)	0.23 (−0.25, 0.82)	11 529 (10.34)	117.41 (115.27, 119.58)	0.3 (−0.09, 0.76)
≥ 80	6772 (6.08)	209 (204.04, 214.06)	0.14 (−0.45, 0.92)	7200 (6.46)	132.94 (129.88, 136.05)	0.86 (−0.68, 2.73)
NHB						
All	49 202 (51.97)	55.36 (54.83, 55.88)	0.83 (0.68, 1.03)	45 479 (48.03)	37.88 (37.53, 38.24)	0.86 (0.49, 1.29)
20–29	1249 (1.32)	4.73 (4.47, 5)	−0.08 (−2.16, 2.38)	884 (0.93)	3.25 (3.04, 3.47)	−0.21 (−1.49, 1.13)
30–39	2645 (2.79)	11.54 (11.11, 11.99)	−0.07 (−1.06, 0.89)	2111 (2.23)	8.3 (7.95, 8.66)	0.37 (−0.55, 1.32)
40–49	6132 (6.48)	26.58 (25.92, 27.26)	−0.24 (−0.82, 0.34)	4967 (5.25)	19.13 (18.6, 19.67)	1.21 (0.61, 1.84)
50–59	11 331 (11.97)	59.29 (58.2, 60.39)	0.26 (−0.82, 1.26)	9071 (9.58)	40.71 (39.88, 41.56)	0.64 (0.16, 1.18)
60–69	13 183 (13.92)	118.66 (116.64, 120.72)	1.65 (0.87, 2.58)	11 627 (12.28)	81.37 (79.89, 82.87)	1.03 (0.52, 1.63)
70–79	9823 (10.37)	181.37 (177.77, 185.02)	1.3 (0.61, 2.24)	10 274 (10.85)	125.82 (123.39, 128.28)	0.89 (0.07, 2.32)
≥ 80	4839 (5.11)	212.65 (206.67, 218.75)	1.08 (0.63, 1.6)	6545 (6.91)	135.55 (132.28, 138.88)	0.42 (−0.15, 1.08)
NHW						
All	397 205 (56.28)	58.95 (58.77, 59.14)	0.51 (0.34, 0.7)	308 505 (43.72)	37.28 (37.15, 37.42)	0.43 (0.3, 0.57)
20–29	4813 (0.68)	4.57 (4.44, 4.7)	0.16 (−0.43, 0.77)	2969 (0.42)	2.9 (2.8, 3.01)	0.22 (−0.63, 1.1)
30–39	9915 (1.40)	9.33 (9.15, 9.52)	−0.48 (−0.87, −0.1)	6383 (0.90)	6.09 (5.94, 6.24)	0.39 (0.03, 0.75)
40–49	27 292 (3.87)	22.32 (22.05, 22.58)	−0.2 (−0.45, 0.06)	17 596 (2.49)	14.38 (14.17, 14.59)	0.6 (0.31, 0.89)
50–59	65 718 (9.31)	54.12 (53.71, 54.54)	0.06 (−0.19, 0.46)	44 563 (6.31)	35.78 (35.45, 36.12)	0.14 (−0.05, 0.32)
60–69	102 676 (14.55)	120.05 (119.32, 120.79)	0.3 (0.12, 0.5)	72 428 (10.26)	78.32 (77.75, 78.89)	0.25 (−0.03, 0.62)
70–79	109 232 (15.48)	213.56 (212.29, 214.83)	0.88 (0.72, 1.06)	85 173 (12.07)	135.11 (134.2, 136.02)	0.54 (0.38, 0.78)
≥ 80	77 559 (10.99)	277.35 (275.4, 279.31)	0.88 (0.7, 1.12)	79 393 (11.25)	162.79 (161.65, 163.93)	0.52 (0.26, 0.85)

Abbreviations: AAPC, average annual percent change; CI, confidence interval; NHB, non‐Hispanic Black; NHW, non‐Hispanic White.

^a^ The ASIR in the ‘All’ rows refers to the age‐standardized incidence rate. For individual age categories, the values represent age‐specific incidence rates.

Between 2015 and 2019, a total of 281 641 cases of NHL were reported. The majority of these cases were identified among NHWs, accounting for 69.67%, and in individuals aged 60–69 years, who represented 27.16% of cases (Table S1). NHW men had the highest ASIR (per 100 000 population) among all racial and ethnic groups, at 59.66 (95% CI: 59.30, 60.02) (Table S1). Importantly, there was a significant decline in ASIRs per 100 000 population during this period for both men (AAPC: −0.94% [95% CI: −1.61, −0.51]) and women (AAPC: −0.60% [95% CI: −1.36, −0.01]) (Table S1). Additional details on the similar and parallel trends of NHL can be found in Tables S2 and S3.

The number of incident cases rose with age, reaching its highest point in the 65–69 age group for men and the 70–74 age group for women. Both sexes experienced peak incidence rates in the 80–84 age category (Figure S1).

### 3.2 COVID‐19 Impacts

There was a notable decline in the ASIRs per 100 000 population across all races and both sexes, regardless of age, with an overall percentage change (PC) of −10.52% (95% CI: −11.60, −9.45) (Table 2). This decrease was also significant for males (PC: −10.72% [95% CI: −12.15, −9.28]) and females (PC: −10.19% [95% CI: −11.83, −8.56]) from 2019 to November 2020 (Table 2).

**TABLE 2:** Percent change in age‐standardized incidence rates of non‐Hodgkin lymphoma from 2019 to 2020 by race and sex.

Races/ethnicities	Sex	2019 ASIR (95% CI)	2020 ASIR (95% CI)	PC (95% CI)
All	Both	44.53 (44.16, 44.9)	39.84 (39.5, 40.19)	−10.52 (−11.6, −9.45)
	Female	35.98 (35.53, 36.43)	32.31 (31.89, 32.74)	−10.19 (−11.83, −8.56)
	Male	54.99 (54.38, 55.61)	49.1 (48.53, 49.67)	−10.72 (−12.15, −9.28)
Hispanic	Both	38.69 (37.81, 39.58)	34.06 (33.26, 34.87)	−11.97 (−14.85, −9.08)
	Female	33.01 (31.93, 34.12)	29.4 (28.4, 30.42)	−10.96 (−15.19, −6.73)
	Male	45.81 (44.37, 47.29)	40.01 (38.69, 41.36)	−12.67 (−16.68, −8.65)
NHB	Both	46.13 (44.95, 47.33)	39.99 (38.9, 41.1)	−13.31 (−16.57, −10.05)
	Female	39.07 (37.64, 40.53)	33.24 (31.94, 34.59)	−14.9 (−19.5, −10.29)
	Male	55.92 (53.89, 58)	49.34 (47.46, 51.28)	−11.76 (−16.45, −7.07)
NHW	Both	47.02 (46.55, 47.51)	42.18 (41.72, 42.63)	−10.31 (−11.64, −8.98)
	Female	37.35 (36.77, 37.94)	33.55 (32.99, 34.11)	−10.18 (−12.23, −8.12)
	Male	58.5 (57.72, 59.29)	52.48 (51.74, 53.22)	−10.3 (−12.05, −8.56)

Abbreviations: ASIR, age‐standardized incidence rate; CI, confidence interval; NHB, non‐Hispanic Black; NHW, non‐Hispanic White; PC, percent change.

### 3.3 B‐Cell Non‐Hodgkin Lymphoma

From 2000 to 2019, there were 900 791 B‐cell NHL cases. The most common subtype was mature B‐cell NHL (92.35%). The majority of cases were men (55.33%), NHWs (73.95%), and between 70 and 79 years (26.34%) (Table 3). The ASIR per 100 000 population was 51.93 (51.79, 52.08) for men and 33.76 (33.66, 33.87) for women (Table 3, Figures S2–S4).

**TABLE 3:** Counts and age‐standardized rate of B‐cell non‐Hodgkin lymphoma incidence per 100 000 and average annual percent change from 2000 to 2019 in the United States, by age, sex, and race.

Age group (years)	Men			Women		
	Case (%)	ASIR^a^ (95% CI)	AAPC (95% CI)	Case (%)	ASIR (95% CI)	AAPC (95% CI)
Race/ethnicities						
All	498 392 (55.33)	51.93 (51.79, 52.08)	0.37 (0.3, 0.45)	402 399 (44.67)	33.76 (33.66, 33.87)	0.33 (0.2, 0.53)
20–29	7646 (0.85)	3.58 (3.5, 3.66)	0.35 (−0.02, 0.73)	4979 (0.55)	2.42 (2.35, 2.49)	0.39 (−0.12, 0.93)
30–39	15 665 (1.74)	7.73 (7.61, 7.85)	−0.57 (−0.94, −0.21)	10 619 (1.18)	5.25 (5.15, 5.35)	0.39 (−0.08, 0.88)
40–49	39 979 (4.44)	19.3 (19.11, 19.49)	−0.55 (−0.74, −0.37)	27 367 (3.04)	12.94 (12.79, 13.1)	0.49 (0.18, 0.81)
50–59	87 835 (9.75)	47.68 (47.37, 48)	0.05 (−0.12, 0.23)	62 860 (6.98)	32.44 (32.18, 32.69)	0.11 (−0.06, 0.29)
60–69	128 750 (14.29)	106.35 (105.77, 106.93)	0.31 (0.16, 0.49)	96 251 (10.69)	71.09 (70.64, 71.54)	0.31 (0, 0.71)
70–79	129 972 (14.43)	189.02 (187.99, 190.05)	0.73 (0.58, 0.91)	107 328 (11.91)	122.91 (122.17, 123.65)	0.43 (0.27, 0.65)
≥ 80	88 545 (9.83)	247.15 (245.52, 248.79)	0.67 (0.5, 0.85)	92 995 (10.32)	148.31 (147.35, 149.27)	0.23 (0, 0.55)
Hispanic						
All	55 231 (53.54)	42.34 (41.95, 42.73)	0.11 (−0.18, 0.46)	47 937 (46.46)	30.83 (30.55, 31.12)	0.64 (0.02, 1.32)
20–29	2336 (2.26)	3.77 (3.62, 3.93)	1.32 (0.37, 2.39)	1451 (1.41)	2.6 (2.47, 2.74)	1.06 (−0.07, 2.34)
30–39	3878 (3.76)	7.19 (6.96, 7.42)	−0.64 (−1.44, 0.51)	2510 (2.43)	4.95 (4.75, 5.14)	0.74 (−0.12, 1.7)
40–49	7339 (7.11)	16.8 (16.42, 17.19)	−0.81 (−1.24, −0.34)	5186 (5.03)	12.08 (11.75, 12.41)	0.72 (−0.11, 1.69)
50–59	11 283 (10.94)	38.77 (38.06, 39.49)	0.13 (−0.16, 0.48)	9210 (8.93)	30.3 (29.69, 30.93)	0.53 (0.26, 0.84)
60–69	12 886 (12.49)	84.62 (83.16, 86.11)	0.37 (−0.17, 1.16)	11 664 (11.31)	66.01 (64.81, 67.22)	0.38 (−0.04, 0.89)
70–79	11 088 (10.75)	151.78 (148.95, 154.65)	0.19 (−0.28, 0.76)	11 012 (10.67)	112.17 (110.07, 114.29)	0.27 (−0.09, 0.7)
≥ 80	6421 (6.22)	198.2 (193.37, 203.13)	0.14 (−0.44, 0.88)	6904 (6.69)	127.48 (124.49, 130.52)	0.04 (−0.84, 1.13)
NHB						
All	44 502 (51.97)	50.51 (50.01, 51.01)	0.67 (0.55, 0.82)	41 127 (48.03)	34.37 (34.04, 34.71)	0.71 (0.35, 1.12)
20–29	628 (0.73)	3.57 (3.35, 3.81)	−0.8 (−2.1, 0.63)	943 (1.10)	2.31 (2.13, 2.5)	−1.05 (−2.33, 0.29)
30–39	1640 (1.92)	9.2 (8.81, 9.6)	−0.54 (−1.56, 0.46)	2108 (2.46)	6.46 (6.15, 6.78)	0.08 (−0.79, 0.96)
40–49	4206 (4.91)	22.92 (22.31, 23.55)	−0.4 (−0.95, 0.14)	5291 (6.18)	16.18 (15.69, 16.68)	1.16 (0.37, 2)
50–59	8083 (9.44)	53.53 (52.5, 54.58)	−0.34 (−0.85, 0.22)	10 235 (11.95)	36.26 (35.47, 37.06)	0.36 (−0.16, 0.95)
60–69	10 746 (12.55)	109.74 (107.79, 111.72)	1.5 (0.67, 2.38)	12 178 (14.22)	75.26 (73.84, 76.7)	0.88 (0.23, 1.75)
70–79	9644 (11.26)	169.56 (166.08, 173.09)	1.1 (0.39, 2.04)	9182 (10.72)	118.13 (115.77, 120.51)	1.02 (0.39, 1.74)
≥ 80	6180 (7.22)	200.66 (194.86, 206.59)	0.98 (0.49, 1.55)	4565 (5.33)	127.98 (124.8, 131.22)	0.33 (−0.26, 1.01)
NHW						
All	373 621 (56.09)	55.39 (55.21, 55.57)	0.48 (0.35, 0.62)	292 524 (43.91)	35.22 (35.09, 35.35)	0.34 (0.21, 0.49)
20–29	3749 (0.56)	3.56 (3.45, 3.68)	−0.23 (−0.97, 0.52)	2400 (0.36)	2.34 (2.25, 2.44)	−0.12 (−0.84, 0.6)
30–39	8520 (1.28)	8.02 (7.85, 8.2)	−0.78 (−1.18, −0.41)	5524 (0.83)	5.28 (5.14, 5.42)	0.1 (−0.24, 0.44)
40–49	24 950 (3.75)	20.38 (20.13, 20.63)	−0.22 (−0.44, −0.03)	16 084 (2.41)	13.13 (12.93, 13.34)	0.46 (0.08, 0.81)
50–59	61 632 (9.25)	50.74 (50.34, 51.14)	0.21 (0.02, 0.41)	41 776 (6.27)	33.53 (33.21, 33.85)	0.03 (−0.17, 0.24)
60–69	97 064 (14.57)	113.5 (112.78, 114.22)	0.28 (0.1, 0.5)	68 754 (10.32)	74.35 (73.8, 74.91)	0.18 (−0.17, 0.55)
70–79	103 729 (15.57)	202.81 (201.58, 204.05)	0.84 (0.7, 1)	81 569 (12.24)	129.39 (128.51, 130.28)	0.47 (0.33, 0.65)
≥ 80	73 977 (11.11)	264.55 (262.64, 266.46)	0.88 (0.76, 1.02)	76 417 (11.47)	156.62 (155.5, 157.74)	0.44 (0.19, 0.76)

Abbreviations: AAPC, average annual percent change; CI, confidence interval; NHBn non‐Hispanic Black; NHW, non‐Hispanic White.

^a^ The ASIR in the ‘All’ rows refers to the age‐standardized incidence rate. For individual age categories, the values represent age‐specific incidence rates.

From 2000 to 2019, there was an increase in incident cases of B cell NHL in both sexes, reaching a peak at 65–69 and 70–74 in men and women, respectively. Incidence rates increased to reach the highest at the age of 80–84 in both sexes (Figure S5).

### 3.4 Precursor B‐Cell Non‐Hodgkin Lymphoma

A total of 18 786 cases of precursor B‐cell non‐Hodgkin lymphoma (PBNHL) were documented. Most cases were found in males (54.08%), NHWs (54.20%), and in individuals aged 20–29 years (17.04%). The ASIR, per 100 000 population, was recorded at 1.00 (0.98, 1.01) for men and 0.76 (0.75, 0.78) for women (Table 4). Notably, Hispanic men exhibited the highest ASIR among all groups, standing at 1.61 (1.54, 1.67) per 100 000 population (Table 4 and Figures S6–S8).

**TABLE 4:** Counts and age‐standardized rate of precursor non‐Hodgkin lymphoma B‐cell incidence per 100 000 and average annual percent change from 2000 to 2019 in the United States, by age, sex, and race.

Age group (years)	Men			Women		
	Case (%)	ASIR^a^ (95% CI)	AAPC (95% CI)	Case (%)	ASIR (95% CI)	AAPC% (95% CI)
Race/ethnicities						
All	10 159 (54.08)	1 (0.98, 1.01)	0.79 (0.38, 1.24)	8627 (45.92)	0.76 (0.75, 0.78)	0.98 (0.56, 1.43)
20–29	2063 (10.98)	0.97 (0.93, 1.01)	0.99 (0.22, 1.81)	1138 (6.06)	0.56 (0.52, 0.59)	0.76 (−0.26, 1.87)
30–39	1475 (7.85)	0.72 (0.68, 0.76)	1.1 (0.26, 2.02)	1062 (5.65)	0.52 (0.49, 0.55)	1.01 (−0.19, 2.27)
40–49	1560 (8.30)	0.76 (0.72, 0.8)	2.25 (1.18, 3.42)	1257 (6.69)	0.6 (0.57, 0.63)	1.76 (0.71, 2.89)
50–59	1626 (8.66)	0.89 (0.85, 0.94)	0.58 (−0.24, 1.49)	1589 (8.46)	0.82 (0.78, 0.87)	1 (0.01, 2.13)
60–69	1589 (8.46)	1.3 (1.24, 1.37)	0.93 (−0.08, 2.08)	1517 (8.08)	1.11 (1.06, 1.17)	1.41 (0.36, 2.69)
70–79	1163 (6.19)	1.68 (1.58, 1.78)	0.17 (−0.57, 0.95)	1178 (6.27)	1.35 (1.27, 1.43)	0.62 (−1.06, 2.48)
≥ 80	683 (3.64)	1.91 (1.77, 2.06)	−1.73 (−3.53, 0.21)	886 (4.72)	1.41 (1.32, 1.51)	0.61 (−1.8, 2.06)
Hispanic						
All	3335 (55.15)	1.61 (1.54, 1.67)	1.3 (0.56, 2.22)	2712 (44.85)	1.38 (1.32, 1.43)	0.88 (0.07, 1.87)
20–29	1062 (17.56)	1.72 (1.62, 1.82)	2.15 (0.9, 3.59)	560 (9.26)	1.01 (0.92, 1.09)	0.76 (−1.01, 2.77)
30–39	698 (11.54)	1.27 (1.18, 1.37)	0.36 (−0.83, 2.76)	478 (7.90)	0.93 (0.85, 1.01)	0.61 (−1.35, 2.84)
40–49	613 (10.14)	1.4 (1.29, 1.52)	2.16 (−0.09, 4.99)	467 (7.72)	1.09 (0.99, 1.19)	1.43 (−0.36, 3.56)
50–59	434 (7.18)	1.49 (1.35, 1.64)	1.02 (−0.99, 3.62)	470 (7.77)	1.55 (1.41, 1.69)	0.18 (−1.59, 2.41)
60–69	288 (4.76)	1.88 (1.67, 2.12)	0.08 (−2.15, 3.13)	386 (6.38)	2.16 (1.95, 2.39)	1.16 (−0.89, 3.9)
70–79	165 (2.73)	2.23 (1.9, 2.6)	0.35 (−2.88, 4.67)	242 (4.00)	2.45 (2.15, 2.78)	0.09 (−2.37, 3.15)
≥ 80	75 (1.24)	2.31 (1.81, 2.89)	N/A	109 (1.80)	2.01 (1.65, 2.43)	2.52 (−0.66, 7.29)
NHB						
All	621 (50.49)	0.59 (0.54, 0.64)	−0.87 (−2.74, 1.44)	609 (49.51)	0.48 (0.44, 0.52)	0.2 (−1.56, 2.15)
20–29	123 (10.00)	0.46 (0.39, 0.55)	−1.14 (−5.27, 3.41)	70 (5.69)	0.26 (0.2, 0.32)	N/A
30–39	76 (6.18)	0.33 (0.26, 0.41)	−1.05 (−4.54, 2.37)	75 (6.10)	0.29 (0.23, 0.37)	N/A
40–49	124 (10.08)	0.55 (0.45, 0.65)	1.09 (−1.37, 3.67)	88 (7.15)	0.34 (0.27, 0.42)	1.96 (−1.28, 5.11)
50–59	122 (9.92)	0.64 (0.53, 0.77)	N/A	142 (11.54)	0.64 (0.54, 0.75)	0.74 (−3.24, 5.57)
60–69	110 (8.94)	0.96 (0.79, 1.16)	0.61 (−2.71, 5.08)	125 (10.16)	0.87 (0.72, 1.04)	−0.21 (−4.14, 4.74)
70–79	41 (3.33)	0.76 (0.54, 1.03)	N/A	66 (5.37)	0.8 (0.62, 1.02)	N/A
≥ 80	25 (2.03)	1.11 (0.72, 1.64)	N/A	43 (3.50)	0.89 (0.64, 1.2)	−1.93 (−7.6, 4.5)
NHW						
All	5533 (54.34)	0.86 (0.84, 0.89)	0.03 (−0.45, 0.53)	4649 (45.66)	0.64 (0.62, 0.66)	0.35 (−0.24, 0.94)
20–29	741 (7.28)	0.71 (0.66, 0.76)	−1.1 (−2.57, 0.37)	415 (4.08)	0.41 (0.37, 0.45)	−1.21 (−3.21, 0.77)
30–39	573 (5.63)	0.53 (0.49, 0.58)	−0.03 (−1.41, 1.3)	406 (3.99)	0.38 (0.35, 0.42)	1.18 (−0.42, 2.83)
40–49	724 (7.11)	0.6 (0.56, 0.65)	1.43 (0.3, 2.56)	576 (5.66)	0.48 (0.44, 0.52)	0.75 (−0.8, 2.26)
50–59	957 (9.40)	0.8 (0.75, 0.85)	0.1 (−1.33, 1.61)	872 (8.56)	0.7 (0.66, 0.75)	0.4 (−0.84, 1.7)
60–69	1102 (10.82)	1.28 (1.21, 1.36)	0.78 (−0.35, 2.06)	887 (8.71)	0.95 (0.89, 1.02)	1.16 (−0.42, 3.08)
70–79	882 (8.66)	1.71 (1.6, 1.83)	0.19 (−0.98, 1.48)	794 (7.80)	1.26 (1.17, 1.35)	0.42 (−1.25, 2.26)
≥ 80	554 (5.44)	1.98 (1.82, 2.15)	−1.51 (−3.24, 0.3)	699 (6.87)	1.43 (1.33, 1.54)	0.07 (−2.44, 1.82)

Abbreviations: AAPC, average annual percent change; CI, confidence interval; N/A, not available; NHB, non‐Hispanic Black; NHW, non‐Hispanic White.

^a^ The ASIR in the ‘All’ rows refers to the age‐standardized incidence rate. For individual age categories, the values represent age‐specific incidence rates.

There was a reduction in the incident cases of PBNHL among men from 20–24 to 30–34, followed by a minimal increase to 60–64, after which there was a decline. For women, there was an overall increase from 25–29 to 55–59, which was preceded by a decline to 80–84 (Figure S9).

### 3.5 Mature B‐Cell Non‐Hodgkin Lymphoma

Overall, 831 875 cases of mature B‐cell Non‐Hodgkin lymphoma (MBNHL) were reported. The majority were in men (55.53%), NHWs (74.31%), and aged 70–79 years (26.61%) (Table 5). Both sexes experienced a significant incline in ASIRs (per 100 000 population) over 2000–2019 (AAPC: 0.37% [0.29, 0.45] for men, and AAPC: 0.30% [0.19, 0.46] for women). NHW men had the highest ASIR per 100 000 population (51.52 [51.35, 51.69]) (Table 5 and Figures S10–S12).

**TABLE 5:** Counts and age‐standardized rate of mature B‐cell non‐Hodgkin lymphoma incidence per 100 000 and average annual percent change from 2000 to 2019 in the United States, by age, sex, and race.

Age group (years)	Men			Women		
	Case (%)	ASIR^a^ (95% CI)	AAPC (95% CI)	Case (%)	ASIR (95% CI)	AAPC (95% CI)
Race/ethnicities						
All	461 958 (55.53)	48.11 (47.97, 48.26)	0.37 (0.29, 0.45)	369 917 (44.47)	31.01 (30.91, 31.12)	0.3 (0.19, 0.46)
20–29	5136 (0.62)	2.4 (2.34, 2.47)	0.14 (−0.29, 0.59)	3524 (0.42)	1.71 (1.66, 1.77)	0.33 (−0.33, 1.02)
30–39	13 267 (1.59)	6.56 (6.45, 6.67)	−0.71 (−1.15, −0.28)	8980 (1.08)	4.45 (4.35, 4.54)	0.26 (−0.15, 0.67)
40–49	36 448 (4.38)	17.58 (17.4, 17.76)	−0.51 (−0.75, −0.24)	24 847 (2.99)	11.74 (11.6, 11.89)	0.42 (0.08, 0.76)
50–59	82 410 (9.91)	44.72 (44.42, 45.03)	0.04 (−0.15, 0.24)	58 310 (7.01)	30.09 (29.84, 30.33)	0.12 (−0.08, 0.34)
60–69	121 298 (14.58)	100.2 (99.63, 100.76)	0.42 (0.28, 0.6)	89 803 (10.80)	66.32 (65.89, 66.76)	0.3 (0.09, 0.6)
70–79	121 720 (14.63)	176.99 (175.99, 177.99)	0.7 (0.54, 0.88)	99 670 (11.98)	114.13 (113.42, 114.84)	0.4 (0.25, 0.58)
≥ 80	81 679 (9.82)	227.93 (226.36, 229.5)	0.67 (0.45, 0.93)	84 783 (10.19)	135.3 (134.38, 136.21)	0.19 (−0.05, 0.51)
Hispanic						
All	48 923 (53.54)	38.33 (37.96, 38.71)	0.08 (−0.24, 0.46)	42 447 (46.46)	27.59 (27.32, 27.87)	0.56 (0.03, 1.16)
20–29	1178 (1.29)	1.9 (1.79, 2.01)	0.73 (−0.93, 2.55)	825 (0.90)	1.48 (1.38, 1.58)	1.36 (−0.55, 3.56)
30–39	2939 (3.22)	5.47 (5.27, 5.67)	−0.58 (−1.57, 0.47)	1908 (2.09)	3.77 (3.61, 3.95)	0.72 (−0.53, 2.14)
40–49	6290 (6.88)	14.4 (14.05, 14.76)	−0.93 (−1.34, −0.5)	4457 (4.88)	10.38 (10.08, 10.69)	0.58 (−0.16, 1.45)
50–59	10 364 (11.34)	35.61 (34.93, 36.31)	0.1 (−0.28, 0.56)	8291 (9.07)	27.28 (26.69, 27.87)	0.55 (0.1, 1.09)
60–69	11 964 (13.09)	78.61 (77.19, 80.04)	0.45 (−0.09, 1.2)	10 629 (11.63)	60.15 (59.01, 61.31)	0.36 (−0.02, 0.8)
70–79	10 316 (11.29)	141.19 (138.46, 143.96)	0.22 (−0.23, 0.75)	10 075 (11.03)	102.59 (100.59, 104.62)	0.64 (0.05, 1.26)
≥ 80	5872 (6.43)	181.15 (176.53, 185.86)	0.06 (−0.42, 0.67)	6262 (6.85)	115.63 (112.79, 118.53)	−0.12 (−0.88, 0.83)
NHB						
All	41 907 (52.00)	47.71 (47.22, 48.2)	0.75 (0.54, 1.01)	38 681 (48.00)	32.37 (32.04, 32.7)	0.73 (0.4, 1.09)
20–29	731 (0.91)	2.77 (2.57, 2.98)	−0.67 (−2.98, 1.85)	508 (0.63)	1.87 (1.71, 2.04)	−1.19 (−2.89, 0.57)
30–39	1861 (2.31)	8.13 (7.76, 8.5)	−0.49 (−1.64, 0.67)	1448 (1.80)	5.71 (5.42, 6.01)	0.03 (−0.88, 0.94)
40–49	4882 (6.06)	21.14 (20.55, 21.74)	−0.22 (−0.84, 0.42)	3899 (4.84)	15 (14.53, 15.47)	1.18 (0.51, 1.89)
50–59	9683 (12.02)	50.63 (49.63, 51.65)	−0.25 (−0.9, 0.47)	7580 (9.41)	34 (33.24, 34.78)	0.39 (−0.12, 0.95)
60–69	11 626 (14.43)	104.82 (102.92, 106.76)	1.47 (0.53, 2.43)	10 181 (12.63)	71.3 (69.92, 72.7)	0.84 (0.12, 1.82)
70–79	8806 (10.93)	162.62 (159.21, 166.07)	1.09 (0.49, 1.88)	9208 (11.43)	112.78 (110.48, 115.11)	1.01 (0.39, 1.74)
≥ 80	4318 (5.36)	189.71 (184.07, 195.48)	0.98 (0.4, 1.66)	5857 (7.27)	121.35 (118.25, 124.5)	0.37 (−0.16, 0.96)
NHW						
All	348 263 (56.34)	51.52 (51.35, 51.69)	0.47 (0.37, 0.57)	269 875 (43.66)	32.47 (32.35, 32.6)	0.33 (0.2, 0.49)
20–29	2778 (0.45)	2.64 (2.54, 2.74)	0 (−0.81, 0.85)	1818 (0.29)	1.77 (1.69, 1.86)	0.06 (−0.64, 0.77)
30–39	7497 (1.21)	7.07 (6.91, 7.23)	−0.78 (−1.2, −0.39)	4834 (0.78)	4.62 (4.49, 4.76)	−0.02 (−0.35, 0.29)
40–49	23 100 (3.74)	18.85 (18.61, 19.1)	−0.17 (−0.37, 0.04)	14 815 (2.40)	12.08 (11.89, 12.28)	0.46 (0.02, 0.88)
50–59	58 036 (9.39)	47.76 (47.37, 48.15)	0.21 (0.01, 0.42)	38 945 (6.30)	31.26 (30.95, 31.58)	0.06 (−0.14, 0.25)
60–69	91 543 (14.81)	107.04 (106.35, 107.74)	0.42 (0.27, 0.61)	64 284 (10.40)	69.52 (68.98, 70.06)	0.2 (−0.3, 0.65)
70–79	97 092 (15.71)	189.81 (188.61, 191.01)	0.88 (0.73, 1.03)	75 680 (12.24)	120.05 (119.2, 120.91)	0.45 (0.29, 0.64)
≥ 80	68 217 (11.04)	243.9 (242.07, 245.74)	0.83 (0.63, 1.06)	69 499 (11.24%)	142.53 (141.46, 143.6)	0.4 (0.16, 0.7)

Abbreviations: AAPC, average annual percent change; CI, confidence interval; NHB, non‐Hispanic Black; NHW, non‐Hispanic White.

^a^ The ASIR in the ‘All’ rows refers to the age‐standardized incidence rate. For individual age categories, the values represent age‐specific incidence rates.

Among men, the incident cases and rates peaked at 65–69 and 80–84 age groups, respectively. For women, the cases and rates peaked at 70–74 and 80–84, respectively. Men had a higher incidence rate compared to women (Figure S13).

### 3.6 B‐Cell Non‐Hodgkin Lymphoma Not Otherwise Specified

There were 50 130 B‐cell non‐Hodgkin lymphoma not otherwise specified (BNHL‐NOS), mostly in men (52.41%), NHWs (75.45%), and aged 80+ years (28.75%). NHW men had the highest ASIR per 100 000 population among other groups (3.00 [2.96, 3.05]) (Table 6 and Figures S14–S16).

**TABLE 6:** Counts and age‐standardized rate of B‐cell non‐Hodgkin lymphoma not otherwise specified (NOS) incidence per 100 000 and average annual percent change from 2000 to 2019 in the United States, by age, sex, and race.

Age group (years)	Men			Women		
	Case (%)	ASIR^a^ (95% CI)	AAPC (95% CI)	Case (%)	ASIR (95% CI)	AAPC (95% CI)
Race/ethnicities						
All	26 275 (52.41)	2.82 (2.79, 2.86)	−0.1 (−0.81, 0.5)	23 855 (47.59)	1.99 (1.96, 2.01)	0.4 (−0.54, 1.36)
20–29	447 (0.89)	0.21 (0.19, 0.23)	−1.69 (−3.41, 0.34)	317 (0.63)	0.15 (0.14, 0.17)	−0.58 (−2.53, 1.45)
30–39	923 (1.84)	0.45 (0.43, 0.49)	−1.63 (−3.19, 0.15)	577 (1.15)	0.28 (0.26, 0.31)	1.31 (0.05, 2.64)
40–49	1971 (3.93)	0.95 (0.91, 1)	−2.1 (−3.22, −1.06)	1263 (2.52)	0.6 (0.57, 0.63)	0.65 (−0.52, 1.86)
50–59	3799 (7.58)	2.07 (2, 2.13)	−0.43 (−2.14, 1.8)	2961 (5.91)	1.53 (1.47, 1.58)	−0.55 (−1.47, 0.41)
60–69	5863 (11.70)	4.85 (4.73, 4.98)	−0.24 (−1.42, 0.9)	4931 (9.84)	3.65 (3.55, 3.76)	−0.52 (−1.15, 0.16)
70–79	7089 (14.14)	10.35 (10.11, 10.6)	0.27 (−0.2, 0.72)	6480 (12.93)	7.43 (7.25, 7.61)	0.59 (0.03, 1.28)
≥ 80	6183 (12.33)	17.32 (16.89, 17.76)	2.2 (0.47, 3.95)	7326 (14.61)	11.61 (11.34, 11.88)	0.14 (−0.74, 1.05)
Hispanic						
All	2973 (51.70)	2.4 (2.31, 2.5)	−0.25 (−1.82, 2.05)	2778 (48.30)	1.86 (1.79, 1.93)	0.33 (−1.04, 2.91)
20–29	96 (1.67)	0.15 (0.13, 0.19)	−0.79 (−5.07, 3.93)	66 (1.15)	0.12 (0.09, 0.15)	N/A
30–39	241 (4.19)	0.45 (0.39, 0.51)	−0.99 (−3.61, 1.83)	124 (2.16)	0.24 (0.2, 0.29)	0.46 (−3.43, 5.11)
40–49	436 (7.58)	1 (0.91, 1.1)	−3.26 (−5.39, −1.09)	262 (4.56)	0.61 (0.54, 0.69)	1.37 (−0.72, 3.93)
50–59	485 (8.43)	1.67 (1.52, 1.82)	−0.05 (−1.41, 1.63)	449 (7.81)	1.48 (1.34, 1.62)	0.32 (−0.89, 1.85)
60–69	634 (11.02)	4.13 (3.82, 4.47)	−1.19 (−2.84, 0.87)	649 (11.28)	3.69 (3.41, 3.99)	2.43 (−0.37, 5.31)
70–79	607 (10.55)	8.37 (7.71, 9.06)	−0.83 (−3.12, 2.39)	695 (12.08)	7.12 (6.6, 7.68)	−0.09 (−2.09, 2.3)
≥ 80	474 (8.24)	14.75 (13.45, 16.14)	1.21 (−1.81, 5.58)	533 (9.27)	9.83 (9.01, 10.7)	1.16 (−1.8, 5.3)
NHB						
All	1974 (51.80)	2.21 (2.11, 2.32)	0.04 (−0.96, 1.16)	1837 (48.20)	1.53 (1.46, 1.6)	0.64 (−0.92, 2.6)
20–29	89 (2.34)	0.34 (0.27, 0.41)	−1.63 (−6.09, 3.12)	50 (1.31)	0.18 (0.14, 0.24)	N/A
30–39	171 (4.49)	0.74 (0.64, 0.86)	−1.24 (−4.79, 2.34)	117 (3.07)	0.45 (0.38, 0.54)	1.67 (−4.11, 7.95)
40–49	285 (7.48)	1.24 (1.1, 1.39)	−4.24 (−7.25, −1.69)	219 (5.75)	0.85 (0.74, 0.97)	0.74 (−1.96, 3.6)
50–59	430 (11.28)	2.26 (2.05, 2.48)	−1.67 (−4.14, 0.99)	361 (9.47)	1.62 (1.46, 1.8)	−0.56 (−2.65, 1.85)
60–69	442 (11.60)	3.95 (3.59, 4.34)	1.11 (−1.81, 5.98)	440 (11.55)	3.09 (2.81, 3.39)	0.71 (−1.35, 3.34)
70–79	335 (8.79)	6.19 (5.54, 6.89)	1.25 (−0.67, 3.67)	370 (9.71)	4.55 (4.09, 5.03)	1.02 (−2.66, 5.57)
≥ 80	222 (5.83)	9.85 (8.59, 11.24)	1.51 (−1.16, 5.04)	280 (7.35)	5.74 (5.09, 6.46)	−1.8 (−4.24, 1.02)
NHW						
All	19 825 (52.41)	3 (2.96, 3.05)	0.01 (−0.6, 0.54)	18 000 (47.59)	2.11 (2.08, 2.14)	0.38 (−0.07, 0.83)
20–29	230 (0.61)	0.22 (0.19, 0.25)	−1.12 (−3.55, 1.59)	167 (0.44)	0.16 (0.14, 0.19)	0.57 (−2.54, 3.85)
30–39	450 (1.19)	0.42 (0.39, 0.46)	−1.72 (−4.04, 0.36)	284 (0.75)	0.27 (0.24, 0.3)	0.84 (−0.73, 2.45)
40–49	1126 (2.98)	0.93 (0.87, 0.98)	−1.54 (−3, −0.24)	693 (1.83)	0.57 (0.53, 0.61)	0.19 (−1.24, 1.54)
50–59	2639 (6.98)	2.18 (2.1, 2.27)	−0.27 (−2.69, 2.71)	1959 (5.18)	1.57 (1.5, 1.64)	−0.75 (−1.83, 0.34)
60–69	4419 (11.68)	5.18 (5.03, 5.33)	−0.39 (−1.26, 0.61)	3583 (9.47)	3.88 (3.76, 4.01)	−1.52 (−2.25, −0.89)
70–79	5755 (15.21)	11.29 (11, 11.59)	0.46 (−0.21, 1.09)	5095 (13.47)	8.09 (7.86, 8.31)	0.95 (0.39, 1.62)
≥ 80	5206 (13.76)	18.67 (18.16, 19.18)	2.17 (0.46, 4.01)	6219 (16.44)	12.66 (12.35, 12.98)	0.42 (−0.5, 1.33)

Abbreviations: AAPC, average annual percent change; CI, confidence interval; N/A, not available; NHB, non‐Hispanic Black; NHW, non‐Hispanic White.

^a^ The ASIR in the ‘All’ rows refers to the age‐standardized incidence rate. For individual age categories, the values represent age‐specific incidence rates.

The incident cases of BNHL‐NOS increased to 75–79 age groups in men, followed by a slight decrease. Women followed a similar pattern; however, the incidence of BNHL‐NOS peaked in the 85+ group (Figure S17).

### 3.7 T‐Cell Non‐Hodgkin Lymphoma

There were 60 259 T‐cell NHL, mostly in men (57.93%), NHWs (63.93%), and between 60 and 69 years (22.15%). NHB men had the highest ASIR per 100 000 population (4.77 [4.62, 4.92]) and the greatest AAPC (2.99% [1.50, 4.92]) (Table 7 and Figures S18–S20).

**TABLE 7:** Counts and age‐standardized rate of T‐cell non‐Hodgkin lymphoma incidence per 100 000 and average annual percent change from 2000 to 2019 in the United States, by age, sex, and race.

Age group (years)	Men			Women		
	Case (%)	ASIR^a^ (95% CI)	AAPC (95% CI)	Case (%)	ASIR (95% CI)	AAPC (95% CI)
Race/ethnicities						
All	34 911 (57.93)	3.54 (3.51, 3.58)	1.89 (1.22, 2.57)	25 348 (42.07)	2.19 (2.16, 2.22)	1.88 (1.16, 2.66)
20–29	2098 (3.48)	0.98 (0.94, 1.03)	5.38 (4.14, 7.23)	1304 (2.16)	0.63 (0.6, 0.67)	3.25 (0.39, 6.64)
30–39	2820 (4.68)	1.38 (1.33, 1.43)	1.89 (0.74, 3.14)	2007 (3.33)	0.98 (0.94, 1.03)	2.49 (1.39, 3.69)
40–49	4297 (7.13)	2.09 (2.03, 2.15)	0.95 (0.21, 1.7)	3055 (5.07)	1.46 (1.41, 1.51)	1.93 (1.21, 2.68)
50–59	6479 (10.75)	3.54 (3.45, 3.62)	0.99 (0.38, 1.68)	4786 (7.94)	2.48 (2.41, 2.56)	2.18 (1.04, 3.51)
60–69	7882 (13.08)	6.49 (6.35, 6.64)	0.86 (0.22, 1.61)	5464 (9.07)	4.02 (3.92, 4.13)	1.49 (0.72, 2.43)
70–79	7049 (11.70)	10.23 (9.99, 10.47)	1.98 (1.07, 3.13)	4994 (8.29)	5.71 (5.56, 5.87)	2 (1.45, 2.65)
≥ 80	4286 (7.11)	11.95 (11.6, 12.32)	1.38 (−0.17, 3.63)	3738 (6.20)	6.01 (5.82, 6.21)	2.16 (1.43, 2.98)
Hispanic						
All	4634 (57.50)	3.02 (2.93, 3.12)	2.7 (2.1, 3.58)	3425 (42.50)	1.94 (1.88, 2.01)	1.41 (0.42, 2.67)
20–29	545 (6.76)	0.88 (0.81, 0.96)	5.13 (2.35, 9.77)	349 (4.33)	0.63 (0.56, 0.7)	1.58 (−0.63, 4.19)
30–39	622 (7.72)	1.14 (1.05, 1.23)	1.13 (−0.4, 2.83)	466 (5.78)	0.91 (0.83, 0.99)	2.55 (1.05, 4.33)
40–49	797 (9.89)	1.82 (1.7, 1.95)	0.95 (−0.09, 2.14)	531 (6.59)	1.24 (1.13, 1.35)	0.99 (−1.32, 3.74)
50–59	856 (10.62)	2.94 (2.75, 3.14)	1.13 (−0.35, 2.98)	698 (8.66)	2.3 (2.13, 2.48)	0.96 (−0.78, 3.2)
60–69	827 (10.26)	5.41 (5.05, 5.8)	1.59 (0, 3.79)	589 (7.31)	3.33 (3.07, 3.61)	0.96 (−1.03, 3.7)
70–79	646 (8.02)	8.77 (8.1, 9.48)	−0.3 (−2.21, 3.51)	503 (6.24)	5.1 (4.66, 5.57)	0.94 (−1.2, 3.72)
≥ 80	341 (4.23)	10.49 (9.41, 11.67)	−0.44 (−2.82, 2.83)	289 (3.59)	5.33 (4.74, 5.98)	2.17 (−0.21, 5.73)
NHB						
All	4623 (51.89)	4.77 (4.62, 4.92)	2.99 (1.5, 4.92)	4287 (48.11)	3.46 (3.35, 3.56)	2.27 (1.06, 3.81)
20–29	293 (3.29)	1.11 (0.98, 1.24)	7.39 (2.89, 14.6)	246 (2.76)	0.9 (0.79, 1.02)	1.98 (−1.16, 5.78)
30–39	532 (5.97)	2.32 (2.13, 2.53)	1.73 (−0.9, 4.62)	462 (5.19)	1.81 (1.65, 1.98)	1.63 (−0.65, 4.19)
40–49	823 (9.24)	3.58 (3.34, 3.83)	1.21 (−0.8, 3.4)	753 (8.45)	2.92 (2.71, 3.13)	1.49 (0.19, 2.87)
50–59	1082 (12.14)	5.68 (5.35, 6.03)	1.58 (0.91, 2.34)	972 (10.91)	4.38 (4.11, 4.67)	3.28 (1.75, 5.24)
60–69	993 (11.14)	8.82 (8.27, 9.39)	1.87 (−0.05, 4.44)	875 (9.82)	6.07 (5.67, 6.49)	2.58 (0.98, 5.38)
70–79	631 (7.08)	11.63 (10.73, 12.58)	4.04 (1.99, 6.54)	619 (6.95)	7.56 (6.97, 8.18)	2.99 (0.97, 6.36)
≥ 80	269 (3.02)	11.75 (10.39, 13.25)	4.94 (0.91, 10.55)	360 (4.04)	7.46 (6.71, 8.28)	1.86 (−0.67, 5.11)
NHW						
All	22 881 (59.39)	3.46 (3.41, 3.5)	1.18 (0.61, 1.72)	15 644 (40.61)	2.02 (1.99, 2.05)	1.95 (1.51, 2.44)
20–29	1022 (2.65)	0.97 (0.91, 1.03)	2.77 (0.17, 5.61)	543 (1.41)	0.53 (0.49, 0.58)	3.89 (0.06, 8.14)
30–39	1349 (3.50)	1.26 (1.2, 1.33)	1.61 (0.34, 2.77)	835 (2.17)	0.79 (0.74, 0.85)	2.63 (1.11, 4.27)
40–49	2284 (5.93)	1.89 (1.81, 1.97)	0.83 (−0.18, 1.79)	1494 (3.88)	1.23 (1.17, 1.3)	2.28 (1.41, 3.17)
50–59	4010 (10.41)	3.32 (3.22, 3.43)	0.64 (−0.03, 1.32)	2744 (7.12)	2.22 (2.13, 2.3)	1.88 (0.44, 3.51)
60–69	5473 (14.21)	6.39 (6.22, 6.56)	0.68 (−0.13, 1.58)	3609 (9.37)	3.9 (3.77, 4.03)	0.99 (0.15, 1.96)
70–79	5312 (13.79)	10.37 (10.1, 10.66)	2.36 (0.87, 3.82)	3531 (9.17)	5.6 (5.42, 5.79)	1.99 (1.58, 2.44)
≥ 80	3431 (8.91)	12.26 (11.86, 12.68)	1.62 (−0.49, 4.64)	2888 (7.50)	5.98 (5.77, 6.21)	2.16 (1.3, 3.11)

Abbreviations: AAPC, average annual percent change; CI, confidence interval; NHB, non‐Hispanic Black; NHW, non‐Hispanic White.

^a^ The ASIR in the ‘All’ rows refers to the age‐standardized incidence rate. For individual age categories, the values represent age‐specific incidence rates.

There was a marked exponential rise in T‐cell NHL incidence for both sexes, reaching its highest levels in the 65–69 age group, after which a decline was observed (Figure S21).

### 3.8 Precursor T‐Cell Non‐Hodgkin Lymphoma

From 2000 to 2019, there were 3223 cases of precursor T‐cell non‐Hodgkin lymphoma (PTNHL) in the US. It mostly affected men (67.83%), NHWs (56.22%), and those aged 20–29 years (30.04%) (Table 8). NHB men had the highest ASIR per 100 000 population (0.30 [0.27, 0.34]). The AAPCs for men and women were 12.33% (2.39, 22.68) and 10.50% (1.89, 21.69), respectively (Table 8 and Figures S22–S24).

**TABLE 8:** Counts and age‐standardized rate of precursor T‐cell non‐Hodgkin lymphoma incidence per 100 000 and average annual percent change from 2000 to 2019 in the United States, by age, sex, and race.

Age group (years)	Men			Women		
	Case (%)	ASIR^a^ (95% CI)	AAPC (95% CI)	Case (%)	ASIR (95% CI)	AAPC (95% CI)
Race/ethnicities						
All	2186 (67.83)	0.21 (0.2, 0.22)	12.33 (2.39, 22.68)	1037 (32.17)	0.09 (0.09, 0.1)	10.5 (1.89, 21.69)
20–29	717 (22.25)	0.34 (0.31, 0.36)	8.6 (1.47, 18.63)	251 (7.79)	0.12 (0.11, 0.14)	N/A
30–39	459 (14.24)	0.22 (0.2, 0.24)	14.74 (2.86, 28.97)	194 (6.02)	0.09 (0.08, 0.11)	N/A
40–49	314 (9.74)	0.15 (0.14, 0.17)	1.51 (−1.51, 4.85)	168 (5.21)	0.08 (0.07, 0.09)	4.67 (2.07, 7.91)
50–59	246 (7.63)	0.14 (0.12, 0.15)	4.94 (3.01, 7.47)	146 (4.53)	0.08 (0.06, 0.09)	N/A
60–69	214 (6.64)	0.18 (0.15, 0.2)	1.64 (−3.11, 16.11)	119 (3.69)	0.09 (0.07, 0.1)	N/A
70–79	153 (4.75)	0.22 (0.19, 0.26)	N/A	96 (2.98)	0.11 (0.09, 0.13)	N/A
≥ 80	83 (2.58)	0.23 (0.18, 0.28)	N/A	63 (1.95)	0.1 (0.08, 0.13)	N/A
Hispanic						
All	411 (68.73)	0.17 (0.15, 0.19)	17.19 (5.09, 29.93)	187 (31.27)	0.09 (0.07, 0.1)	11.62 (3.47, 23.26)
20–29	183 (30.60)	0.3 (0.25, 0.34)	N/A	58 (9.70)	0.1 (0.08, 0.13)	N/A
30–39	101 (16.89)	0.18 (0.14, 0.22)	N/A	45 (7.53)	0.08 (0.06, 0.11)	N/A
40–49	49 (8.19)	0.11 (0.08, 0.15)	N/A	30 (5.02)	0.07 (0.05, 0.1)	N/A
50–59	39 (6.52)	0.13 (0.1, 0.18)	N/A	22 (3.68)	0.07 (0.05, 0.11)	N/A
60–69	24 (4.01)	0.15 (0.1, 0.23)	N/A	15 (2.51)	0.08 (0.05, 0.14)	N/A
70–79	10 (1.67)	0.13 (0.06, 0.24)	N/A	12 (2.01)	0.12 (0.06, 0.21)	N/A
≥ 80	5 (0.84)	0.15 (0.05, 0.35)	N/A	5 (0.84)	0.09 (0.03, 0.22)	N/A
NHB						
All	332 (64.97)	0.3 (0.27, 0.34)	4.77 (1.12, 14.33)	179 (35.03)	0.14 (0.12, 0.16)	N/A
20–29	100 (19.57)	0.37 (0.3, 0.45)	N/A	41 (8.02)	0.15 (0.11, 0.2)	N/A
30–39	60 (11.74)	0.26 (0.2, 0.33)	N/A	31 (6.07)	0.12 (0.08, 0.17)	N/A
40–49	54 (10.57)	0.24 (0.18, 0.31)	N/A	29 (5.68)	0.11 (0.08, 0.16)	N/A
50–59	51 (9.98)	0.27 (0.2, 0.35)	N/A	29 (5.68)	0.13 (0.09, 0.19)	N/A
60–69	40 (7.83)	0.35 (0.25, 0.48)	N/A	25 (4.89)	0.17 (0.11, 0.26)	N/A
70–79	15 (2.94)	0.29 (0.16, 0.48)	N/A	18 (3.52)	0.22 (0.13, 0.34)	N/A
≥ 80	12 (2.35)	0.51 (0.27, 0.9)	N/A	6 (1.17)	0.13 (0.05, 0.27)	N/A
NHW						
All	1229 (67.83)	0.21 (0.19, 0.22)	10.71 (1.47, 19.64)	583 (32.17)	0.09 (0.08, 0.1)	12.01 (3.98, 20.85)
20–29	357 (19.70)	0.34 (0.31, 0.38)	2.65 (−0.7, 6.73)	131 (7.23)	0.13 (0.11, 0.15)	N/A
30–39	242 (13.36)	0.22 (0.2, 0.25)	8.31 (−0.09, 22.16)	97 (5.35)	0.09 (0.07, 0.11)	N/A
40–49	187 (10.32)	0.16 (0.14, 0.18)	−0.01 (−4.35, 4.07)	96 (5.30)	0.08 (0.06, 0.1)	9.6 (5.4, 17.25)
50–59	135 (7.45)	0.11 (0.09, 0.13)	N/A	85 (4.69)	0.07 (0.06, 0.09)	N/A
60–69	136 (7.51)	0.16 (0.13, 0.19)	N/A	69 (3.81)	0.07 (0.06, 0.09)	N/A
70–79	110 (6.07)	0.21 (0.18, 0.26)	N/A	57 (3.15)	0.09 (0.07, 0.12)	N/A
≥ 80	62 (3.42)	0.22 (0.17, 0.28)	N/A	48 (2.65)	0.1 (0.07, 0.13)	N/A

Abbreviations: AAPC, average annual percent change; CI, confidence interval; N/A not available; NHB, non‐Hispanic Black; NHW, non‐Hispanic White.

^a^ The ASIR in the ‘All’ rows refers to the age‐standardized incidence rate. For individual age categories, the values represent age‐specific incidence rates.

There was almost a decrease in the incident cases of PTNHL in both sexes. PTNHL incidence rates declined between 20–24 and 60–64, after which they increased to the 80–84 age group. For women, the incidence rates had minimal fluctuations (Figure S25).

### 3.9 Mature T‐Cell Non‐Hodgkin Lymphoma

From 2000 to 2019, there were 57 036 cases of mature T‐cell non‐Hodgkin lymphoma (MTNHL) reported in the US, with a higher incidence among men (57.38%), NHWs (64.37%), and those aged 60–69 years (22.81%) (Table 9). The ASIR per 100 000 people was 3.33 (3.30, 3.37) for men and 2.10 (2.07, 2.12) for women. NHB men had the highest ASIR per 100 000 population (4.47 [4.33, 4.61]) (Table 9). The AAPCs for men and women were 1.11% (0.60, 1.63) and 1.74% (0.79, 2.94), respectively (Table 9 and Figures S26–S28).

**TABLE 9:** Counts and age‐standardized rate of mature T‐cell non‐Hodgkin lymphoma incidence per 100 000 and average annual percent change from 2000 to 2019 in the United States, by age, sex, and race.

Age group (years)	Men			Women		
	Case (%)	ASIR^a^ (95 CI)	AAPC (95 CI)	Case (%)	ASIR (95% CI)	AAPC (95% CI)
Race/ethnicities						
All	32 725 (57.38)	3.33 (3.3, 3.37)	1.11 (0.6, 1.63)	24 311 (42.62)	2.1 (2.07, 2.12)	1.74 (0.79, 2.94)
20–29	1381 (2.42)	0.65 (0.61, 0.68)	2.46 (1.29, 3.76)	1053 (1.85)	0.51 (0.48, 0.54)	0.64 (−1.01, 3.28)
30–39	2361 (4.14)	1.16 (1.11, 1.21)	1.23 (0.07, 2.44)	1813 (3.18)	0.89 (0.85, 0.93)	2.05 (0.8, 3.44)
40–49	3983 (6.98)	1.93 (1.87, 2)	0.81 (−0.06, 1.7)	2887 (5.06)	1.38 (1.33, 1.43)	1.76 (1.03, 2.53)
50–59	6233 (10.93)	3.4 (3.32, 3.49)	0.82 (0.23, 1.49)	4640 (8.14)	2.41 (2.34, 2.48)	2.09 (1.01, 3.35)
60–69	7668 (13.44)	6.32 (6.18, 6.46)	0.97 (0.24, 1.95)	5345 (9.37)	3.94 (3.83, 4.04)	1.47 (0.69, 2.45)
70–79	6896 (12.09)	10.01 (9.77, 10.25)	1.88 (1.14, 2.78)	4898 (8.59)	5.6 (5.45, 5.76)	1.94 (1.37, 2.62)
≥ 80	4203 (7.37)	11.72 (11.37, 12.08)	1.43 (−0.06, 3.62)	3675 (6.44)	5.91 (5.72, 6.1)	2.05 (1.33, 2.86)
Hispanic						
All	4223 (56.60)	2.85 (2.76, 2.95)	2.3 (1.56, 3.36)	3238 (43.40)	1.86 (1.79, 1.93)	1.33 (0.4, 2.5)
20–29	362 (4.85)	0.58 (0.53, 0.65)	3.58 (−1.89, 13.21)	291 (3.90)	0.52 (0.46, 0.59)	1.42 (−1.31, 4.71)
30–39	521 (6.98)	0.96 (0.88, 1.04)	0.36 (−1.29, 2.12)	421 (5.64)	0.82 (0.74, 0.9)	2.26 (0.72, 4.09)
40–49	748 (10.03)	1.71 (1.59, 1.84)	0.61 (−0.71, 2.12)	501 (6.71)	1.17 (1.07, 1.27)	0.84 (−1.78, 4.02)
50–59	817 (10.95)	2.81 (2.62, 3.01)	0.89 (−0.46, 2.52)	676 (9.06)	2.23 (2.06, 2.4)	0.97 (−0.94, 3.42)
60–69	803 (10.76)	5.26 (4.9, 5.64)	1.55 (−0.09, 3.82)	574 (7.69)	3.25 (2.99, 3.53)	0.97 (−1.14, 3.89)
70–79	636 (8.52)	8.64 (7.98, 9.34)	−0.49 (−2.48, 3.53)	491 (6.58)	4.98 (4.55, 5.44)	0.77 (−1.6, 3.8)
≥ 80	336 (4.50)	10.34 (9.26, 11.52)	−0.51 (−2.89, 2.74)	284 (3.81)	5.24 (4.65, 5.89)	2.06 (−0.49, 5.91)
NHB						
All	4291 (51.09)	4.47 (4.33, 4.61)	2.69 (1.77, 4.08)	4108 (48.91)	3.32 (3.22, 3.42)	2.02 (0.93, 3.34)
20–29	193 (2.30)	0.73 (0.63, 0.84)	1.73 (−1.17, 5.5)	205 (2.44)	0.75 (0.65, 0.86)	1.02 (−2.31, 4.9)
30–39	472 (5.62)	2.06 (1.88, 2.26)	1.2 (−1.59, 4.23)	431 (5.13)	1.68 (1.53, 1.85)	1.2 (−1.3, 3.97)
40–49	769 (9.16)	3.34 (3.11, 3.59)	0.86 (−0.91, 2.75)	724 (8.62)	2.81 (2.6, 3.02)	1.41 (−0.03, 2.94)
50–59	1031 (12.28)	5.41 (5.09, 5.76)	1.48 (0.84, 2.22)	943 (11.23)	4.25 (3.98, 4.53)	1.95 (−0.39, 7.47)
60–69	953 (11.35)	8.46 (7.93, 9.02)	1.69 (−0.22, 4.19)	850 (10.12)	5.9 (5.51, 6.31)	2.59 (1.09, 4.89)
70–79	616 (7.33)	11.34 (10.46, 12.28)	2.41 (0.95, 4.28)	601 (7.16)	7.34 (6.76, 7.95)	2.88 (0.54, 7.7)
≥ 80	257 (3.06)	11.24 (9.9, 12.71)	1.8 (−0.72, 5.21)	354 (4.21)	7.34 (6.59, 8.14)	1.83 (−0.6, 4.92)
NHW						
All	21 652 (58.98)	3.25 (3.21, 3.29)	1.02 (0.54, 1.49)	15 061 (41.02)	1.93 (1.9, 1.96)	1.62 (1.05, 2.12)
20–29	665 (1.81)	0.63 (0.58, 0.68)	−0.08 (−3.38, 4.73)	412 (1.12)	0.4 (0.36, 0.44)	1.37 (−0.31, 3.2)
30–39	1107 (3.02)	1.04 (0.98, 1.1)	1.09 (−0.13, 2.35)	738 (2.01)	0.7 (0.65, 0.75)	2.24 (0.82, 3.78)
40–49	2097 (5.71)	1.73 (1.66, 1.81)	0.8 (−0.32, 1.88)	1398 (3.81)	1.15 (1.09, 1.22)	1.99 (1.18, 2.81)
50–59	3875 (10.55)	3.21 (3.11, 3.31)	0.48 (−0.15, 1.14)	2659 (7.24)	2.15 (2.06, 2.23)	1.8 (0.39, 3.39)
60–69	5337 (14.54)	6.23 (6.06, 6.4)	0.59 (−0.15, 1.4)	3540 (9.64)	3.82 (3.7, 3.95)	1.03 (0.15, 2.06)
70–79	5202 (14.17)	10.16 (9.88, 10.44)	2.19 (0.69, 3.59)	3474 (9.46)	5.51 (5.33, 5.7)	1.93 (1.51, 2.39)
≥ 80	3369 (9.18)	12.04 (11.64, 12.46)	1.54 (−0.62, 4.74)	2840 (7.74)	5.89 (5.67, 6.11)	2.06 (1.09, 3.16)

Abbreviations: AAPC, average annual percent change; CI, confidence interval; NHB, non‐Hispanic Black; NHW, non‐Hispanic White.

^a^ The ASIR in the ‘All’ rows refers to the age‐standardized incidence rate. For individual age categories, the values represent age‐specific incidence rates.

There was an incline in MTNHL incident cases among men aged 20–24 to 65–69, after which it began to decline. In a similar fashion, women followed the same pattern. (Figure S29).

### 3.10 Non‐Hodgkin Lymphoma of Unknown Lineage

There were a total of 1485 reported cases of non‐Hodgkin lymphoma of unknown lineage (NHL‐U) in the US. Predominantly, these cases affected men (66.13%), NHW individuals (70.03%), and individuals aged 70–79 years (22.63%). The ASIR per 100 000 people was 0.10 (0.10, 0.11) for men and 0.04 (0.04, 0.05) for women (Table 10 and Figures S30–S32).

**TABLE 10:** Counts and age‐standardized rate of non‐Hodgkin lymphoma of unknown lineage incidence per 100 000 and average annual percent change from 2000 to 2019 in the United States, by age, sex, and race.

Age group (years)	Men			Women		
	Case (%)	ASIR^a^ (95% CI)	AAPC (95% CI)	Case (%)	ASIR (95% CI)	AAPC (95% CI)
Race/ethnicities						
All	982 (66.13)	0.1 (0.1, 0.11)	−5.87 (−7.49, −3.44)	503 (33.87)	0.04 (0.04, 0.05)	−8.78 (−10.15, −7.41)
20–29	95 (6.40)	0.04 (0.04, 0.05)	N/A	51 (3.43)	0.02 (0.02, 0.03)	N/A
30–39	84 (5.66)	0.04 (0.03, 0.05)	N/A	51 (3.43)	0.02 (0.02, 0.03)	N/A
40–49	100 (6.73)	0.05 (0.04, 0.06)	−9.55 (−17.94, −4.31)	39 (2.63)	0.02 (0.01, 0.03)	N/A
50–59	117 (7.88)	0.06 (0.05, 0.08)	−5.76 (−9.39, −0.13)	75 (5.05)	0.04 (0.03, 0.05)	N/A
60–69	178 (11.99)	0.15 (0.13, 0.17)	−4.65 (−8.02, 1.33)	85 (5.72)	0.06 (0.05, 0.08)	−8.77 (−14.45, −3.94)
70–79	237 (15.96)	0.34 (0.3, 0.39)	−2.4 (−4.8, 1.22)	99 (6.67)	0.11 (0.09, 0.14)	−7.43 (−10.71, −3.43)
≥ 80	171 (11.52)	0.48 (0.41, 0.55)	−3.03 (−6.17, 2.2)	103 (6.94)	0.17 (0.14, 0.2)	−5.21 (−10.19, −1.04)
Hispanic						
All	157 (67.09)	0.1 (0.08, 0.12)	−3.51 (−7.72, 2.87)	77 (32.91)	0.04 (0.03, 0.05)	N/A
20–29	32 (13.68)	0.05 (0.04, 0.07)	N/A	10 (4.27)	0.02 (0.01, 0.03)	N/A
30–39	24 (10.26)	0.04 (0.03, 0.06)	N/A	14 (5.98)	0.03 (0.01, 0.05)	N/A
40–49	19 (8.12)	0.04 (0.03, 0.07)	N/A	11 (4.70)	0.03 (0.01, 0.05)	N/A
50–59	21 (8.97)	0.07 (0.04, 0.11)	N/A	10 (4.27)	0.03 (0.02, 0.06)	N/A
60–69	23 (9.83)	0.15 (0.1, 0.23)	N/A	11 (4.70)	0.06 (0.03, 0.11)	N/A
70–79	28 (11.97)	0.38 (0.25, 0.55)	N/A	14 (5.98)	0.14 (0.08, 0.24)	N/A
≥ 80	10 (4.27)	0.31 (0.15, 0.57)	N/A	7 (2.99)	0.13 (0.05, 0.27)	N/A
NHB						
All	77 (54.23)	0.08 (0.06, 0.1)	N/A	65 (45.77)	0.05 (0.04, 0.07)	−7.2 (−12.98, −2.4)
20–29	13 (9.15)	0.05 (0.03, 0.08)	N/A	10 (7.04)	0.04 (0.02, 0.07)	N/A
30–39	5 (3.52)	0.02 (0.01, 0.05)	N/A	9 (6.34)	0.03 (0.02, 0.07)	N/A
40–49	18 (12.68)	0.08 (0.05, 0.12)	N/A	8 (5.63)	0.03 (0.01, 0.06)	N/A
50–59	14 (9.86)	0.07 (0.04, 0.12)	N/A	16 (11.27)	0.07 (0.04, 0.12)	N/A
60–69	12 (8.45)	0.11 (0.06, 0.19)	N/A	6 (4.23)	0.04 (0.01, 0.09)	N/A
70–79	10 (7.04)	0.18 (0.09, 0.33)	N/A	11 (7.75)	0.14 (0.07, 0.24)	N/A
≥ 80	5 (3.52)	0.23 (0.07, 0.53)	N/A	5 (3.52)	0.11 (0.03, 0.25)	N/A
NHW						
All	703 (67.60)	0.11 (0.1, 0.12)	−6.16 (−8.27, −2.72)	337 (32.40)	0.04 (0.04, 0.05)	−8.86 (−10.53, −7.14)
20–29	42 (4.04)	0.04 (0.03, 0.05)	N/A	26 (2.50)	0.03 (0.02, 0.04)	N/A
30–39	46 (4.42)	0.04 (0.03, 0.06)	N/A	24 (2.31)	0.02 (0.01, 0.03)	N/A
40–49	58 (5.58)	0.05 (0.04, 0.06)	N/A	18 (1.73)	0.02 (0.01, 0.02)	N/A
50–59	76 (7.31)	0.06 (0.05, 0.08)	N/A	43 (4.13)	0.03 (0.03, 0.05)	N/A
60–69	139 (13.37)	0.16 (0.14, 0.19)	−2.75 (−5.6, 1.77)	65 (6.25)	0.07 (0.05, 0.09)	N/A
70–79	191 (18.37)	0.37 (0.32, 0.43)	−2.98 (−5.71, 1.02)	73 (7.02)	0.12 (0.09, 0.15)	−7.69 (−10.8, −4.35)
≥ 80	151 (14.52)	0.54 (0.46, 0.63)	−3.38 (−6.53, 0.81)	88 (8.46)	0.18 (0.15, 0.22)	−4.48 (−9.37, −0.56)

Abbreviations: AAPC, average annual percent change; CI, confidence interval; N/A, not available; NHB, non‐Hispanic Black; NHW, non‐Hispanic White.

^a^ The ASIR in the ‘All’ rows refers to the age‐standardized incidence rate. For individual age categories, the values represent age‐specific incidence rates.

There was a substantial rise in incident cases between 50–54 and 70–74, the age group in which the NHL‐UL reached its peak in men. Women had a minimal increase beginning from the 60–64 age group, after which it remained almost constant (Figure S33).

## 4 Discussion

### 4.1 Main Findings

From 2000 to 2014, adult NHL incidence rates significantly increased, followed by a notable decline from 2014 to 2019. B‐cell NHL was the predominant subtype, accounting for 93.59% of cases, with mature B‐cell NHL being the most common. ASIRs (per 100 000 population) were consistently higher in men than women; men experienced rising rates from 2000 to 2014 and a decline thereafter, while women showed no significant ASIR changes from 2004 to 2019. NHL ASIRs increased with age across all groups, with the 20–29 age group showing the highest AAPCs.

Racially, NHW men had the highest ASIRs, whereas NHB women exhibited the greatest increase. NHL rates in NHWs rose until 2015 and declined from 2015 to 2019; NHBs showed trend variations but experienced an overall decline from 2011 to 2019. Hispanic rates remained relatively stable from 2004 to 2019. Following the COVID‐19 pandemic, ASIRs declined significantly across all races, ethnicities, and sexes. For subtypes, both B‐cell and T‐cell lymphoma incidence rates increased between 2000 and 2015, followed by a decline from 2015 to 2019.

### 4.2 Overall Patterns of Adult NHL

In recent years, adult NHL incidence in the US has shown fluctuations. Earlier studies reported approximately 77 200 new cases in 2020, accounting for 4.3% of all cancer diagnoses and ranking NHL as the seventh most common cancer [5]. In 2017, the incidence rate was 18.6 per 100 000, marking a 168% increase from 11.1 per 100 000 in 1975 [5]. Shiels et al., using SEER data (1992–2009), reported that NHL rates nearly doubled between 1974 and 2009, with a sharp increase in the early 1990s followed by stabilization. The increase continued for men until the early 1990s and for women until the early 2000s before plateauing [23]. These trends highlight a significant long‐term rise in NHL diagnoses. Our results showed a similar pattern, with incidence rates rising until 2014 and then declining significantly between 2014 and 2019.

Globally, comparable trends have been observed. Ho et al. [24], using the Global Burden of Disease (GBD) 2019 data, analyzed lymphoma and leukemia subtypes in Australia and Oceania, reporting an increase in NHL incidence. Similarly, Su et al. [25] found a rising trend in ASIRs of NHL in China from 1990 to 2019. These findings, consistent with our SEER‐based analysis in the US, reflect an increasing global burden of NHL. However, differences in population demographics, healthcare systems, and data sources between SEER and GBD registries should be considered when interpreting cross‐national trends.

### 4.3 Age and Sex Patterns

Previous research has shown that the overall age ASIR of NHL in the US is higher in men (23.9 per 100 000) than in women (16.4 per 100 000) [26], consistent with our findings. Globally, men have more than double the lifetime risk of developing NHL, and in countries with high human development index, are 39% more likely to be diagnosed and 60% more likely to die from the disease. In our study, ASIRs increased in both sexes, with an AAPC of 0.45% in men and 0.38% in women. This aligns with projections of increasing NHL incidence through 2040 [23] and with Cai et al., who reported rising rates from 1990 to 2019 with APCs of 0.78 in men and 0.25 in women [26].

NHL incidence increased with age in our analysis. Prior studies reported a mean diagnosis age of 65, varying by histological subtype [27]. Age‐related immune system changes [23] and increased t(11;14) translocations [28] may contribute to this trend.

Wasifuddin et al. [29] found a declining incidence of DLBCL in adolescents and young adults (AYAs) from 2000 to 2020 using the SEER data, while Zhang et al. [30] also observed declining NHL rates. Our study, which analyzed B‐cell NHL as a whole rather than by subtype, showed an increasing trend in adults from 2000 to 2019. Although we did not focus exclusively on AYAs, our joinpoint analysis included individuals aged 20–39. We found a significant increase in incidence among those aged 20–29 (APC = 0.37%, *p* < 0.05), while the 30–39 age group showed a non‐significant decline. These discrepancies may reflect methodological differences in age stratification and subtype classification. Notably, our findings suggest potential intra‐group variations within the AYA category. Further research focusing on B‐cell NHL subtypes and age‐stratified AYA analyses is warranted.

### 4.4 COVID‐19 Impacts on Adult NHL

The COVID‐19 pandemic in 2020 caused significant interruptions in healthcare services throughout the US [31]. This led to a reduction in face‐to‐face primary care consultations and cancer screenings [31]. Our study showed a significant decrease in NHL incidence rate during the COVID‐19 pandemic. A study by Mariotto et al. on the SEER database also indicated that NHL age‐standardized, delay‐adjusted incidence rates in men and women in 2019 and 2020 had decreased from 23.3 to 21.5 (PC: −7.7%) and 16.1 to 14.7 (PC: −8.4%), respectively [32]. There were similar decreasing patterns for myeloma, as well as kidney and renal pelvis cancers, following the COVID‐19 pandemic in the US [33, 34]. The drop in incidence was likely caused by less access to healthcare and fewer diagnoses during the pandemic. More research is needed to find out if the decrease was only due to delays in detection or if other factors, like changes in disease risk, how people used healthcare, or how cases were reported, also played a part.

### 4.5 Histological Subtypes

B‐cell lymphomas account for approximately 95% of all lymphomas [35], with an incidence of about 20 new cases per 100 000 individuals annually in high‐income countries [36]. Several factors may explain their higher prevalence compared to T‐cell lymphomas in the US, including better 5‐year relative survival rates [37] and genetic variants that may predispose B cells to malignant transformation [38].

DLBCL, classified under mature B‐cell neoplasms, was the most common aggressive lymphoma in 2016 [39, 40] and is consistently reported as the most prevalent B‐cell lymphoma subtype [13, 41]. Our findings align with other studies recommending that mature B‐cell NHL be identified as the most frequent subtype [39], likely due to its varied cellular origins, genetic heterogeneity, and distinct clinical behavior compared to other B‐cell neoplasms [42]. While Howlader et al. noted a decline in B‐cell NHL subtype incidence after 2003, peripheral T‐cell lymphoma rates remained stable from 2006 to 2011 [43].

### 4.6 Race/Ethnicity

In the US, NHW individuals face the highest likelihood of developing NHL, while those from Asian/Pacific Islander, American Indian, and Black communities have the lowest susceptibility [5]. Our findings confirmed that NHW men had the highest ASIR, while NHB women showed the most substantial increase in ASIRs over the study period. Environmental pollutants and lifestyle factors may contribute to the rising NHL incidence among African Americans. Exposure to pollutants such as polychlorinated biphenyls, dichlorodiphenyltrichloroethane, and pesticides has been linked to NHL development [30], and urban environments—more common among NHBs—are associated with higher incidence [44]. Blansky et al., using the SEER data, found that urban residence correlated with increased DLBCL incidence in Hispanics and chronic lymphocytic leukemia in NHBs. After adjusting for demographic and socioeconomic variables, DLBCL incidence remained higher in metropolitan areas, especially among Hispanics [44].

Our analysis showed that among Hispanics, adult NHL incidence rose from 2000 to 2004, then plateaued until 2009. Miller et al. reported NHL among the leading cancer sites for both incidence and mortality in US Hispanics, estimating approximately 4700 and 4200 new cases in Hispanic men and women, respectively, in 2021 [45]. Subtype distribution also differs: Portillo et al. found significantly higher rates of B‐cell NHL subtypes (DLBCL, follicular lymphoma, Burkitt lymphoma) and T‐cell subtypes (NK/T‐cell and anaplastic lymphoma) among Hispanics compared to non‐Hispanics [46]. Additionally, studies in Texas and Florida showed comparable outcomes in peripheral T‐cell lymphomas between Hispanics and non‐Hispanics, despite differences in demographics such as age and insurance coverage [47]. These findings highlight ethnic variation in NHL subtype distribution and demographic profiles, underscoring the need for further research to clarify these associations and temporal trends across racial and ethnic groups.

### 4.7 Limitations and Strengths

This study has several limitations. First, it did not account for major NHL risk factors, such as Epstein–Barr virus prevalence. Second, potential misclassification of cases or histological subtypes within the database may have introduced inaccuracies. Variability in healthcare access and diagnostic services across regions and demographics could have influenced incidence reporting. Additionally, changes in diagnostic criteria and improvements in detection over the two‐decade span may affect data consistency. While this study focused on incidence trends, future research should incorporate survival and treatment‐specific outcomes, especially in the context of emerging therapies like chimeric antigen receptor (CAR) T‐cell therapy. Finally, findings based on the SEER database may not be generalizable to other populations or healthcare systems, limiting external validity.

Despite these limitations, the study has notable strengths. The SEER database provides high‐quality, nationally representative data on adult NHL in the US. Covering the period from 2000 to 2020, it allows for a robust analysis of long‐term trends. The inclusion of data reflecting the impact of the COVID‐19 pandemic adds contemporary relevance. However, it is important to note that some of the observed declines in incidence during the pandemic period may not necessarily reflect true changes in cancer trends, but rather reduced detection due to limited healthcare access. Furthermore, the use of standardized and precise histologic criteria ensured accurate classification of NHL subtypes.

## 5 Conclusions

The overall incidence rate of adult NHL increased until 2014, then experienced a significant decline from 2014 to 2019. Adult NHL had a different trend based on race, sex, age group, and histological subtype. Overall, the ASIR of NHL in the US was higher in men. During COVID‐19 pandemic adult NHL indicated a significant decrease in incidence rates. Mature B‐cell lymphoma was the most common subtype, and NHW had the highest ASIR. There is a critical need for more research to understand why NHL rates decreased in the US between 2014 and 2019. Investigating the underlying causes of this decline could be beneficial for planning effective prevention strategies. Moreover, developing targeted prevention measures that address the various NHL risk factors could significantly reduce the global cancer burden and improve health outcomes worldwide.

## Author Contributions

S.E.M. and S.A.N. designed the study. S.E.M. and S.A.N. analyzed the data and performed the statistical analyses. S.E.M., M.N., H.M., A.A., Z.Y., and S.A.N. drafted the initial manuscript. S.E.M., Z.Y., and S.A.N. critically edited and revised the initial draft of the manuscript. All authors reviewed the drafted manuscript for critical content. All authors approved the final version of the manuscript.

## Ethics Statement

The authors have nothing to report.

## Conflicts of Interest

The authors declare no conflicts of interest.

## Supporting information


**Appendix S1.** Supporting Information.

## Data Availability

The data that support the findings of this study are openly available in Surveillance, Epidemiology, and End Results Program at https://seer.cancer.gov/data‐software/.
