# Supplementary material for: Trends of Non‐Hodgkin Lymphoma Incidence Among Adults in the United States From 2000 to 2020
Source: Cancer Rep (Hoboken). 2025 Jun 30;8(7):e70269. doi: 10.1002/cnr2.70269 (PMC12207246; doi:10.1002/cnr2.70269)
Supplement: Supplementary file 1 — Appendix S1. Supporting Information. [file CNR2-8-e70269-s001.docx]

**Table S1.** Counts and age-standardized rate of non-Hodgkin lymphoma incidence per 100,000 and average annual percent change from 2015 to 2019 in the United States, by age, sex, and race.

| **Race/ethnicities** | | | | | | |
| --- | --- | --- | --- | --- | --- | --- |
| **Age group (years)** | **Men** | | | **Women** | | |
|  | **Case (%)** | **ASIR (95% CI)** | **AAPC (95% CI)** | **Case (%)** | **ASIR (95% CI)** | **AAPC (95% CI)** |
| **All** | 158338 (56.22) | 55.87 (55.59, 56.16) | -0.94 (-1.61, -0.51) | 123303(43.78) | 36.3 (36.1, 36.51) | -0.6 (-1.36, -0.01) |
| **20 to 29** | 2739 (0.97) | 4.79 (4.62, 4.98) | 0.2 (-1.27, 0.68) | 1776 (0.63) | 3.25 (3.1, 3.4) | 0.73 (0.17, 1.34) |
| **30 to 39** | 4741 (1.68) | 9 (8.75, 9.26) | -0.24 (-0.57, 0.1) | 3432 (1.22) | 6.58 (6.36, 6.8) | 0.68 (0.3, 1.08) |
| **40 to 49** | 10195 (3.62) | 20.48 (20.08, 20.88) | -0.94 (-1.59, -0.62) | 7646 (2.71) | 15.07 (14.73, 15.41) | 0.63 (0.38, 0.87) |
| **50 to 59** | 26378 (9.37) | 51.63 (51, 52.26) | 0.11 (-0.06, 0.29) | 19006 (6.75) | 35.77 (35.26, 36.29) | 0.25 (0.04, 0.47) |
| **60 to 69** | 43982 (15.62) | 113.01 (111.95, 114.07) | -1 (-1.86, -0.43) | 32489 (11.54) | 75.12 (74.3, 75.94) | -0.31 (-0.67, -0.08) |
| **70 to 79** | 42660 (15.15) | 203.88 (201.93, 205.84) | -1.01 (-1.68, -0.51) | 33129 (11.76) | 129.52 (128.12, 130.93) | -0.05 (-0.38, 0.17) |
| **≥ 80** | 27643 (9.81) | 261.45 (258.38, 264.56) | -1.36 (-2.15, -0.75) | 25825 (9.17) | 151.96 (150.09, 153.85) | -0.52 (-0.91, -0.22) |
| **Hispanic** | | | | | | |
| **Age groups** | **Men** | | | **Women** | | |
|  | **Case (%)** | **ASIR (95% CI)** | **AAPC (95% CI)** | **Case (%)** | **ASIR (95% CI)** | **AAPC (95% CI)** |
| **All** | 20275 (53.65) | 45.56 (44.88, 46.24) | 0.18 (-0.11, 0.53) | 17518 (46.35) | 33.49 (32.98, 34) | 0.18 (-0.98, 0.48) |
| **20 to 29** | 887 (2.35) | 5.26 (4.92, 5.62) | 1.72 (0.86, 2.67) | 559 (1.48) | 3.55 (3.26, 3.86) | 1.17 (0.19, 2.27) |
| **30 to 39** | 1299 (3.44) | 8.56 (8.1, 9.04) | 0.99 (0.28, 2.7) | 888 (2.35) | 6.3 (5.89, 6.73) | 1.01 (0.25, 1.87) |
| **40 to 49** | 2329 (6.16) | 17.91 (17.19, 18.65) | -0.62 (-0.98, -0.25) | 1783 (4.72) | 13.93 (13.29, 14.59) | 0.72 (0.08, 1.48) |
| **50 to 59** | 4182 (11.07) | 42.41 (41.13, 43.71) | 0.21 (-0.06, 0.53) | 3383 (8.95) | 33.78 (32.65, 34.94) | 0.56 (0.24, 0.93) |
| **60 to 69** | 5039 (13.33) | 90.44 (87.95, 92.99) | 0.31 (-0.04, 0.76) | 4468 (11.82) | 71.05 (68.97, 73.17) | 0.41 (0.05, 0.84) |
| **70 to 79** | 4057 (10.73) | 161.26 (156.29, 166.36) | 0.23 (-0.25, 0.82) | 3874 (10.25) | 118.09 (114.38, 121.89) | 0.3 (-0.09, 0.76) |
| **≥ 80** | 2482 (6.57) | 208.21 (200.1, 216.57) | 0.14 (-0.45, 0.92) | 2563 (6.78) | 132.89 (127.78, 138.15) | -0.4 (-5.21, 1.22) |
| **NHB** | | | | | | |
| **Age groups** | **Men** | | | **Women** | | |
|  | **Case (%)** | **ASIR (95% CI)** | **AAPC (95% CI)** | **Case (%)** | **ASIR (95% CI)** | **AAPC (95% CI)** |
| **All** | 15171 (51.52) | 56.25 (55.3, 57.21) | -0.8 (-1.44, -0.33) | 14278 (48.48) | 39.79 (39.13, 40.47) | 0.86 (0.49, 1.29) |
| **20 to 29** | 345 (1.17) | 4.48 (4.02, 4.98) | -2.99 (-12.25, -0.14) | 241 (0.82) | 3.18 (2.79, 3.6) | -0.21 (-1.49, 1.13) |
| **30 to 39** | 698 (2.37) | 11.4 (10.57, 12.28) | -0.07 (-1.06, 0.89) | 565 (1.92) | 8.6 (7.91, 9.34) | 0.37 (-0.55, 1.32) |
| **40 to 49** | 1467 (4.98) | 26.06 (24.74, 27.44) | -0.24 (-0.82, 0.34) | 1344 (4.56) | 21.22 (20.1, 22.39) | 1.21 (0.61, 1.84) |
| **50 to 59** | 3279 (11.13) | 57.95 (55.97, 59.98) | -0.44 (-4.03, 0.85) | 2756 (9.36) | 42.65 (41.06, 44.28) | 0.64 (0.16, 1.18) |
| **60 to 69** | 4565 (15.50) | 122.05 (118.52, 125.66) | 0.38 (-0.4, 0.82) | 4009 (13.61) | 84.36 (81.76, 87.01) | -0.71 (-2.89, 0.4) |
| **70 to 79** | 3136 (10.65) | 183.7 (177.26, 190.31) | -2.52 (-5.51, -0.63) | 3340 (11.34) | 133.31 (128.81, 137.93) | -1.48 (-5.45, 1.88) |
| **≥ 80** | 1681 (5.71) | 228.72 (217.9, 239.94) | 1.08 (0.63, 1.6) | 2023 (6.87) | 139.44 (133.41, 145.68) | 0.42 (-0.15, 1.08) |
| **NHW** | | | | | | |
| **Age groups** | **Men** | | | **Women** | | |
|  | **Case (%)** | **ASIR (95% CI)** | **AAPC (95% CI)** | **Case (%)** | **ASIR (95% CI)** | **AAPC (95% CI)** |
| **All** | 112757 (57.47) | 59.66 (59.3, 60.02) | -1.21 (-1.92, -0.53) | 83456 (42.53) | 37.52 (37.25, 37.78) | -0.06 (-0.23, 0.08) |
| **20 to 29** | 1214 (0.62) | 4.52 (4.27, 4.78) | 0.16 (-0.43, 0.77) | 751 (0.38) | 2.93 (2.73, 3.15) | 0.22 (-0.63, 1.1) |
| **30 to 39** | 2260 (1.15) | 8.83 (8.47, 9.21) | -0.48 (-0.87, -0.1) | 1566 (0.80) | 6.23 (5.93, 6.55) | 0.39 (0.03, 0.75) |
| **40 to 49** | 5560 (2.83) | 21.43 (20.87, 22) | -0.87 (-1.44, -0.54) | 3867 (1.97) | 15.02 (14.55, 15.5) | 0.6 (0.31, 0.89) |
| **50 to 59** | 17144 (8.74) | 55.09 (54.26, 55.93) | -0.98 (-2.28, 0.32) | 11495 (5.86) | 36.32 (35.65, 37) | 0.14 (-0.05, 0.32) |
| **60 to 69** | 31534 (16.07) | 119.79 (118.47, 121.12) | -1.16 (-1.94, -0.42) | 21925 (11.17) | 77.65 (76.63, 78.69) | -0.22 (-0.64, -0.03) |
| **70 to 79** | 33024 (16.83) | 220.79 (218.4, 223.2) | -1.11 (-1.74, -0.3) | 23932 (12.20) | 135.89 (134.17, 137.63) | -0.08 (-0.3, 0.11) |
| **≥ 80** | 22021 (11.22) | 283.67 (279.93, 287.44) | -1.3 (-2.28, -0.69) | 19920 (10.15) | 162.35 (160.06, 164.66) | -0.33 (-0.74, -0.03) |

**Abbreviations:** NHW: Non-Hispanic White; NHB: Non-Hispanic Black; ASIR: Age-standardized incidence rate; CI: Confidence interval, AAPC: Average annual percent change

**Table S2**. Results of the tests of incidental trends for non-Hodgkin lymphoma incidence rate among all ages over 2000-2019 in the United States

| **Cohort 1** | | | **Cohort 2** | | | **p-value** |
| --- | --- | --- | --- | --- | --- | --- |
| **sex** | **Subtype** | **race** | **sex** | **Subtype** | **race** |  |
| Male | B-cell NHL NOS | Hispanic | Male | B-cell NHL NOS | NHB | 0.07 |
| Female | NHL unknown lineage | All | Female | NHL unknown lineage | NHB | 0.29 |
| Female | NHL unknown lineage | All | Female | NHL unknown lineage | NHW | 0.56 |
| Male | NHL unknown lineage | All | Male | NHL unknown lineage | Hispanic | 0.32 |
| Male | NHL unknown lineage | All | Male | NHL unknown lineage | NHW | 0.16 |
| Male | NHL unknown lineage | Hispanic | Male | NHL unknown lineage | NHW | 0.65 |
| Both | NHL unknown lineage | All | Both | NHL unknown lineage | Hispanic | 0.06 |
| Both | NHL unknown lineage | All | Both | NHL unknown lineage | NHB | 0.08 |
| Both | NHL unknown lineage | All | Both | NHL unknown lineage | NHW | 0.34 |
| Both | NHL unknown lineage | Hispanic | Both | NHL unknown lineage | NHW | 0.09 |
| Both | NHL unknown lineage | NHB | Both | NHL unknown lineage | NHW | 0.3 |
| Female | Precursor T-cell NHL | All | Female | Precursor T-cell NHL | Hispanic | 0.26 |
| Female | Precursor T-cell NHL | All | Female | Precursor T-cell NHL | NHW | 0.73 |
| Female | Precursor T-cell NHL | Hispanic | Female | Precursor T-cell NHL | NHW | 0.22 |
| Both | Precursor T-cell NHL | All | Both | Precursor T-cell NHL | Hispanic | 0.1 |
| Both | Precursor T-cell NHL | All | Both | Precursor T-cell NHL | NHW | 0.23 |
| Both | Precursor T-cell NHL | Hispanic | Both | Precursor T-cell NHL | NHW | 0.07 |
| Male | B-cell NHL NOS | NHB | Male | B-cell NHL NOS | Hispanic | 0.07 |
| Both | Precursor T-cell NHL | NHW | Both | Precursor T-cell NHL | Hispanic | 0.07 |
| Both | Precursor T-cell NHL | NHW | Both | Precursor T-cell NHL | All | 0.24 |
| Both | Precursor T-cell NHL | Hispanic | Both | Precursor T-cell NHL | All | 0.11 |
| Female | Precursor T-cell NHL | NHW | Female | Precursor T-cell NHL | Hispanic | 0.22 |
| Female | Precursor T-cell NHL | NHW | Female | Precursor T-cell NHL | All | 0.74 |
| Female | Precursor T-cell NHL | Hispanic | Female | Precursor T-cell NHL | All | 0.26 |
| Both | NHL unknown lineage | NHW | Both | NHL unknown lineage | NHB | 0.29 |
| Both | NHL unknown lineage | NHW | Both | NHL unknown lineage | Hispanic | 0.09 |
| Both | NHL unknown lineage | NHW | Both | NHL unknown lineage | All | 0.35 |
| Both | NHL unknown lineage | NHB | Both | NHL unknown lineage | All | 0.08 |
| Both | NHL unknown lineage | Hispanic | Both | NHL unknown lineage | All | 0.05 |
| Male | NHL unknown lineage | NHW | Male | NHL unknown lineage | Hispanic | 0.65 |
| Male | NHL unknown lineage | NHW | Male | NHL unknown lineage | All | 0.16 |
| Male | NHL unknown lineage | Hispanic | Male | NHL unknown lineage | All | 0.32 |
| Female | NHL unknown lineage | NHW | Female | NHL unknown lineage | All | 0.55 |
| Female | NHL unknown lineage | NHB | Female | NHL unknown lineage | All | 0.29 |
| Female | NHL unknown lineage | All | Female | NHL unknown lineage | NHB | 0.29 |
| Female | NHL unknown lineage | All | Female | NHL unknown lineage | NHW | 0.54 |
| Female | Precursor T-cell NHL | All | Female | Precursor T-cell NHL | Hispanic | 0.26 |
| Female | Precursor T-cell NHL | All | Female | Precursor T-cell NHL | NHW | 0.74 |
| Female | Precursor T-cell NHL | Hispanic | Female | Precursor T-cell NHL | NHW | 0.21 |
| Male | B-cell NHL NOS | Hispanic | Male | B-cell NHL NOS | NHB | 0.07 |
| Male | NHL unknown lineage | All | Male | NHL unknown lineage | Hispanic | 0.33 |
| Male | NHL unknown lineage | All | Male | NHL unknown lineage | NHW | 0.15 |
| Male | NHL unknown lineage | Hispanic | Male | NHL unknown lineage | NHW | 0.65 |
| Both | NHL unknown lineage | All | Both | NHL unknown lineage | Hispanic | 0.05 |
| Both | NHL unknown lineage | All | Both | NHL unknown lineage | NHB | 0.08 |
| Both | NHL unknown lineage | All | Both | NHL unknown lineage | NHW | 0.35 |
| Both | NHL unknown lineage | Hispanic | Both | NHL unknown lineage | NHW | 0.09 |
| Both | NHL unknown lineage | NHB | Both | NHL unknown lineage | NHW | 0.3 |
| Both | Precursor T-cell NHL | All | Both | Precursor T-cell NHL | Hispanic | 0.11 |
| Both | Precursor T-cell NHL | All | Both | Precursor T-cell NHL | NHW | 0.23 |
| Both | Precursor T-cell NHL | Hispanic | Both | Precursor T-cell NHL | NHW | 0.08 |

**Abbreviations**: **NHW:** Non-Hispanic White; **NHB:** Non-Hispanic Black; **NHL**; Non-Hodgkin lymphoma, **NOS**: Not Otherwise Specified

**Table S3**. Results of the tests of parallel trends for non-Hodgkin lymphoma incidence rate among all ages over 2000-2019 in the United States

| **Cohort 1** | | | **Cohort 2** | | | **p-value** |
| --- | --- | --- | --- | --- | --- | --- |
| **sex** | **type** | **race** | **sex** | **type** | **race** |  |
| Female | Mature B-cell NHL | All | Female | Mature B-cell NHL | NHW | 0.28 |
| Female | Mature B-cell NHL | Hispanic | Female | Mature B-cell NHL | NHB | 0.1 |
| Female | Mature B-cell NHL | Hispanic | Female | Mature B-cell NHL | NHW | 0.05 |
| Female | Mature T-cell NHL | All | Female | Mature T-cell NHL | Hispanic | 0.35 |
| Female | Mature T-cell NHL | All | Female | Mature T-cell NHL | NHW | 0.85 |
| Female | Mature T-cell NHL | Hispanic | Female | Mature T-cell NHL | NHW | 0.3 |
| Female | NHL | All | Female | NHL | NHW | 0.4 |
| Female | NHL | Hispanic | Female | NHL | NHW | 0.13 |
| Female | B-cell NHL | All | Female | B-cell NHL | NHW | 0.71 |
| Female | B-cell NHL | Hispanic | Female | B-cell NHL | NHB | 0.13 |
| Female | B-cell NHL NOS | All | Female | B-cell NHL NOS | Hispanic | 0.36 |
| Female | B-cell NHL NOS | All | Female | B-cell NHL NOS | NHB | 0.24 |
| Female | B-cell NHL NOS | All | Female | B-cell NHL NOS | NHW | 0.69 |
| Female | B-cell NHL NOS | Hispanic | Female | B-cell NHL NOS | NHB | 0.87 |
| Female | B-cell NHL NOS | NHB | Female | B-cell NHL NOS | NHW | 0.09 |
| Female | T-cell NHL | All | Female | T-cell NHL | Hispanic | 0.05 |
| Female | T-cell NHL | All | Female | T-cell NHL | NHB | 0.06 |
| Female | T-cell NHL | All | Female | T-cell NHL | NHW | 0.92 |
| Female | T-cell NHL | Hispanic | Female | T-cell NHL | NHW | 0.2 |
| Female | NHL unknown lineage | All | Female | NHL unknown lineage | NHB | 0.75 |
| Female | NHL unknown lineage | All | Female | NHL unknown lineage | NHW | 0.34 |
| Female | NHL unknown lineage | NHB | Female | NHL unknown lineage | NHW | 0.81 |
| Female | Precursor B-cell NHL | All | Female | Precursor B-cell NHL | Hispanic | 0.65 |
| Female | Precursor B-cell NHL | All | Female | Precursor B-cell NHL | NHB | 0.19 |
| Female | Precursor B-cell NHL | Hispanic | Female | Precursor B-cell NHL | NHB | 0.36 |
| Female | Precursor B-cell NHL | Hispanic | Female | Precursor B-cell NHL | NHW | 0.24 |
| Female | Precursor B-cell NHL | NHB | Female | Precursor B-cell NHL | NHW | 0.83 |
| Female | Precursor T-cell NHL | All | Female | Precursor T-cell NHL | Hispanic | 0.34 |
| Female | Precursor T-cell NHL | All | Female | Precursor T-cell NHL | NHW | 0.72 |
| Female | Precursor T-cell NHL | Hispanic | Female | Precursor T-cell NHL | NHW | 0.31 |
| Male | Mature B-cell NHL | All | Male | Mature B-cell NHL | Hispanic | 0.12 |
| Male | Mature B-cell NHL | Hispanic | Male | Mature B-cell NHL | NHB | 0.11 |
| Male | Mature B-cell NHL | Hispanic | Male | Mature B-cell NHL | NHW | 0.06 |
| Male | Mature T-cell NHL | All | Male | Mature T-cell NHL | Hispanic | 0.75 |
| Male | Mature T-cell NHL | All | Male | Mature T-cell NHL | NHW | 0.28 |
| Male | Mature T-cell NHL | Hispanic | Male | Mature T-cell NHL | NHB | 0.46 |
| Male | Mature T-cell NHL | Hispanic | Male | Mature T-cell NHL | NHW | 0.68 |
| Male | Mature T-cell NHL | NHB | Male | Mature T-cell NHL | NHW | 0.07 |
| Male | NHL | All | Male | NHL | Hispanic | 0.32 |
| Male | NHL | All | Male | NHL | NHW | 0.05 |
| Male | NHL | Hispanic | Male | NHL | NHB | 0.12 |
| Male | NHL | Hispanic | Male | NHL | NHW | 0.21 |
| Male | B-cell NHL | All | Male | B-cell NHL | Hispanic | 0.21 |
| Male | B-cell NHL | Hispanic | Male | B-cell NHL | NHB | 0.1 |
| Male | B-cell NHL | Hispanic | Male | B-cell NHL | NHW | 0.08 |
| Male | B-cell NHL NOS | All | Male | B-cell NHL NOS | Hispanic | 0.98 |
| Male | B-cell NHL NOS | All | Male | B-cell NHL NOS | NHB | 0.58 |
| Male | B-cell NHL NOS | All | Male | B-cell NHL NOS | NHW | 0.28 |
| Male | B-cell NHL NOS | Hispanic | Male | B-cell NHL NOS | NHB | 0.37 |
| Male | B-cell NHL NOS | Hispanic | Male | B-cell NHL NOS | NHW | 0.89 |
| Male | B-cell NHL NOS | NHB | Male | B-cell NHL NOS | NHW | 0.21 |
| Male | T-cell NHL | All | Male | T-cell NHL | Hispanic | 0.75 |
| Male | T-cell NHL | All | Male | T-cell NHL | NHW | 0.06 |
| Male | T-cell NHL | Hispanic | Male | T-cell NHL | NHB | 0.43 |
| Male | T-cell NHL | Hispanic | Male | T-cell NHL | NHW | 0.64 |
| Male | T-cell NHL | NHB | Male | T-cell NHL | NHW | 0.06 |
| Male | NHL unknown lineage | All | Male | NHL unknown lineage | Hispanic | 0.26 |
| Male | NHL unknown lineage | All | Male | NHL unknown lineage | NHW | 0.43 |
| Male | NHL unknown lineage | Hispanic | Male | NHL unknown lineage | NHW | 0.43 |
| Male | Precursor B-cell NHL | All | Male | Precursor B-cell NHL | Hispanic | 0.08 |
| Male | Precursor B-cell NHL | NHB | Male | Precursor B-cell NHL | NHW | 0.06 |
| Male | Precursor T-cell NHL | All | Male | Precursor T-cell NHL | Hispanic | 0.6 |
| Male | Precursor T-cell NHL | All | Male | Precursor T-cell NHL | NHB | 0.62 |
| Male | Precursor T-cell NHL | Hispanic | Male | Precursor T-cell NHL | NHB | 0.18 |
| Male | Precursor T-cell NHL | Hispanic | Male | Precursor T-cell NHL | NHW | 0.52 |
| Male | Precursor T-cell NHL | NHB | Male | Precursor T-cell NHL | NHW | 0.18 |
| Both | Mature B-cell NHL | All | Both | Mature B-cell NHL | Hispanic | 0.35 |
| Both | Mature B-cell NHL | Hispanic | Both | Mature B-cell NHL | NHW | 0.47 |
| Both | Mature T-cell NHL | All | Both | Mature T-cell NHL | Hispanic | 0.57 |
| Both | Mature T-cell NHL | All | Both | Mature T-cell NHL | NHW | 0.57 |
| Both | Mature T-cell NHL | Hispanic | Both | Mature T-cell NHL | NHW | 0.18 |
| Both | NHL | All | Both | NHL | Hispanic | 0.68 |
| Both | NHL | All | Both | NHL | NHW | 0.22 |
| Both | NHL | Hispanic | Both | NHL | NHW | 0.39 |
| Both | B-cell NHL | All | Both | B-cell NHL | Hispanic | 0.39 |
| Both | B-cell NHL | Hispanic | Both | B-cell NHL | NHW | 0.36 |
| Both | B-cell NHL NOS | All | Both | B-cell NHL NOS | Hispanic | 0.88 |
| Both | B-cell NHL NOS | All | Both | B-cell NHL NOS | NHB | 0.7 |
| Both | B-cell NHL NOS | All | Both | B-cell NHL NOS | NHW | 0.38 |
| Both | B-cell NHL NOS | Hispanic | Both | B-cell NHL NOS | NHB | 0.85 |
| Both | B-cell NHL NOS | Hispanic | Both | B-cell NHL NOS | NHW | 0.63 |
| Both | B-cell NHL NOS | NHB | Both | B-cell NHL NOS | NHW | 0.48 |
| Both | T-cell NHL | All | Both | T-cell NHL | Hispanic | 0.42 |
| Both | T-cell NHL | All | Both | T-cell NHL | NHW | 0.44 |
| Both | T-cell NHL | Hispanic | Both | T-cell NHL | NHW | 0.17 |
| Both | NHL unknown lineage | All | Both | NHL unknown lineage | Hispanic | 0.05 |
| Both | NHL unknown lineage | All | Both | NHL unknown lineage | NHW | 0.46 |
| Both | NHL unknown lineage | Hispanic | Both | NHL unknown lineage | NHW | 0.06 |
| Both | NHL unknown lineage | NHB | Both | NHL unknown lineage | NHW | 0.14 |
| Both | Precursor B-cell NHL | All | Both | Precursor B-cell NHL | Hispanic | 0.24 |
| Both | Precursor B-cell NHL | NHB | Both | Precursor B-cell NHL | NHW | 0.42 |
| Both | Precursor T-cell NHL | All | Both | Precursor T-cell NHL | Hispanic | 0.72 |
| Both | Precursor T-cell NHL | All | Both | Precursor T-cell NHL | NHB | 0.22 |
| Both | Precursor T-cell NHL | All | Both | Precursor T-cell NHL | NHW | 0.07 |
| Both | Precursor T-cell NHL | Hispanic | Both | Precursor T-cell NHL | NHW | 0.64 |
| Both | Precursor T-cell NHL | NHB | Both | Precursor T-cell NHL | NHW | 0.06 |
| Both | NHL | NHW | Both | NHL | Hispanic | 0.39 |
| Both | NHL | NHW | Both | NHL | All | 0.23 |
| Both | NHL | Hispanic | Both | NHL | All | 0.68 |
| Male | NHL | NHW | Male | NHL | Hispanic | 0.21 |
| Male | NHL | NHW | Male | NHL | All | 0.06 |
| Male | NHL | NHB | Male | NHL | Hispanic | 0.13 |
| Male | NHL | Hispanic | Male | NHL | All | 0.31 |
| Female | NHL | NHW | Female | NHL | Hispanic | 0.12 |
| Female | NHL | NHW | Female | NHL | All | 0.39 |
| Both | B-cell NHL | NHW | Both | B-cell NHL | Hispanic | 0.36 |
| Both | B-cell NHL | Hispanic | Both | B-cell NHL | All | 0.4 |
| Male | B-cell NHL | NHW | Male | B-cell NHL | Hispanic | 0.08 |
| Male | B-cell NHL | NHB | Male | B-cell NHL | Hispanic | 0.11 |
| Male | B-cell NHL | Hispanic | Male | B-cell NHL | All | 0.21 |
| Female | B-cell NHL | NHW | Female | B-cell NHL | All | 0.72 |
| Female | B-cell NHL | NHB | Female | B-cell NHL | Hispanic | 0.13 |
| Both | Precursor B-cell NHL | NHW | Both | Precursor B-cell NHL | NHB | 0.42 |
| Both | Precursor B-cell NHL | Hispanic | Both | Precursor B-cell NHL | All | 0.24 |
| Male | Precursor B-cell NHL | NHW | Male | Precursor B-cell NHL | NHB | 0.05 |
| Male | Precursor B-cell NHL | Hispanic | Male | Precursor B-cell NHL | All | 0.08 |
| Female | Precursor B-cell NHL | NHW | Female | Precursor B-cell NHL | NHB | 0.84 |
| Female | Precursor B-cell NHL | NHW | Female | Precursor B-cell NHL | Hispanic | 0.25 |
| Female | Precursor B-cell NHL | NHB | Female | Precursor B-cell NHL | Hispanic | 0.35 |
| Female | Precursor B-cell NHL | NHB | Female | Precursor B-cell NHL | All | 0.19 |
| Female | Precursor B-cell NHL | Hispanic | Female | Precursor B-cell NHL | All | 0.64 |
| Both | Mature B-cell NHL | NHW | Both | Mature B-cell NHL | Hispanic | 0.47 |
| Both | Mature B-cell NHL | Hispanic | Both | Mature B-cell NHL | All | 0.36 |
| Male | Mature B-cell NHL | NHW | Male | Mature B-cell NHL | Hispanic | 0.07 |
| Male | Mature B-cell NHL | NHB | Male | Mature B-cell NHL | Hispanic | 0.11 |
| Male | Mature B-cell NHL | Hispanic | Male | Mature B-cell NHL | All | 0.13 |
| Female | Mature B-cell NHL | NHW | Female | Mature B-cell NHL | Hispanic | 0.06 |
| Female | Mature B-cell NHL | NHW | Female | Mature B-cell NHL | All | 0.29 |
| Female | Mature B-cell NHL | NHB | Female | Mature B-cell NHL | Hispanic | 0.11 |
| Both | B-cell NHL NOS | NHW | Both | B-cell NHL NOS | NHB | 0.48 |
| Both | B-cell NHL NOS | NHW | Both | B-cell NHL NOS | Hispanic | 0.62 |
| Both | B-cell NHL NOS | NHW | Both | B-cell NHL NOS | All | 0.38 |
| Both | B-cell NHL NOS | NHB | Both | B-cell NHL NOS | Hispanic | 0.86 |
| Both | B-cell NHL NOS | NHB | Both | B-cell NHL NOS | All | 0.71 |
| Both | B-cell NHL NOS | Hispanic | Both | B-cell NHL NOS | All | 0.88 |
| Male | B-cell NHL NOS | NHW | Male | B-cell NHL NOS | NHB | 0.21 |
| Male | B-cell NHL NOS | NHW | Male | B-cell NHL NOS | Hispanic | 0.88 |
| Male | B-cell NHL NOS | NHW | Male | B-cell NHL NOS | All | 0.28 |
| Male | B-cell NHL NOS | NHB | Male | B-cell NHL NOS | Hispanic | 0.36 |
| Male | B-cell NHL NOS | NHB | Male | B-cell NHL NOS | All | 0.58 |
| Male | B-cell NHL NOS | Hispanic | Male | B-cell NHL NOS | All | 0.98 |
| Female | B-cell NHL NOS | NHW | Female | B-cell NHL NOS | NHB | 0.1 |
| Female | B-cell NHL NOS | NHW | Female | B-cell NHL NOS | All | 0.68 |
| Female | B-cell NHL NOS | NHB | Female | B-cell NHL NOS | Hispanic | 0.86 |
| Female | B-cell NHL NOS | NHB | Female | B-cell NHL NOS | All | 0.26 |
| Female | B-cell NHL NOS | Hispanic | Female | B-cell NHL NOS | All | 0.38 |
| Both | T-cell NHL | NHW | Both | T-cell NHL | Hispanic | 0.15 |
| Both | T-cell NHL | NHW | Both | T-cell NHL | All | 0.45 |
| Both | T-cell NHL | Hispanic | Both | T-cell NHL | All | 0.42 |
| Male | T-cell NHL | NHW | Male | T-cell NHL | NHB | 0.06 |
| Male | T-cell NHL | NHW | Male | T-cell NHL | Hispanic | 0.65 |
| Male | T-cell NHL | NHW | Male | T-cell NHL | All | 0.06 |
| Male | T-cell NHL | NHB | Male | T-cell NHL | Hispanic | 0.43 |
| Male | T-cell NHL | Hispanic | Male | T-cell NHL | All | 0.76 |
| Female | T-cell NHL | NHW | Female | T-cell NHL | Hispanic | 0.2 |
| Female | T-cell NHL | NHW | Female | T-cell NHL | All | 0.92 |
| Female | T-cell NHL | NHB | Female | T-cell NHL | All | 0.06 |
| Female | T-cell NHL | Hispanic | Female | T-cell NHL | All | 0.06 |
| Both | Precursor T-cell NHL | NHW | Both | Precursor T-cell NHL | NHB | 0.06 |
| Both | Precursor T-cell NHL | NHW | Both | Precursor T-cell NHL | Hispanic | 0.64 |
| Both | Precursor T-cell NHL | NHW | Both | Precursor T-cell NHL | All | 0.07 |
| Both | Precursor T-cell NHL | NHB | Both | Precursor T-cell NHL | All | 0.21 |
| Both | Precursor T-cell NHL | Hispanic | Both | Precursor T-cell NHL | All | 0.72 |
| Male | Precursor T-cell NHL | NHW | Male | Precursor T-cell NHL | NHB | 0.18 |
| Male | Precursor T-cell NHL | NHW | Male | Precursor T-cell NHL | Hispanic | 0.53 |
| Male | Precursor T-cell NHL | NHB | Male | Precursor T-cell NHL | Hispanic | 0.18 |
| Male | Precursor T-cell NHL | NHB | Male | Precursor T-cell NHL | All | 0.63 |
| Male | Precursor T-cell NHL | Hispanic | Male | Precursor T-cell NHL | All | 0.62 |
| Female | Precursor T-cell NHL | NHW | Female | Precursor T-cell NHL | Hispanic | 0.33 |
| Female | Precursor T-cell NHL | NHW | Female | Precursor T-cell NHL | All | 0.72 |
| Female | Precursor T-cell NHL | Hispanic | Female | Precursor T-cell NHL | All | 0.34 |
| Both | Mature T-cell NHL | NHW | Both | Mature T-cell NHL | Hispanic | 0.17 |
| Both | Mature T-cell NHL | NHW | Both | Mature T-cell NHL | All | 0.58 |
| Both | Mature T-cell NHL | Hispanic | Both | Mature T-cell NHL | All | 0.56 |
| Male | Mature T-cell NHL | NHW | Male | Mature T-cell NHL | NHB | 0.08 |
| Male | Mature T-cell NHL | NHW | Male | Mature T-cell NHL | Hispanic | 0.67 |
| Male | Mature T-cell NHL | NHW | Male | Mature T-cell NHL | All | 0.28 |
| Male | Mature T-cell NHL | NHB | Male | Mature T-cell NHL | Hispanic | 0.46 |
| Male | Mature T-cell NHL | Hispanic | Male | Mature T-cell NHL | All | 0.75 |
| Female | Mature T-cell NHL | NHW | Female | Mature T-cell NHL | Hispanic | 0.29 |
| Female | Mature T-cell NHL | NHW | Female | Mature T-cell NHL | All | 0.84 |
| Female | Mature T-cell NHL | Hispanic | Female | Mature T-cell NHL | All | 0.36 |
| Both | NHL unknown lineage | NHW | Both | NHL unknown lineage | NHB | 0.14 |
| Both | NHL unknown lineage | NHW | Both | NHL unknown lineage | Hispanic | 0.06 |
| Both | NHL unknown lineage | NHW | Both | NHL unknown lineage | All | 0.46 |
| Both | NHL unknown lineage | Hispanic | Both | NHL unknown lineage | All | 0.05 |
| Male | NHL unknown lineage | NHW | Male | NHL unknown lineage | Hispanic | 0.41 |
| Male | NHL unknown lineage | NHW | Male | NHL unknown lineage | All | 0.42 |
| Male | NHL unknown lineage | Hispanic | Male | NHL unknown lineage | All | 0.26 |
| Female | NHL unknown lineage | NHW | Female | NHL unknown lineage | NHB | 0.82 |
| Female | NHL unknown lineage | NHW | Female | NHL unknown lineage | All | 0.36 |
| Female | NHL unknown lineage | NHB | Female | NHL unknown lineage | All | 0.73 |
| Female | Mature B-cell NHL | All | Female | Mature B-cell NHL | NHW | 0.28 |
| Female | Mature B-cell NHL | Hispanic | Female | Mature B-cell NHL | NHB | 0.1 |
| Female | Mature B-cell NHL | Hispanic | Female | Mature B-cell NHL | NHW | 0.05 |
| Male | Mature B-cell NHL | All | Male | Mature B-cell NHL | Hispanic | 0.13 |
| Male | Mature B-cell NHL | Hispanic | Male | Mature B-cell NHL | NHB | 0.12 |
| Male | Mature B-cell NHL | Hispanic | Male | Mature B-cell NHL | NHW | 0.06 |
| Both | Mature B-cell NHL | All | Both | Mature B-cell NHL | Hispanic | 0.35 |
| Both | Mature B-cell NHL | Hispanic | Both | Mature B-cell NHL | NHW | 0.46 |
| Female | Mature T-cell NHL | All | Female | Mature T-cell NHL | Hispanic | 0.37 |
| Female | Mature T-cell NHL | All | Female | Mature T-cell NHL | NHW | 0.84 |
| Female | Mature T-cell NHL | Hispanic | Female | Mature T-cell NHL | NHW | 0.29 |
| Male | Mature T-cell NHL | All | Male | Mature T-cell NHL | Hispanic | 0.73 |
| Male | Mature T-cell NHL | All | Male | Mature T-cell NHL | NHW | 0.26 |
| Male | Mature T-cell NHL | Hispanic | Male | Mature T-cell NHL | NHB | 0.45 |
| Male | Mature T-cell NHL | Hispanic | Male | Mature T-cell NHL | NHW | 0.68 |
| Male | Mature T-cell NHL | NHB | Male | Mature T-cell NHL | NHW | 0.07 |
| Both | Mature T-cell NHL | All | Both | Mature T-cell NHL | Hispanic | 0.55 |
| Both | Mature T-cell NHL | All | Both | Mature T-cell NHL | NHW | 0.58 |
| Both | Mature T-cell NHL | Hispanic | Both | Mature T-cell NHL | NHW | 0.18 |
| Female | NHL | All | Female | NHL | NHW | 0.38 |
| Female | NHL | Hispanic | Female | NHL | NHW | 0.13 |
| Male | NHL | All | Male | NHL | Hispanic | 0.32 |
| Male | NHL | All | Male | NHL | NHW | 0.05 |
| Male | NHL | Hispanic | Male | NHL | NHB | 0.12 |
| Male | NHL | Hispanic | Male | NHL | NHW | 0.21 |
| Both | NHL | All | Both | NHL | Hispanic | 0.69 |
| Both | NHL | All | Both | NHL | NHW | 0.24 |
| Both | NHL | Hispanic | Both | NHL | NHW | 0.4 |
| Female | B-cell NHL | All | Female | B-cell NHL | NHW | 0.7 |
| Female | B-cell NHL | Hispanic | Female | B-cell NHL | NHB | 0.13 |
| Male | B-cell NHL | All | Male | B-cell NHL | Hispanic | 0.21 |
| Male | B-cell NHL | Hispanic | Male | B-cell NHL | NHB | 0.11 |
| Male | B-cell NHL | Hispanic | Male | B-cell NHL | NHW | 0.1 |
| Both | B-cell NHL | All | Both | B-cell NHL | Hispanic | 0.39 |
| Both | B-cell NHL | Hispanic | Both | B-cell NHL | NHW | 0.38 |
| Female | B-cell NHL NOS | All | Female | B-cell NHL NOS | Hispanic | 0.38 |
| Female | B-cell NHL NOS | All | Female | B-cell NHL NOS | NHB | 0.26 |
| Female | B-cell NHL NOS | All | Female | B-cell NHL NOS | NHW | 0.66 |
| Female | B-cell NHL NOS | Hispanic | Female | B-cell NHL NOS | NHB | 0.86 |
| Female | B-cell NHL NOS | NHB | Female | B-cell NHL NOS | NHW | 0.09 |
| Male | B-cell NHL NOS | All | Male | B-cell NHL NOS | Hispanic | 0.98 |
| Male | B-cell NHL NOS | All | Male | B-cell NHL NOS | NHB | 0.57 |
| Male | B-cell NHL NOS | All | Male | B-cell NHL NOS | NHW | 0.27 |
| Male | B-cell NHL NOS | Hispanic | Male | B-cell NHL NOS | NHB | 0.36 |
| Male | B-cell NHL NOS | Hispanic | Male | B-cell NHL NOS | NHW | 0.88 |
| Male | B-cell NHL NOS | NHB | Male | B-cell NHL NOS | NHW | 0.22 |
| Both | B-cell NHL NOS | All | Both | B-cell NHL NOS | Hispanic | 0.88 |
| Both | B-cell NHL NOS | All | Both | B-cell NHL NOS | NHB | 0.72 |
| Both | B-cell NHL NOS | All | Both | B-cell NHL NOS | NHW | 0.37 |
| Both | B-cell NHL NOS | Hispanic | Both | B-cell NHL NOS | NHB | 0.86 |
| Both | B-cell NHL NOS | Hispanic | Both | B-cell NHL NOS | NHW | 0.62 |
| Both | B-cell NHL NOS | NHB | Both | B-cell NHL NOS | NHW | 0.48 |
| Female | T-cell NHL | All | Female | T-cell NHL | Hispanic | 0.06 |
| Female | T-cell NHL | All | Female | T-cell NHL | NHB | 0.06 |
| Female | T-cell NHL | All | Female | T-cell NHL | NHW | 0.92 |
| Female | T-cell NHL | Hispanic | Female | T-cell NHL | NHW | 0.21 |
| Male | T-cell NHL | All | Male | T-cell NHL | Hispanic | 0.76 |
| Male | T-cell NHL | All | Male | T-cell NHL | NHW | 0.06 |
| Male | T-cell NHL | Hispanic | Male | T-cell NHL | NHB | 0.42 |
| Male | T-cell NHL | Hispanic | Male | T-cell NHL | NHW | 0.65 |
| Male | T-cell NHL | NHB | Male | T-cell NHL | NHW | 0.05 |
| Both | T-cell NHL | All | Both | T-cell NHL | Hispanic | 0.41 |
| Both | T-cell NHL | All | Both | T-cell NHL | NHW | 0.44 |
| Both | T-cell NHL | Hispanic | Both | T-cell NHL | NHW | 0.15 |
| Female | NHL unknown lineage | All | Female | NHL unknown lineage | NHB | 0.74 |
| Female | NHL unknown lineage | All | Female | NHL unknown lineage | NHW | 0.36 |
| Female | NHL unknown lineage | NHB | Female | NHL unknown lineage | NHW | 0.81 |
| Male | NHL unknown lineage | All | Male | NHL unknown lineage | Hispanic | 0.26 |
| Male | NHL unknown lineage | All | Male | NHL unknown lineage | NHW | 0.42 |
| Male | NHL unknown lineage | Hispanic | Male | NHL unknown lineage | NHW | 0.42 |
| Both | NHL unknown lineage | All | Both | NHL unknown lineage | Hispanic | 0.05 |
| Both | NHL unknown lineage | All | Both | NHL unknown lineage | NHW | 0.45 |
| Both | NHL unknown lineage | Hispanic | Both | NHL unknown lineage | NHW | 0.07 |
| Both | NHL unknown lineage | NHB | Both | NHL unknown lineage | NHW | 0.15 |
| Female | Precursor B-cell NHL | All | Female | Precursor B-cell NHL | Hispanic | 0.65 |
| Female | Precursor B-cell NHL | All | Female | Precursor B-cell NHL | NHB | 0.18 |
| Female | Precursor B-cell NHL | Hispanic | Female | Precursor B-cell NHL | NHB | 0.35 |
| Female | Precursor B-cell NHL | Hispanic | Female | Precursor B-cell NHL | NHW | 0.25 |
| Female | Precursor B-cell NHL | NHB | Female | Precursor B-cell NHL | NHW | 0.83 |
| Male | Precursor B-cell NHL | All | Male | Precursor B-cell NHL | Hispanic | 0.07 |
| Male | Precursor B-cell NHL | NHB | Male | Precursor B-cell NHL | NHW | 0.06 |
| Both | Precursor B-cell NHL | All | Both | Precursor B-cell NHL | Hispanic | 0.24 |
| Both | Precursor B-cell NHL | NHB | Both | Precursor B-cell NHL | NHW | 0.42 |
| Female | Precursor T-cell NHL | All | Female | Precursor T-cell NHL | Hispanic | 0.34 |
| Female | Precursor T-cell NHL | All | Female | Precursor T-cell NHL | NHW | 0.72 |
| Female | Precursor T-cell NHL | Hispanic | Female | Precursor T-cell NHL | NHW | 0.32 |
| Male | Precursor T-cell NHL | All | Male | Precursor T-cell NHL | Hispanic | 0.6 |
| Male | Precursor T-cell NHL | All | Male | Precursor T-cell NHL | NHB | 0.64 |
| Male | Precursor T-cell NHL | Hispanic | Male | Precursor T-cell NHL | NHB | 0.18 |
| Male | Precursor T-cell NHL | Hispanic | Male | Precursor T-cell NHL | NHW | 0.53 |
| Male | Precursor T-cell NHL | NHB | Male | Precursor T-cell NHL | NHW | 0.17 |
| Both | Precursor T-cell NHL | All | Both | Precursor T-cell NHL | Hispanic | 0.72 |
| Both | Precursor T-cell NHL | All | Both | Precursor T-cell NHL | NHB | 0.22 |
| Both | Precursor T-cell NHL | All | Both | Precursor T-cell NHL | NHW | 0.07 |
| Both | Precursor T-cell NHL | Hispanic | Both | Precursor T-cell NHL | NHW | 0.65 |
| Both | Precursor T-cell NHL | NHB | Both | Precursor T-cell NHL | NHW | 0.06 |

**Abbreviations**: **NHW:** Non-Hispanic White; **NHB:** Non-Hispanic Black; **NHL**; Non-Hodgkin lymphoma, **NOS**: Not Otherwise Specified.


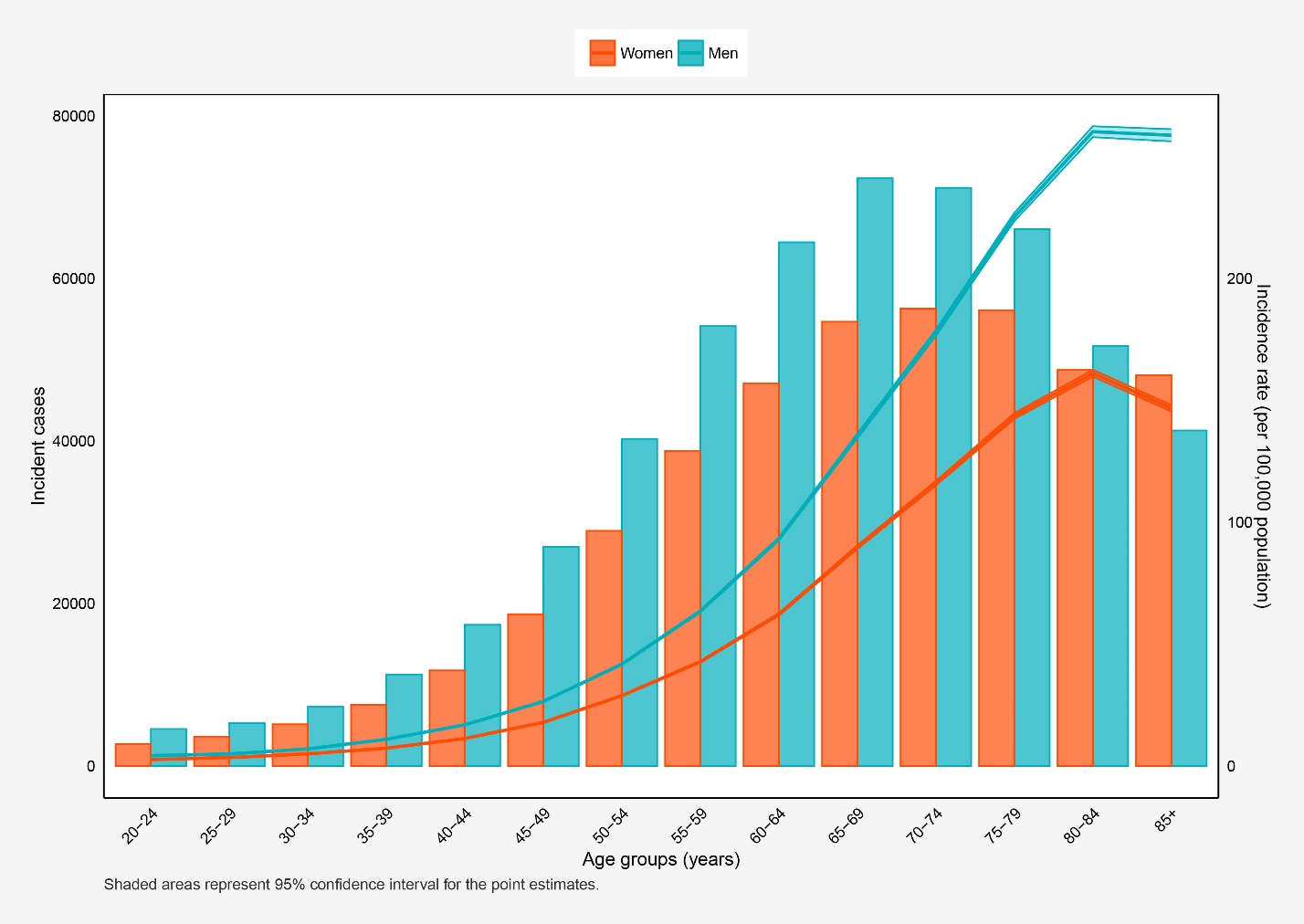


**Figure S1.** Incident cases and incidence rate of non-Hodgkin lymphoma in the United States among males and females in each age group.


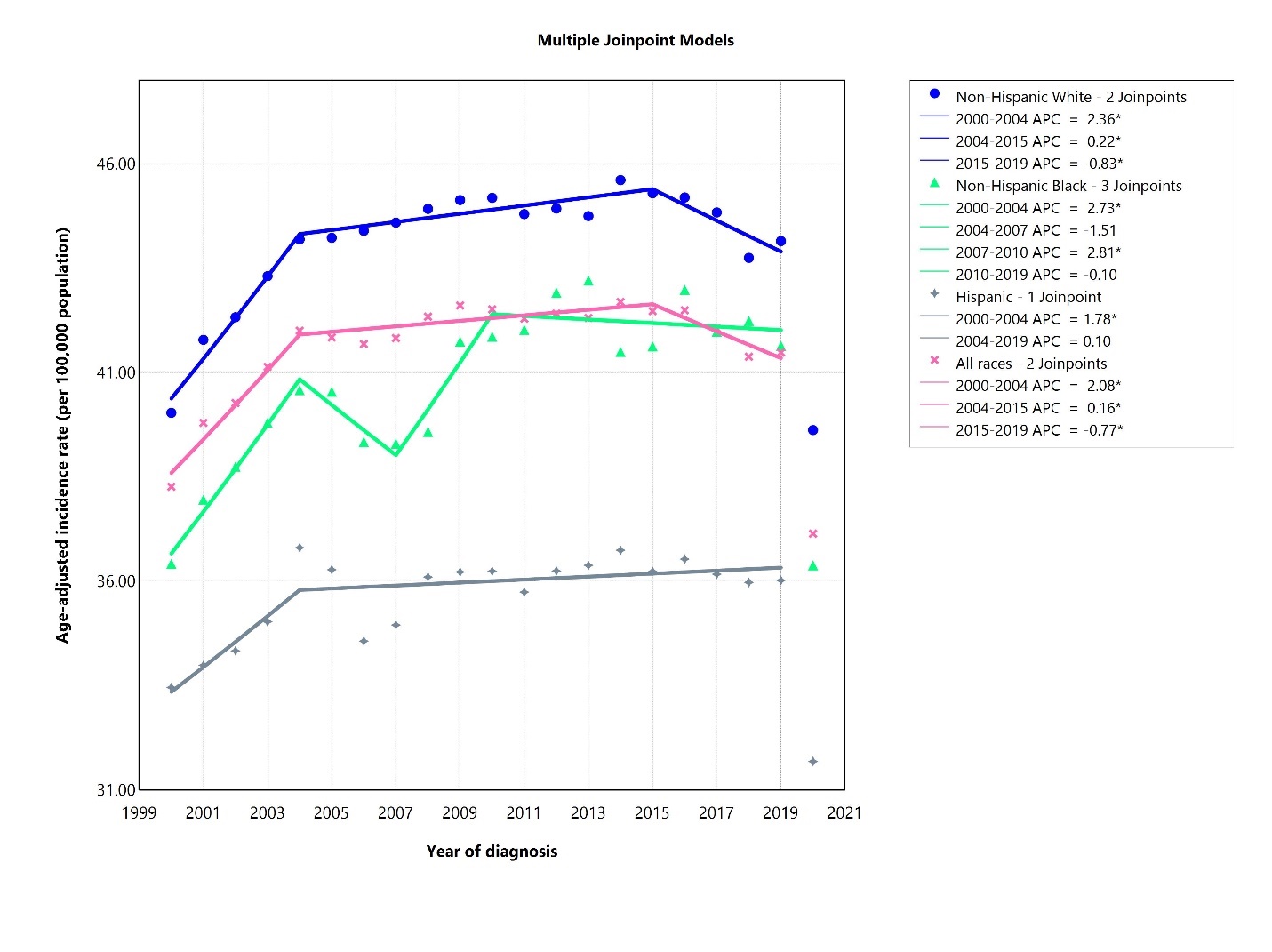


**Figure S2.** The age-adjusted incidence rate of B-cell non-Hodgkin lymphoma in adults over 2000-2019 and 2020 in the United States, by race. APC: annual percent change. * Represent p-value less than 0.05.


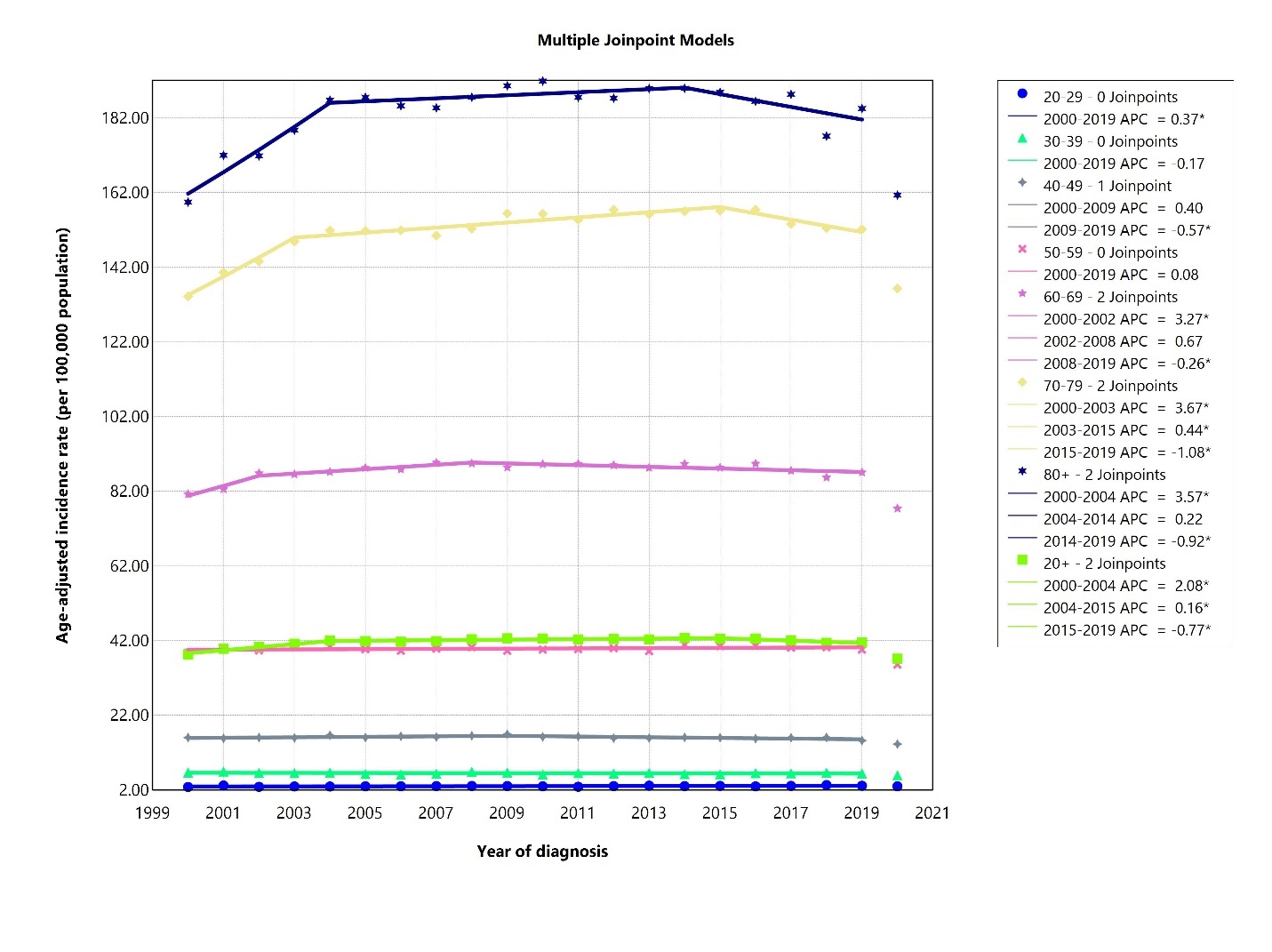


**Figure S3.** The age-adjusted incidence rate of B-cell non-Hodgkin lymphoma in adults over 2000-2019 and 2020 in the United States, by age. APC: annual percent change. * Represents a p-value less than 0.05.


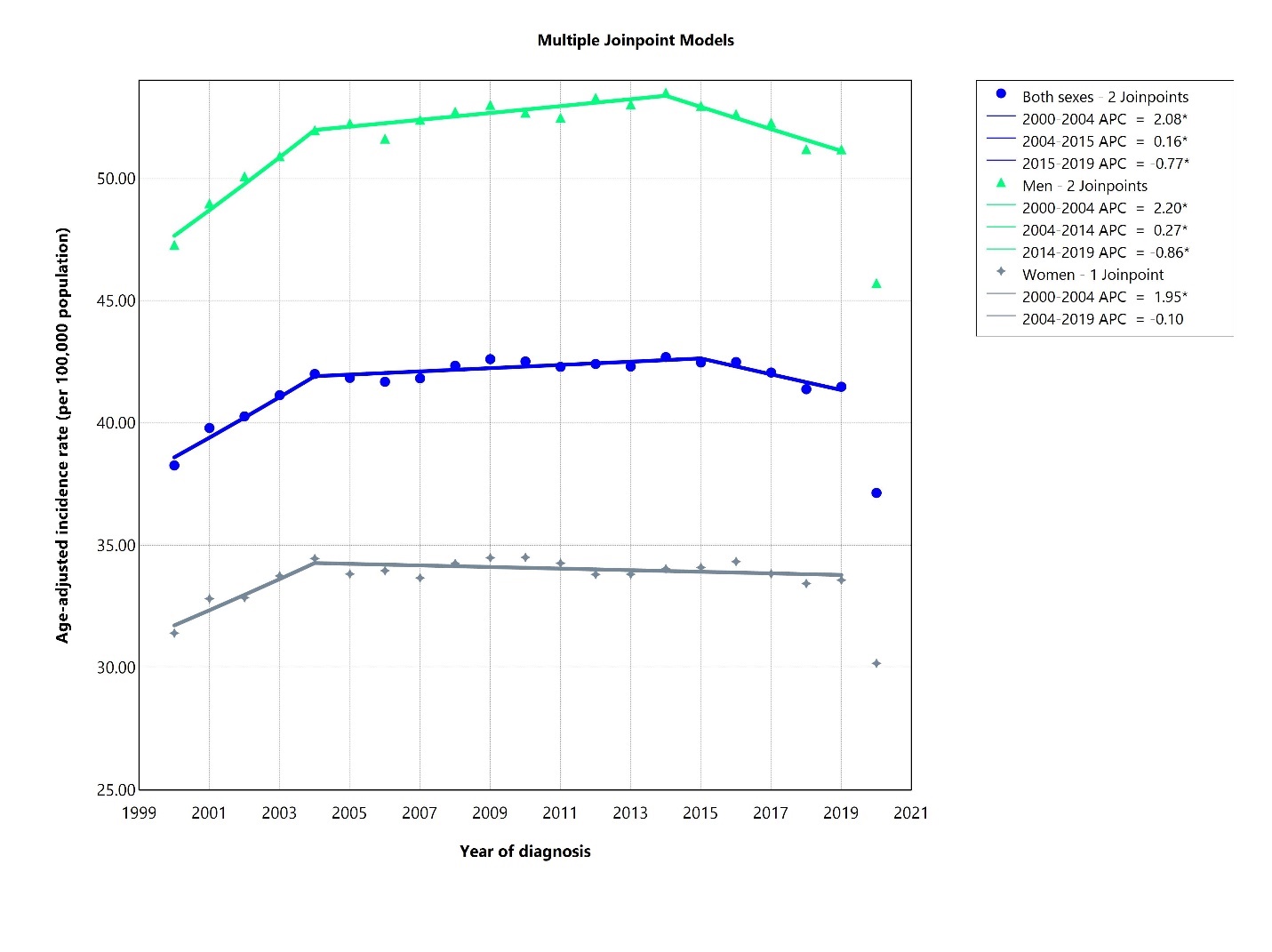


**Figure S4.** The age-adjusted incidence rate of B-cell non-Hodgkin lymphoma in adults over 2000-2019 and 2020 in the United States, by sex. APC: annual percent change. * Represents a p-value less than 0.05.


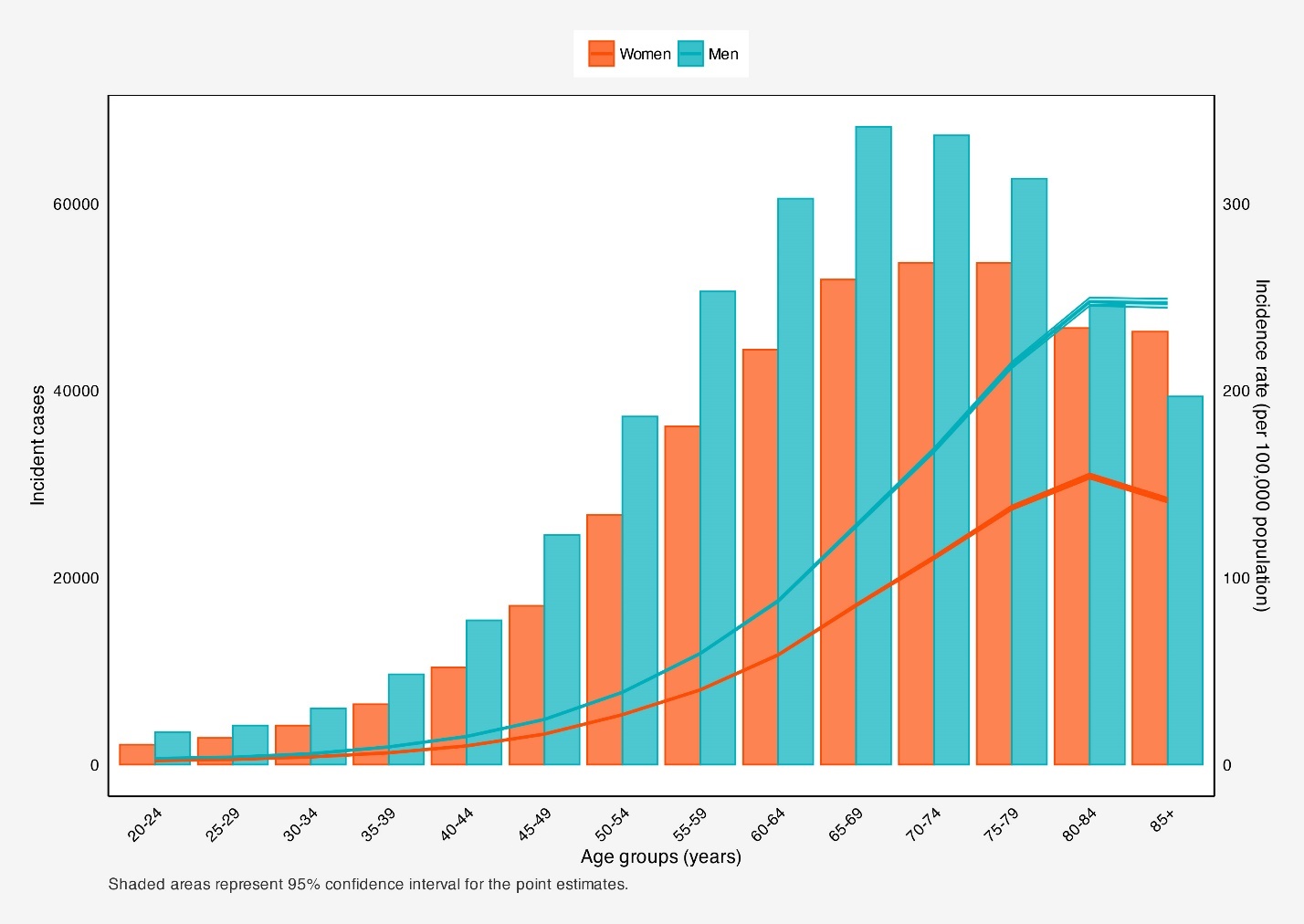


**Figure S5.** Incident cases and incidence rate of B-cell non-Hodgkin lymphoma in the United States among males and females in each age group.


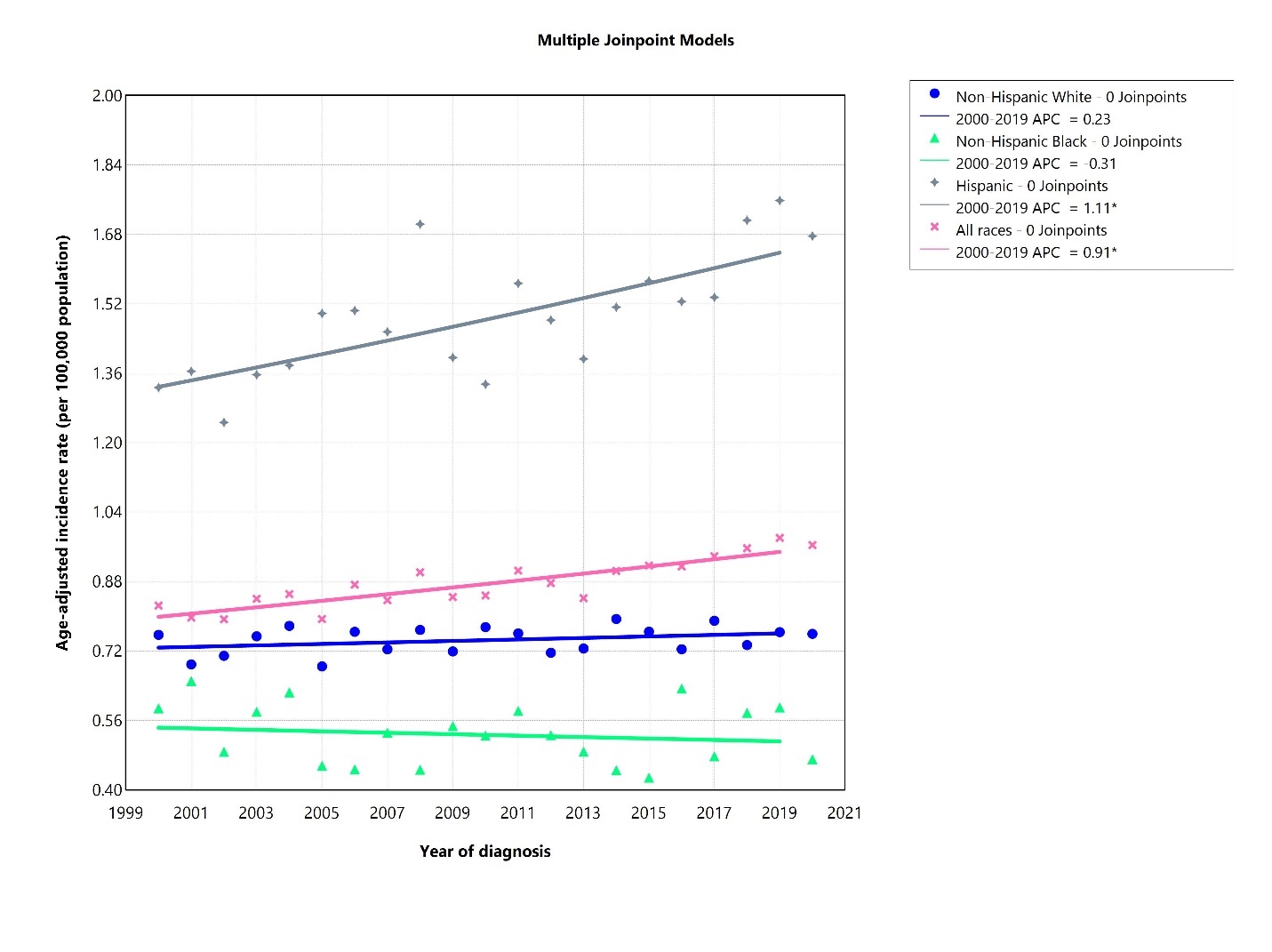


**Figure S6.** The age-adjusted incidence rate of precursor B-cell non-Hodgkin lymphoma in adults over 2000-2019 and 2020 in the United States, by race. APC: annual percent change. * Represents a p-value less than 0.05.


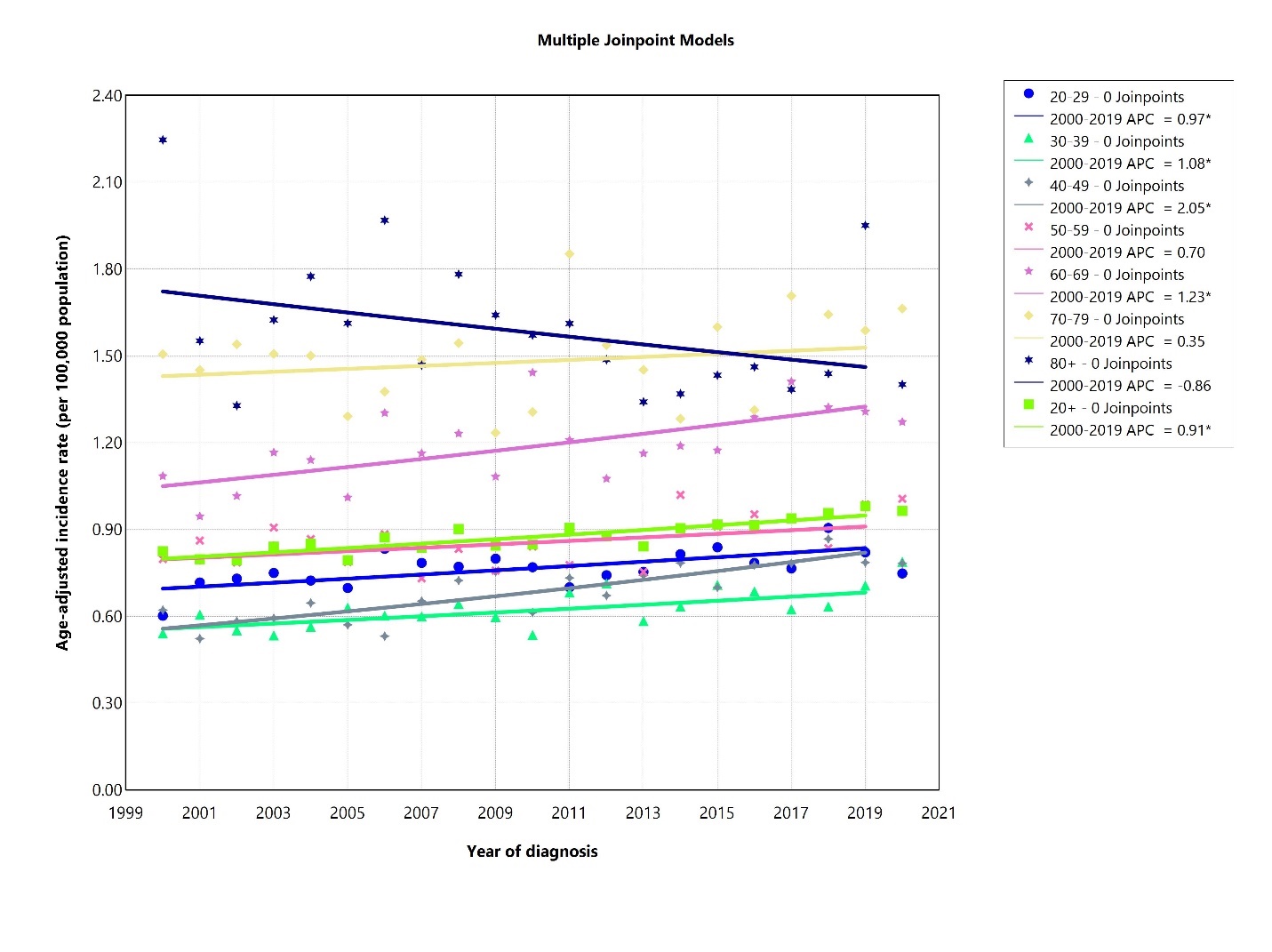


**Figure S7.** The age-adjusted incidence rate of precursor B-cell non-Hodgkin lymphoma in adults over 2000-2019 and 2020 in the United States, by age. APC: annual percent change. * Represents a p-value less than 0.05.


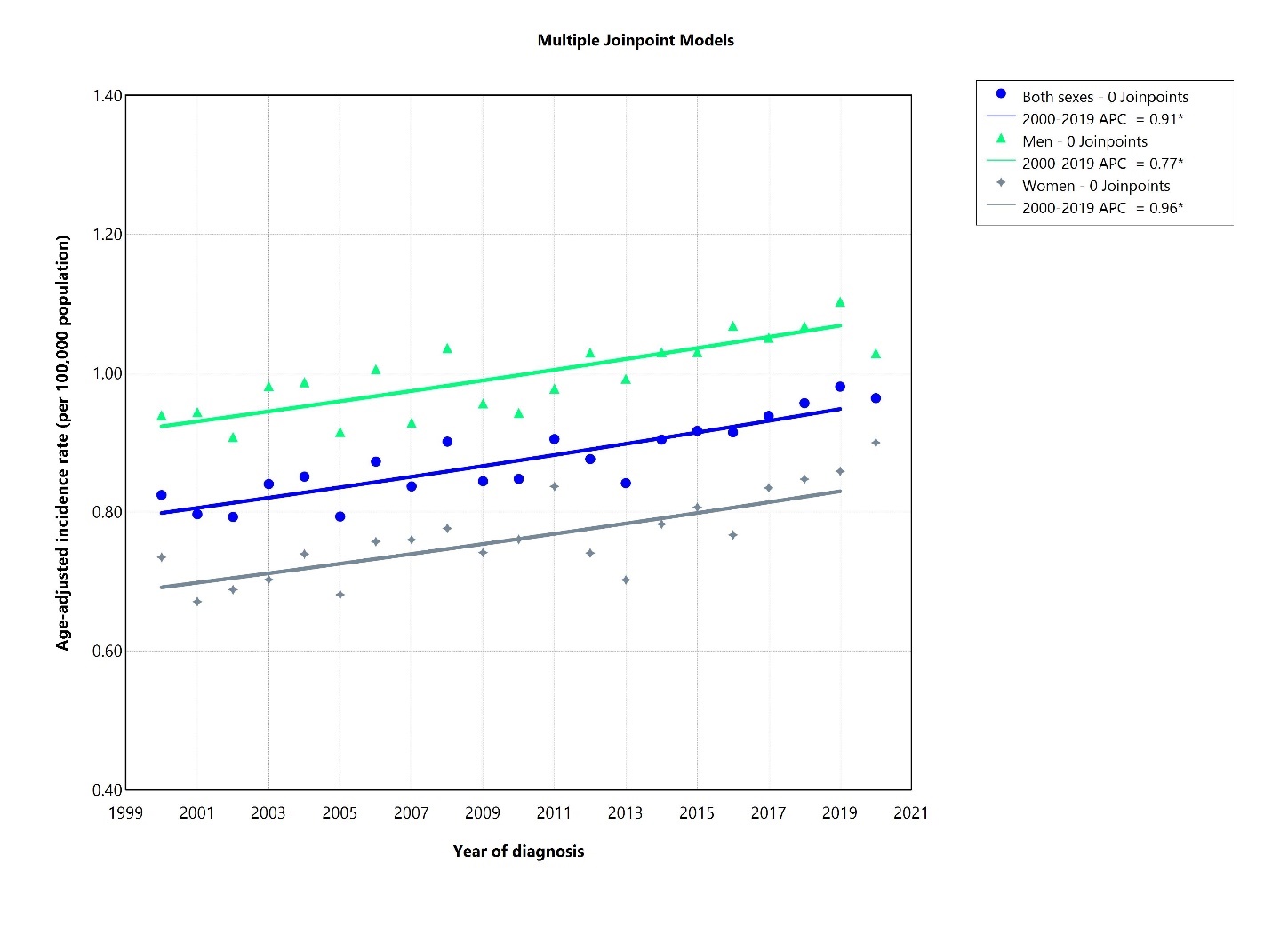


**Figure S8.** The age-adjusted incidence rate of precursor B-cell non-Hodgkin lymphoma in adults over 2000-2019 and 2020 in the United States, by sex. APC: annual percent change. * Represents a p-value less than 0.05.


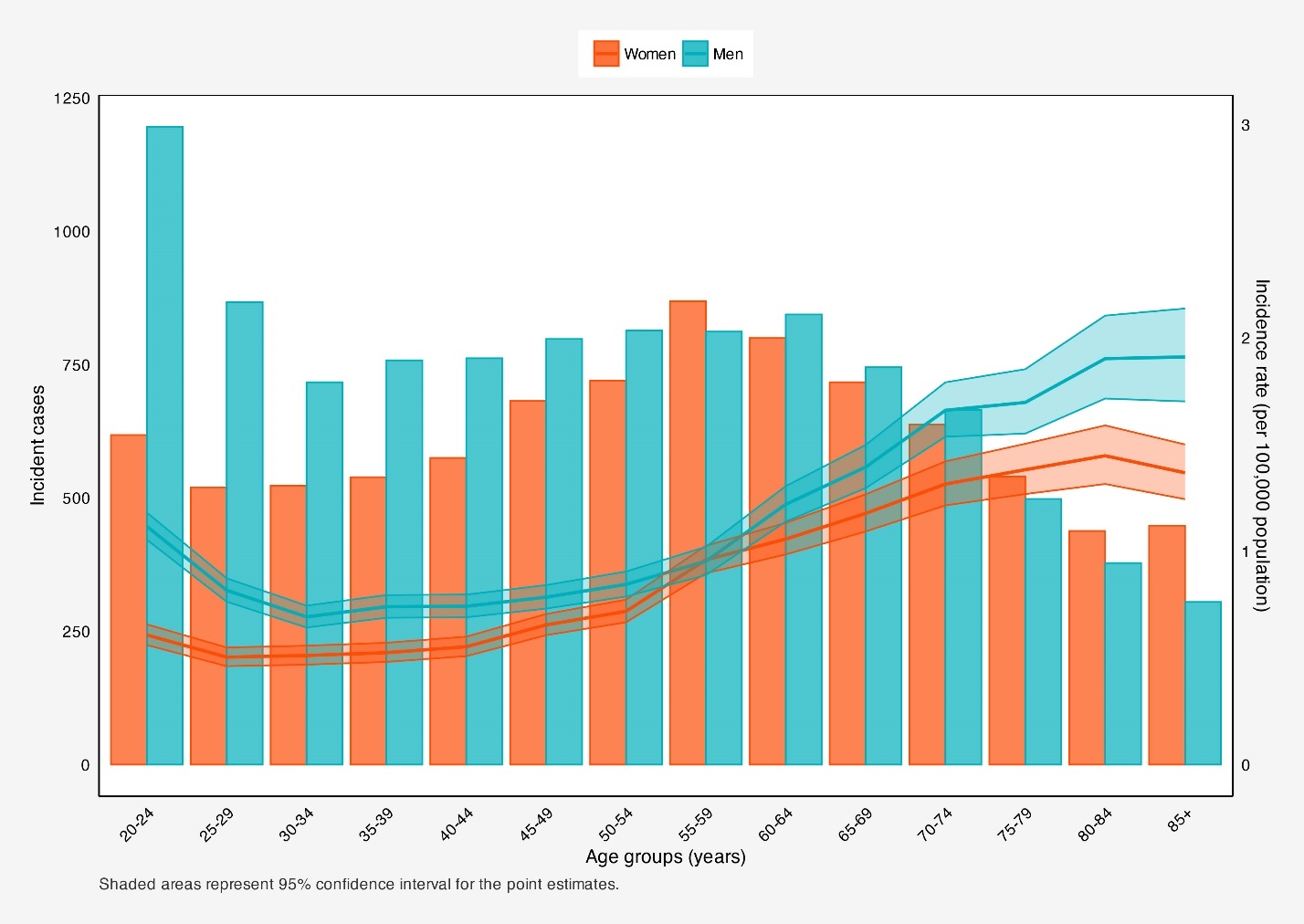


**Figure S9.** Incident cases and incidence rate of precursor B-cell non-Hodgkin lymphoma in the United States among males and females in each age group.


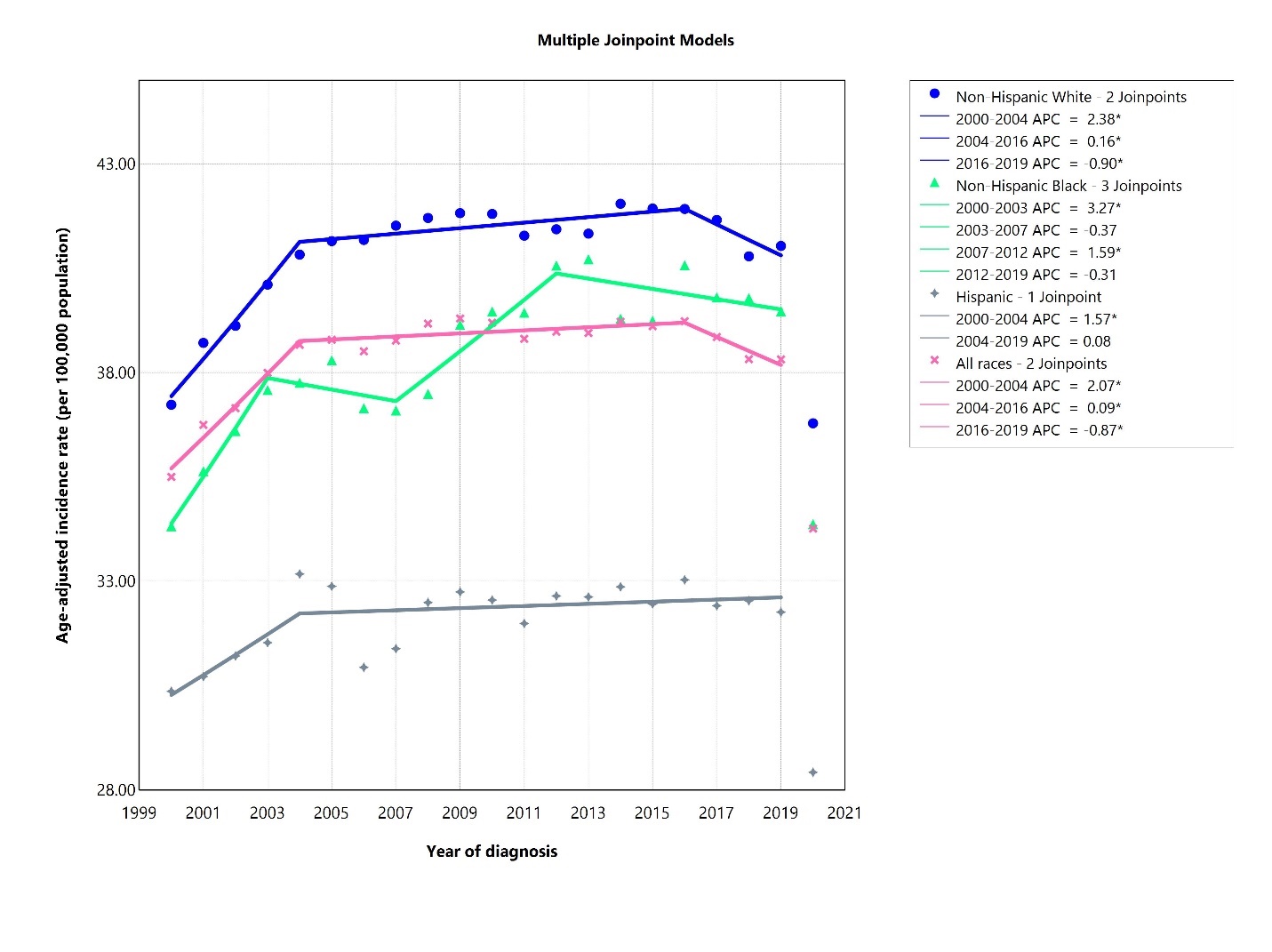


**Figure S10.** The age-adjusted incidence rate of mature B-cell non-Hodgkin lymphoma in adults over 2000-2019 and 2020 in the United States, by race. APC: annual percent change. * Represents a p-value less than 0.05.


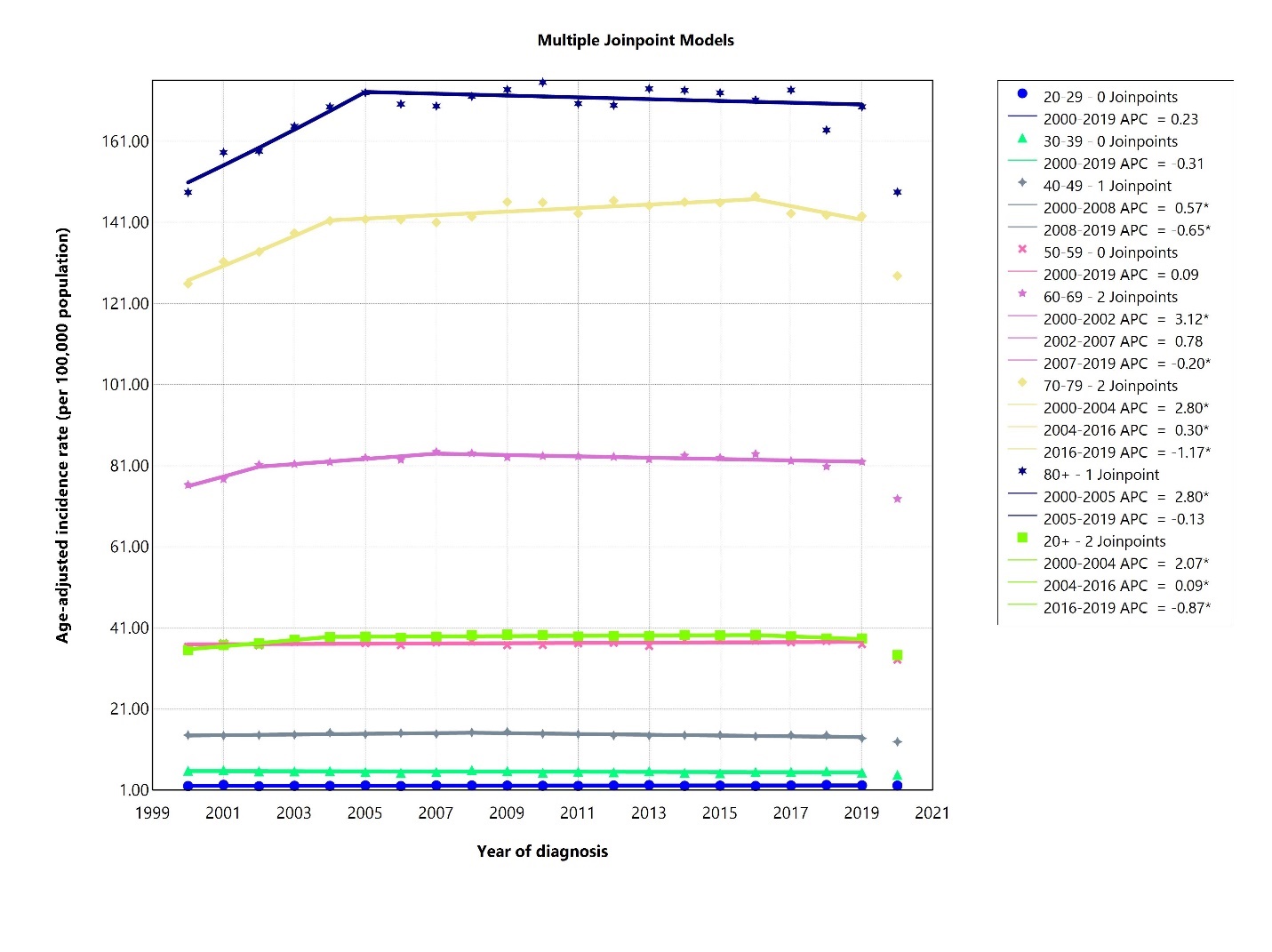


**Figure S11.** The age-adjusted incidence rate of mature B-cell non-Hodgkin lymphoma in adults over 2000-2019 and 2020 in the United States, by age. APC: annual percent change. * Represents a p-value less than 0.05.


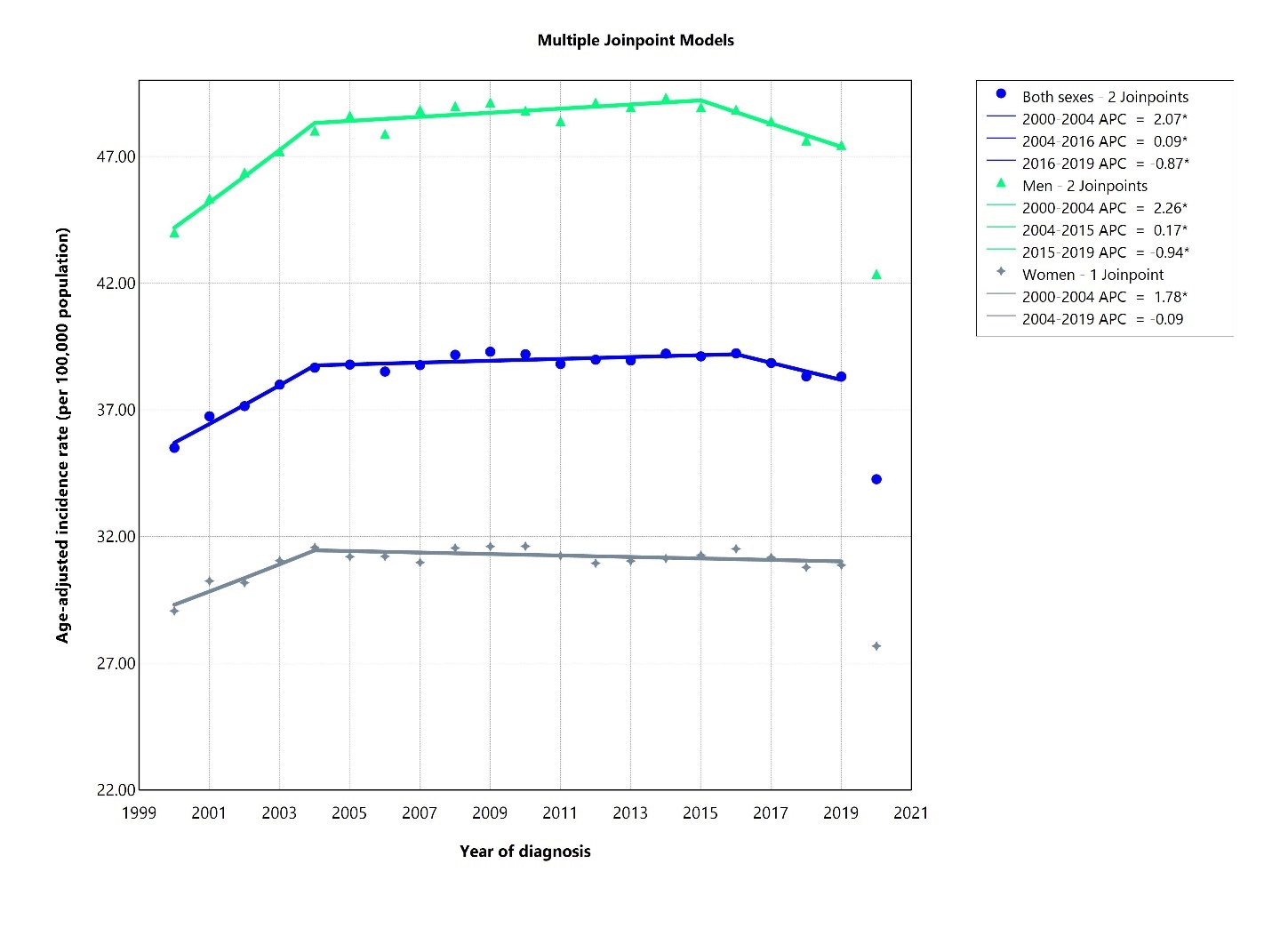


**Figure S12.** The age-adjusted incidence rate of mature B-cell non-Hodgkin lymphoma in adults over 2000-2019 and 2020 in the United States, by sex. APC: annual percent change. * Represents a p-value less than 0.05.


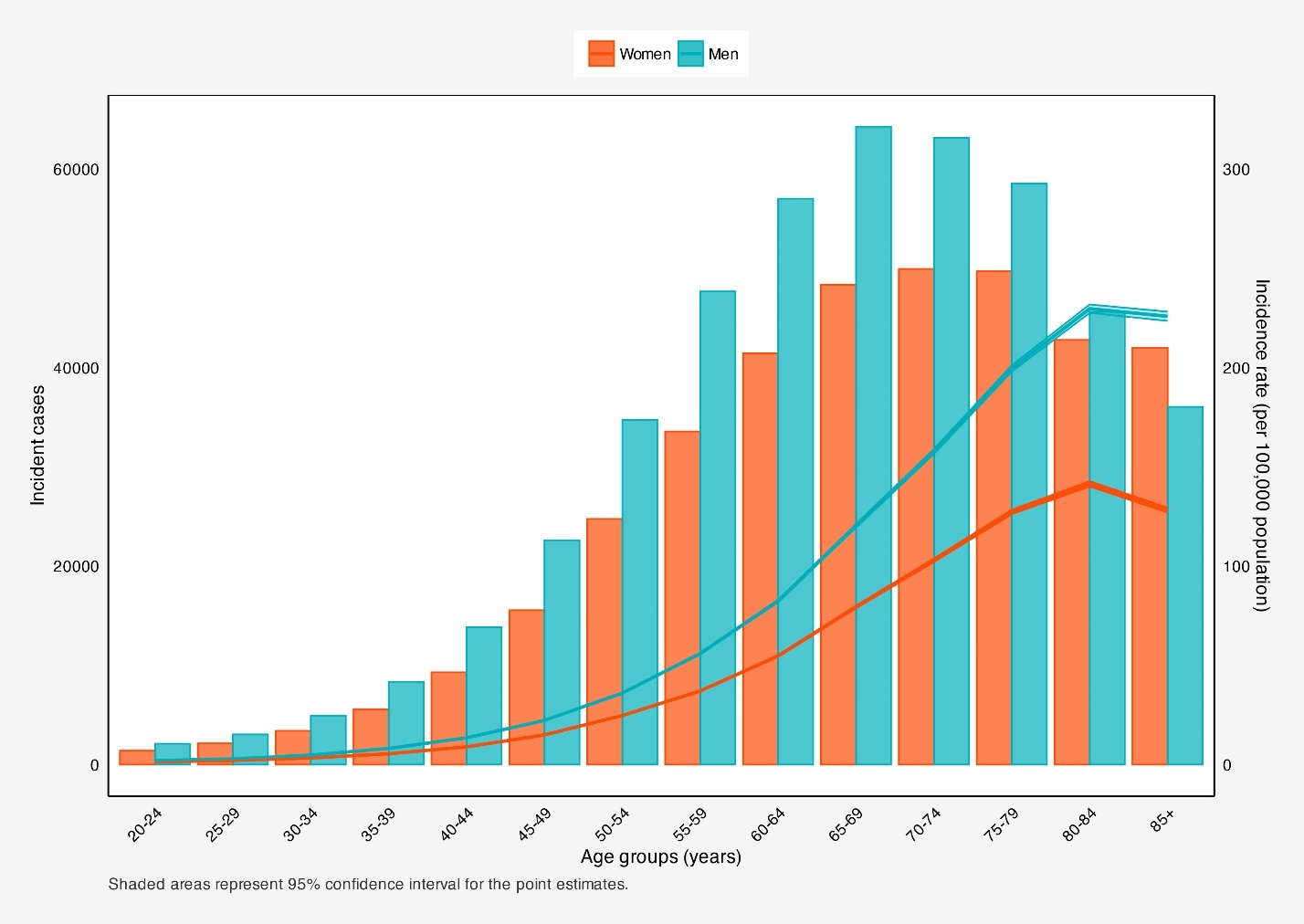


**Figure S13.** Incident cases and incidence rate of mature B-cell non-Hodgkin lymphoma in the United States among males and females in each age group.


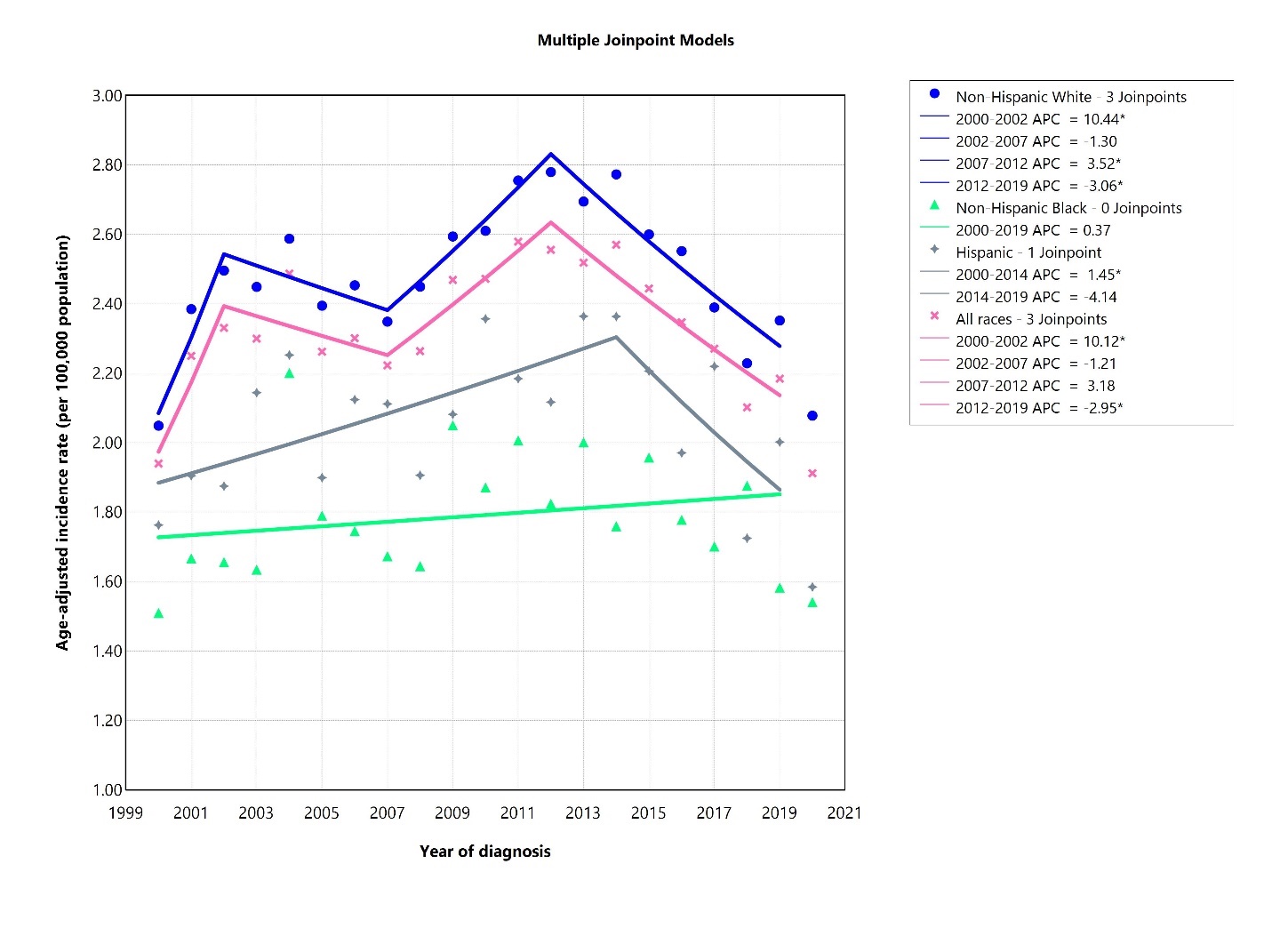


**Figure S14.** The age-adjusted incidence rate of B-cell non-Hodgkin lymphoma not otherwise specified (NOS) in adults over 2000-2019 and 2020 in the United States, by race. APC: annual percent change. * Represents a p-value less than 0.05.


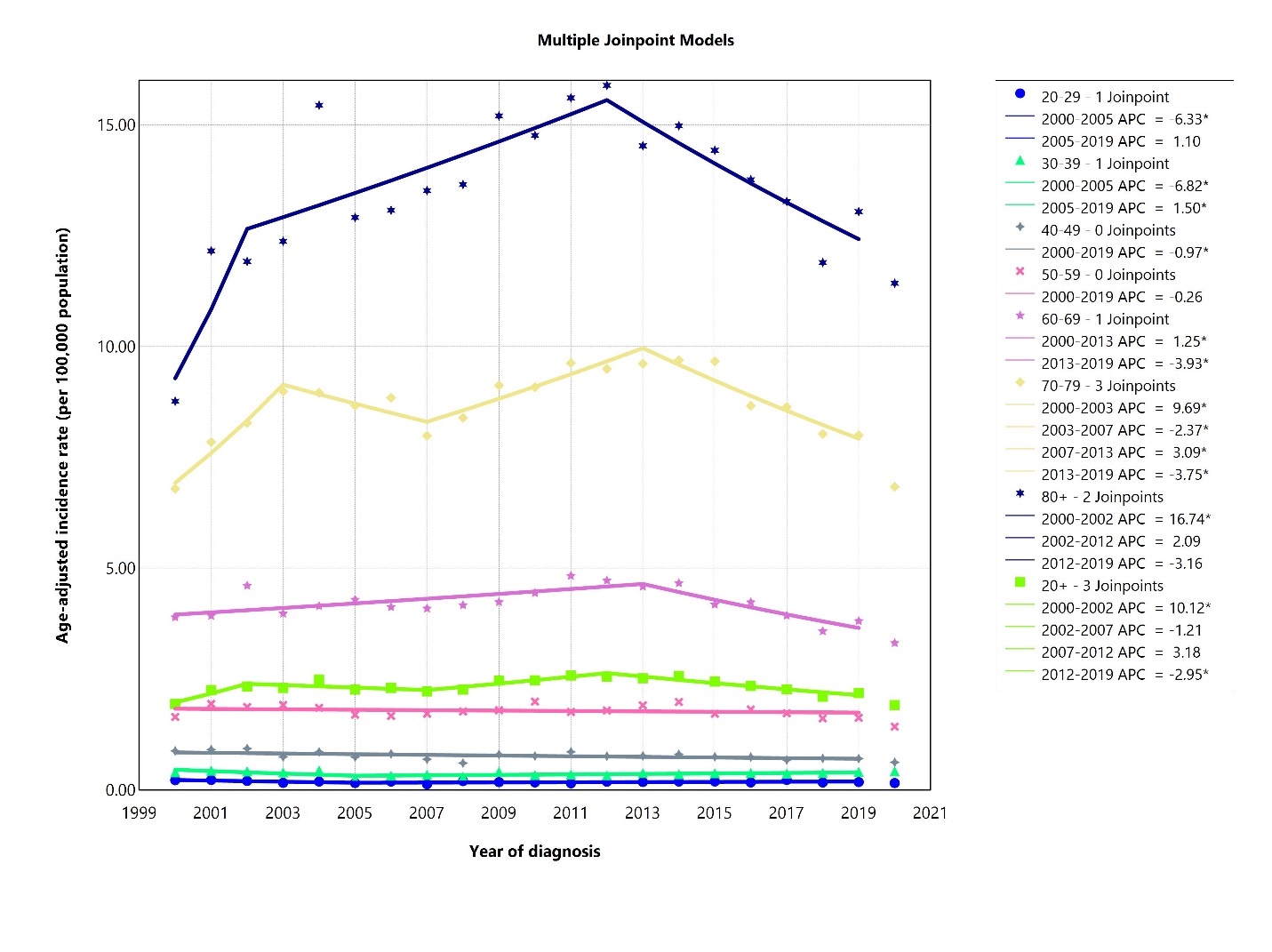


**Figure S15.** The age-adjusted incidence rate of B-cell non-Hodgkin lymphoma not otherwise specified (NOS) in adults over 2000-2019 and 2020 in the United States, by age. APC: annual percent change. * Represents a p-value less than 0.05.


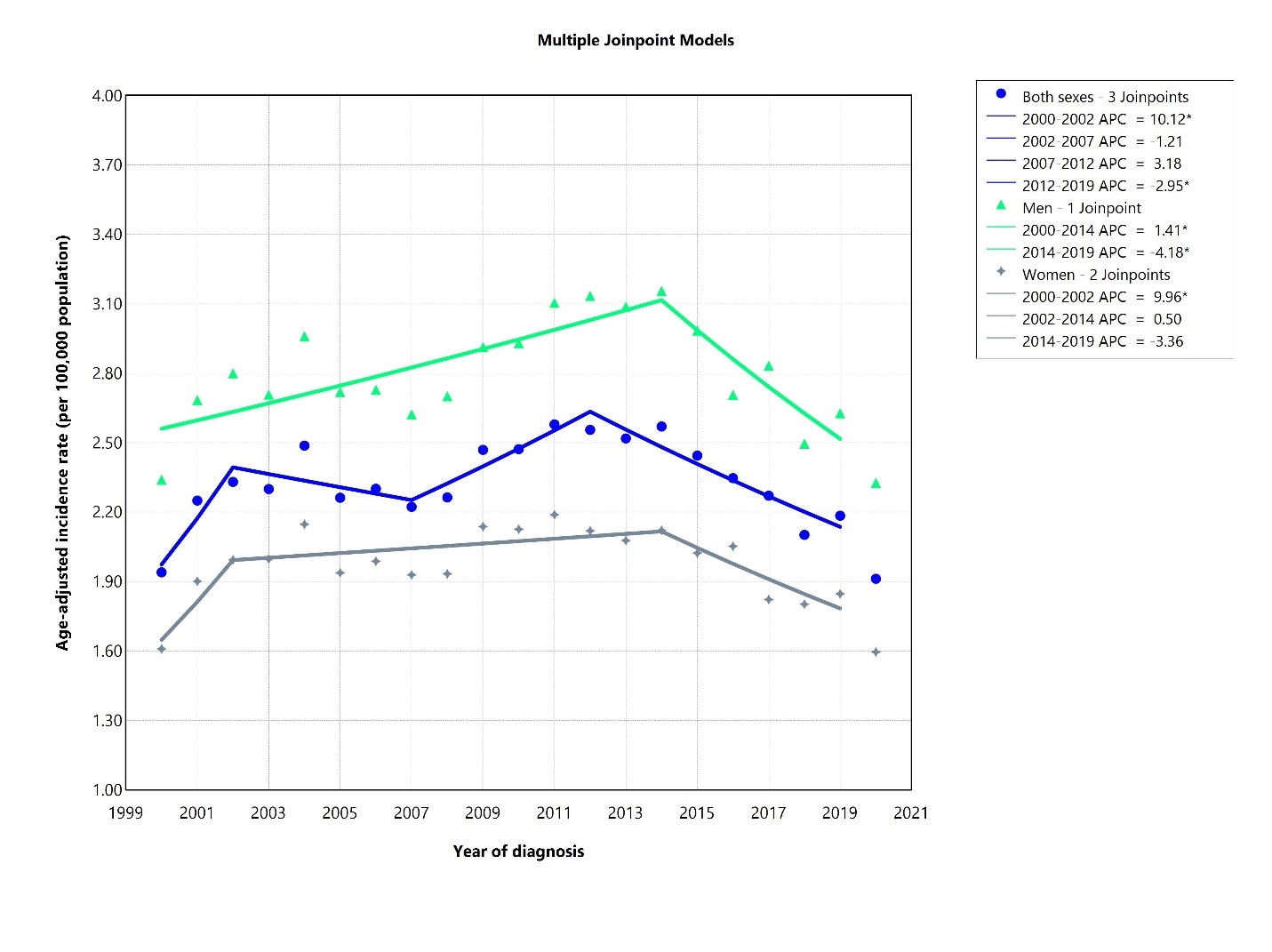


**Figure S16.** The age-adjusted incidence rate of B-cell non-Hodgkin lymphoma not otherwise specified (NOS) in adults over 2000-2019 and 2020 in the United States, by sex. APC: annual percent change. * Represents a p-value less than 0.05.


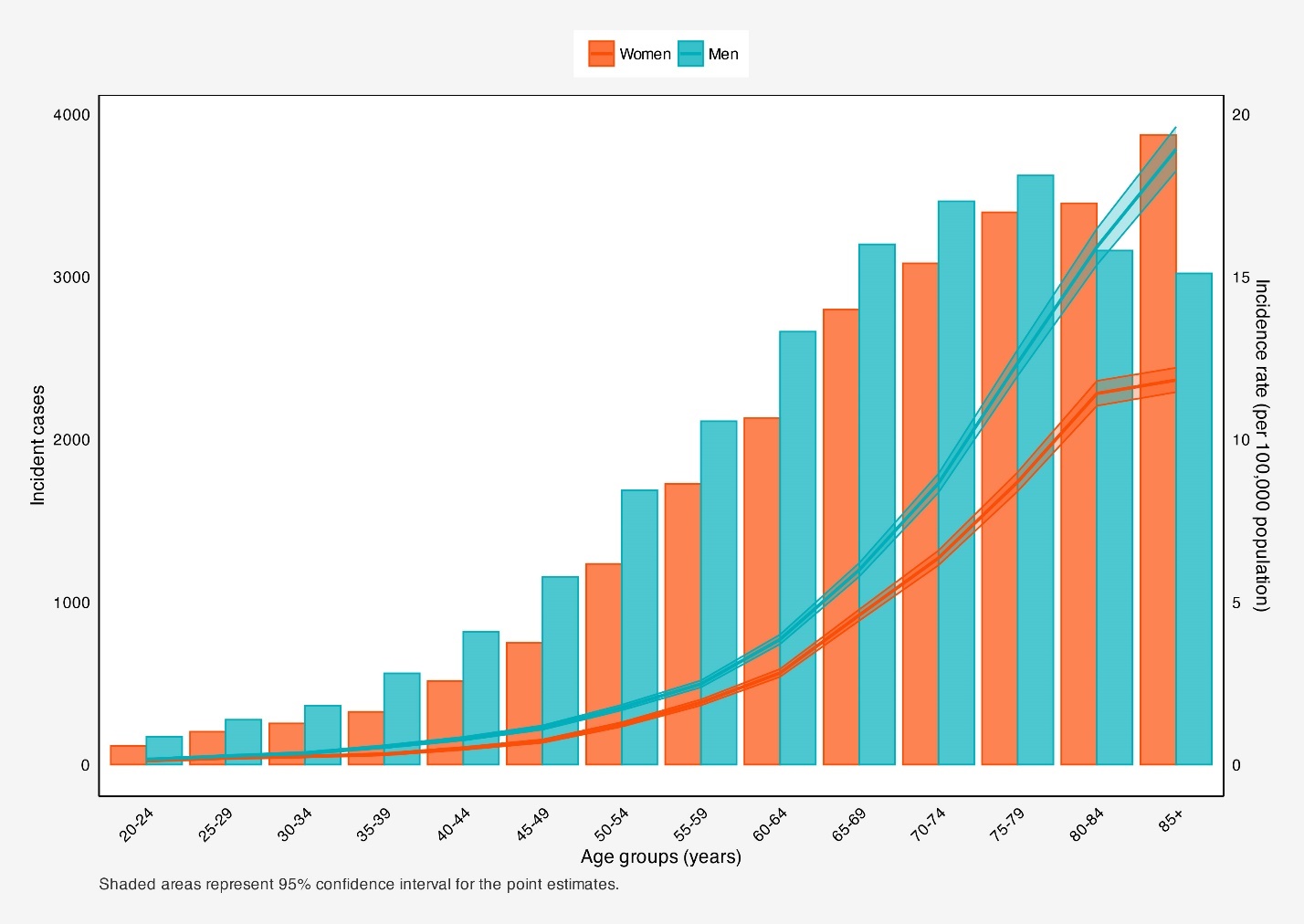


**Figure S17.** Incident cases and incidence rate of B-cell non-Hodgkin lymphoma not otherwise specified (NOS) in the United States among males and females in each age group.


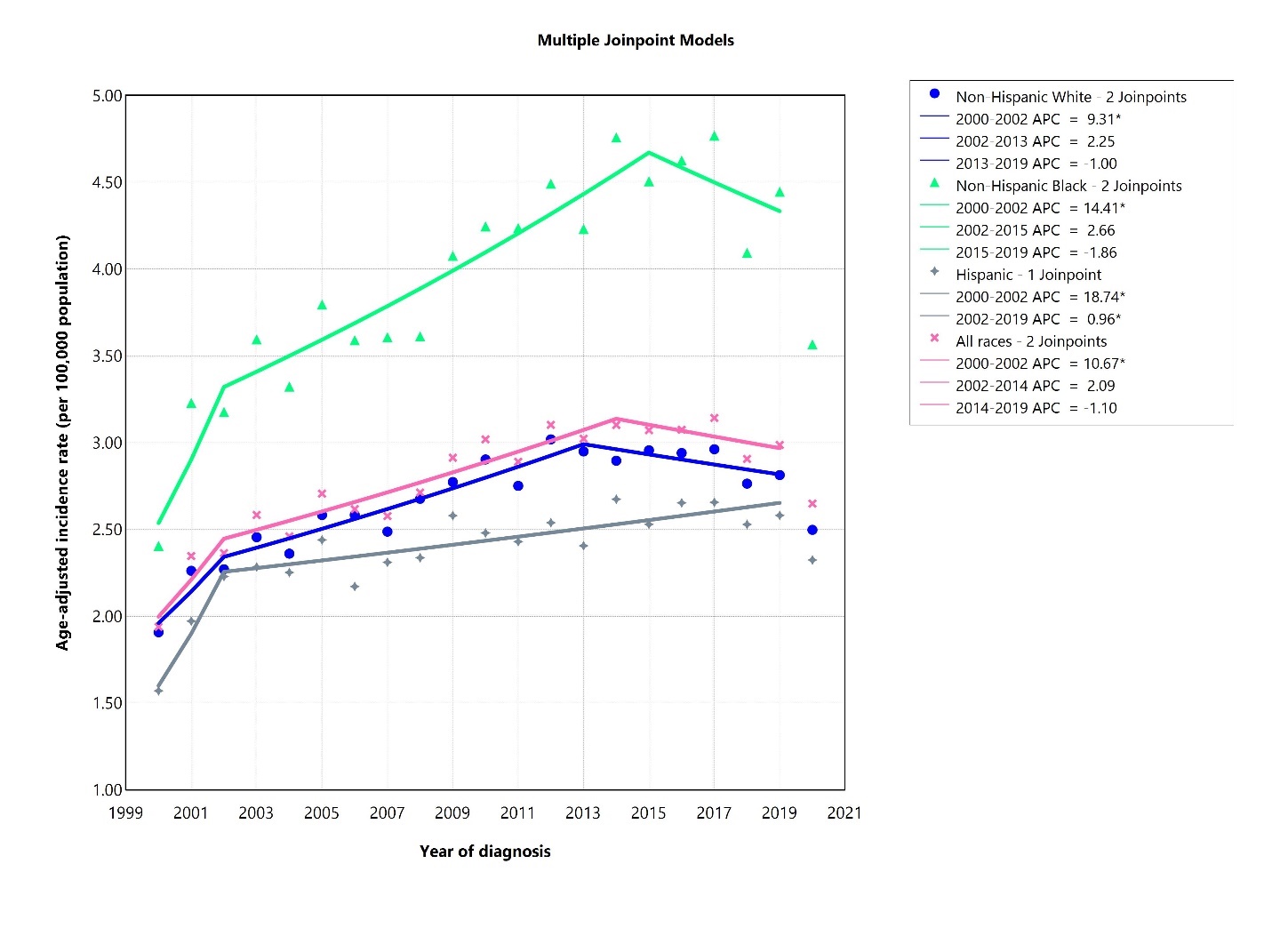


**Figure S18.** The age-adjusted incidence rate of T-cell non-Hodgkin lymphoma in adults over 2000-2019 and 2020 in the United States, by race. APC: annual percent change. * Represents a p-value less than 0.05.


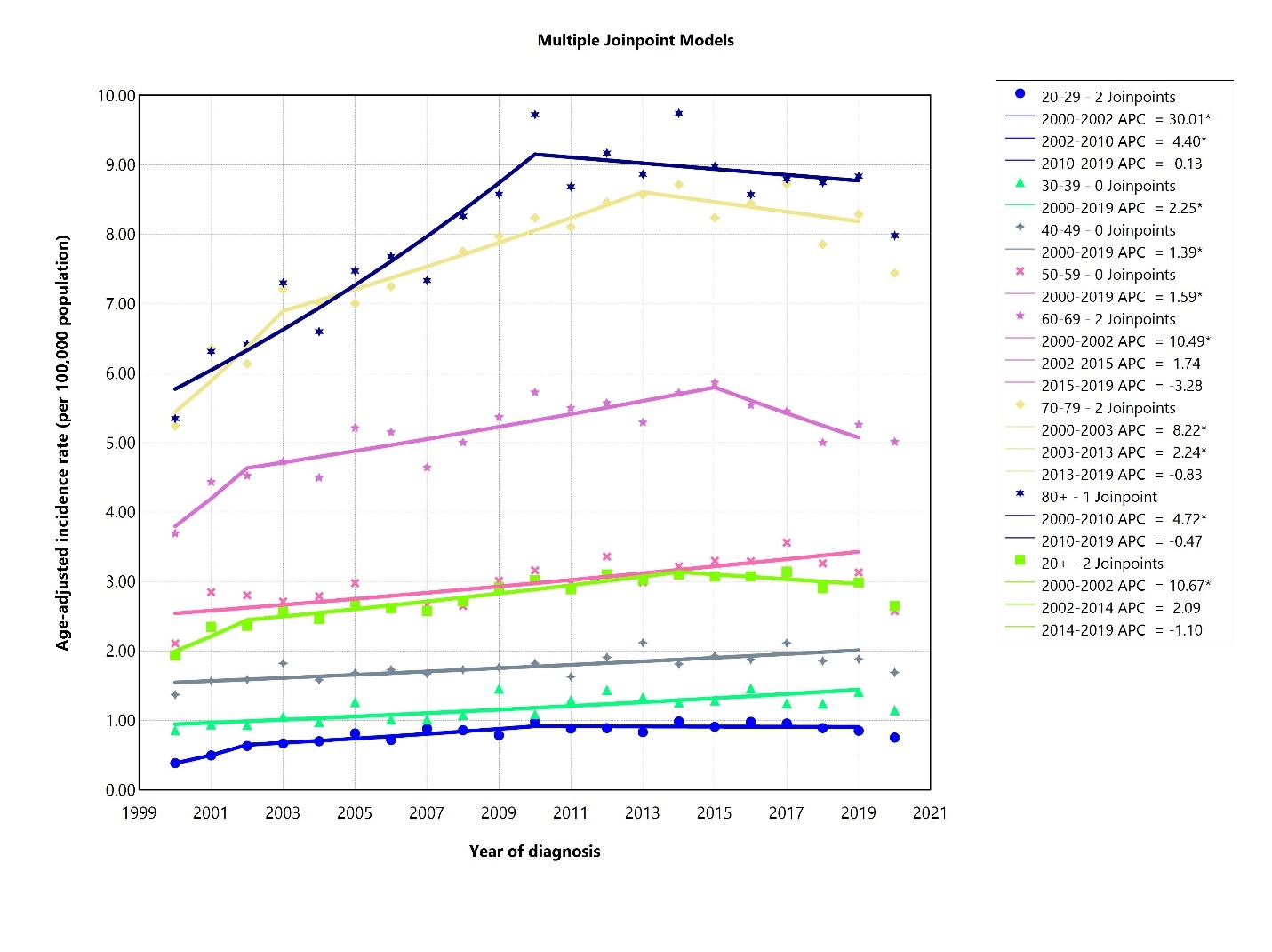


**Figure S19.** The age-adjusted incidence rate of T-cell non-Hodgkin lymphoma in adults over 2000-2019 and 2020 in the United States, by age. APC: annual percent change. * Represents a p-value less than 0.05.


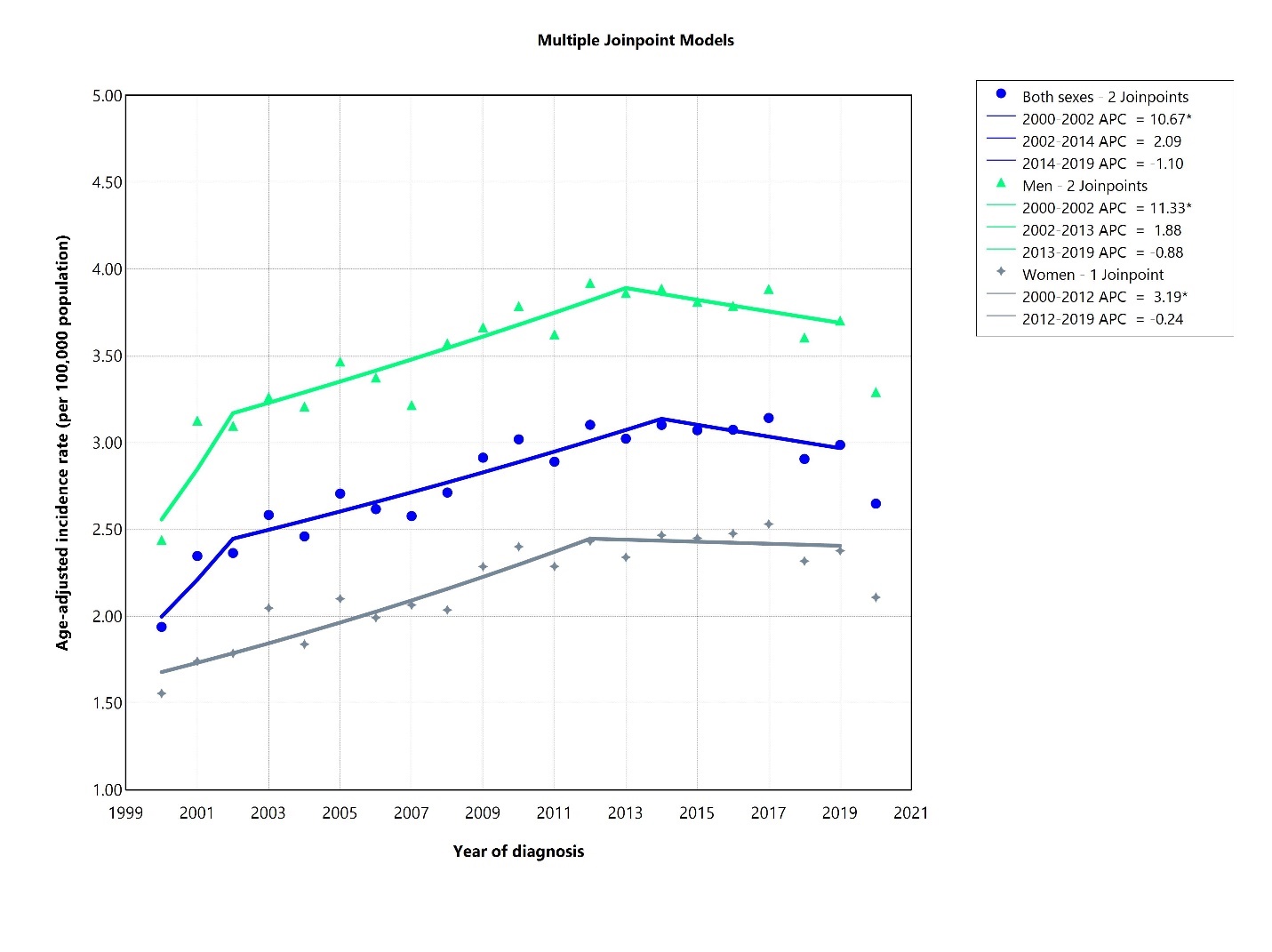


**Figure S20.** The age-adjusted incidence rate of T-cell non-Hodgkin lymphoma in adults over 2000-2019 and 2020 in the United States, by sex. APC: annual percent change. * Represents a p-value less than 0.05.


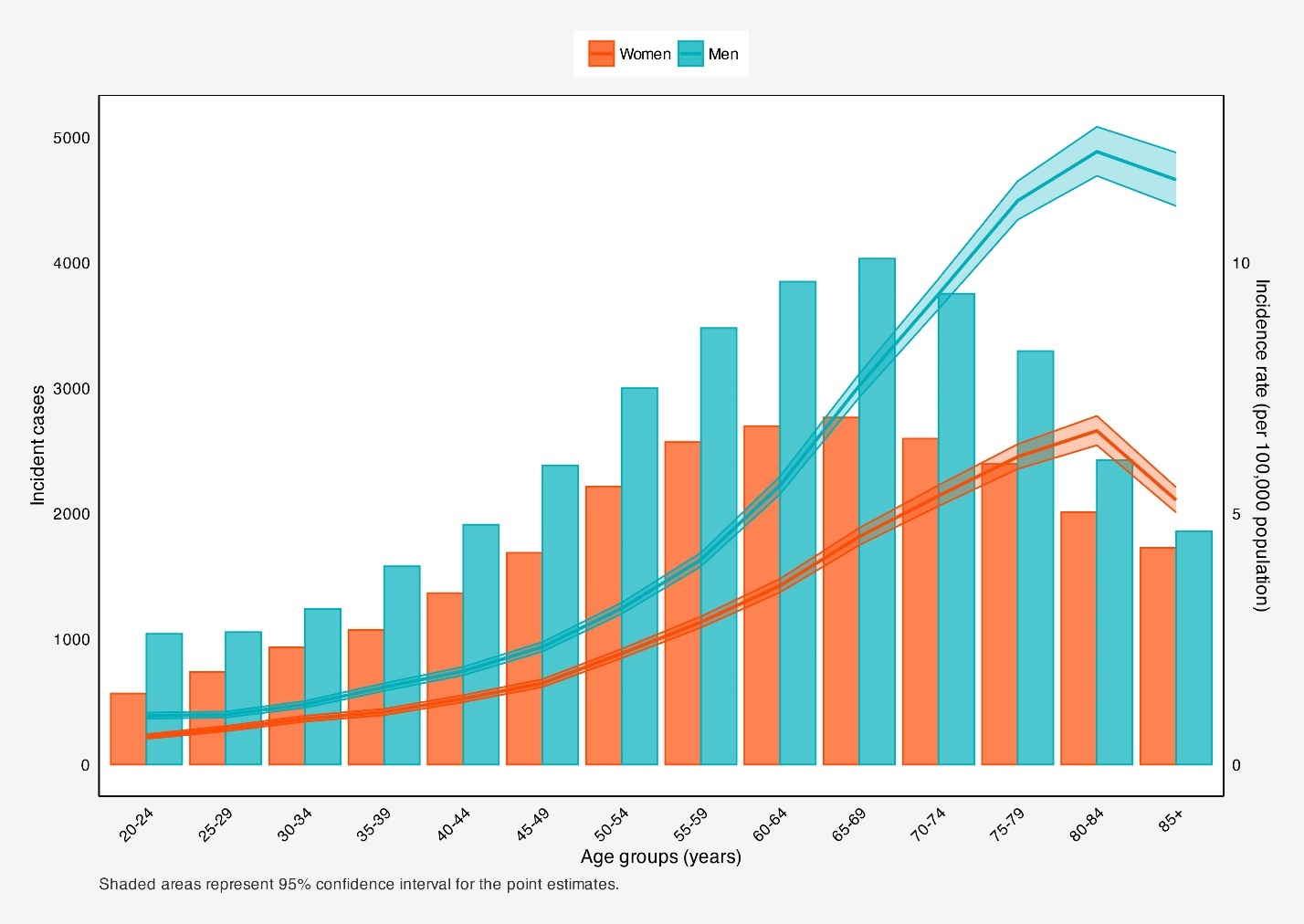


**Figure S21.** Incident cases and incidence rate of T-cell non-Hodgkin lymphoma in the United States among males and females in each age group.


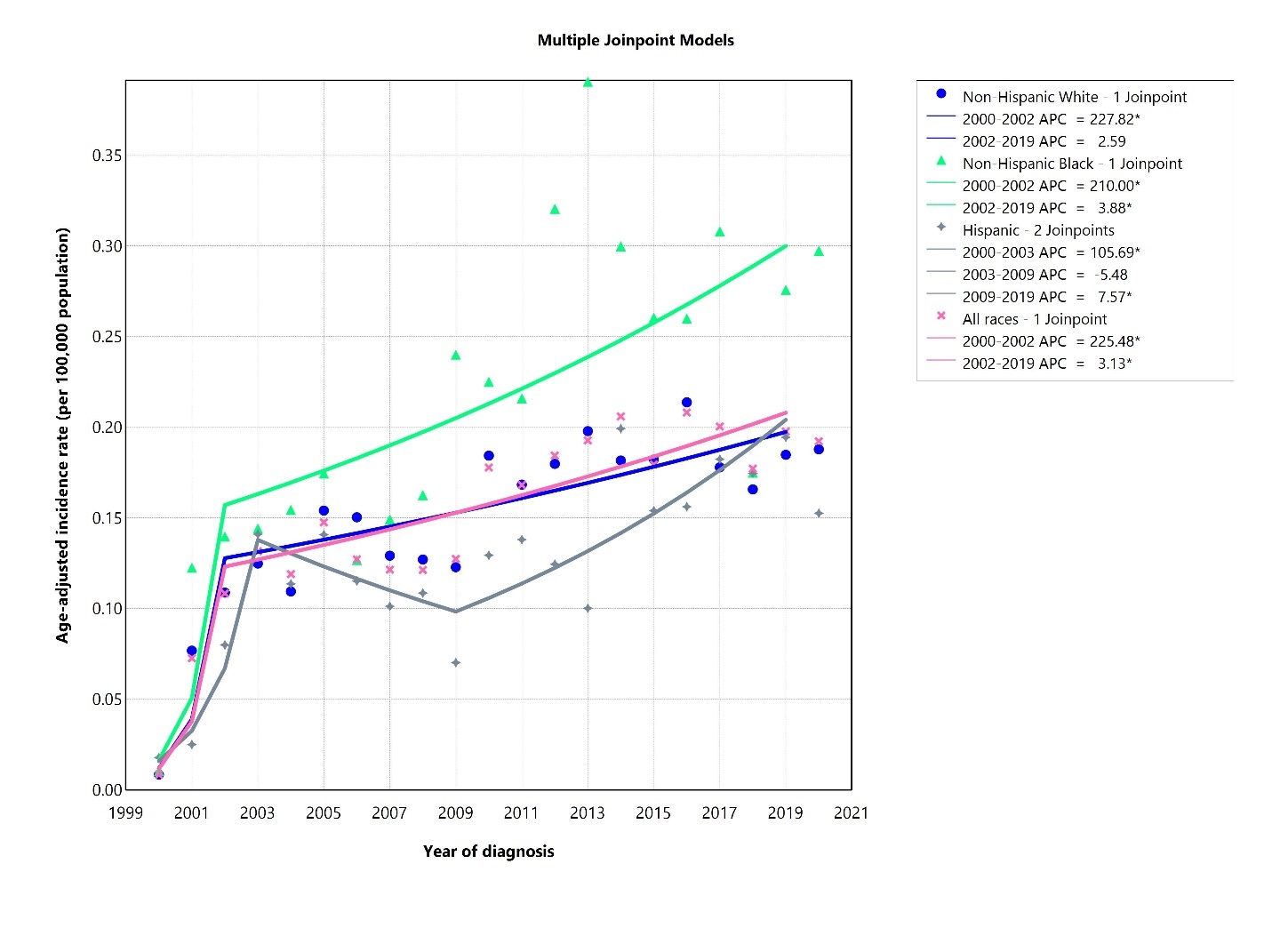


**Figure S22.** The age-adjusted incidence rate of precursor T-cell non-Hodgkin lymphoma in adults over 2000-2019 and 2020 in the United States, by race. APC: annual percent change. * Represents a p-value less than 0.05.


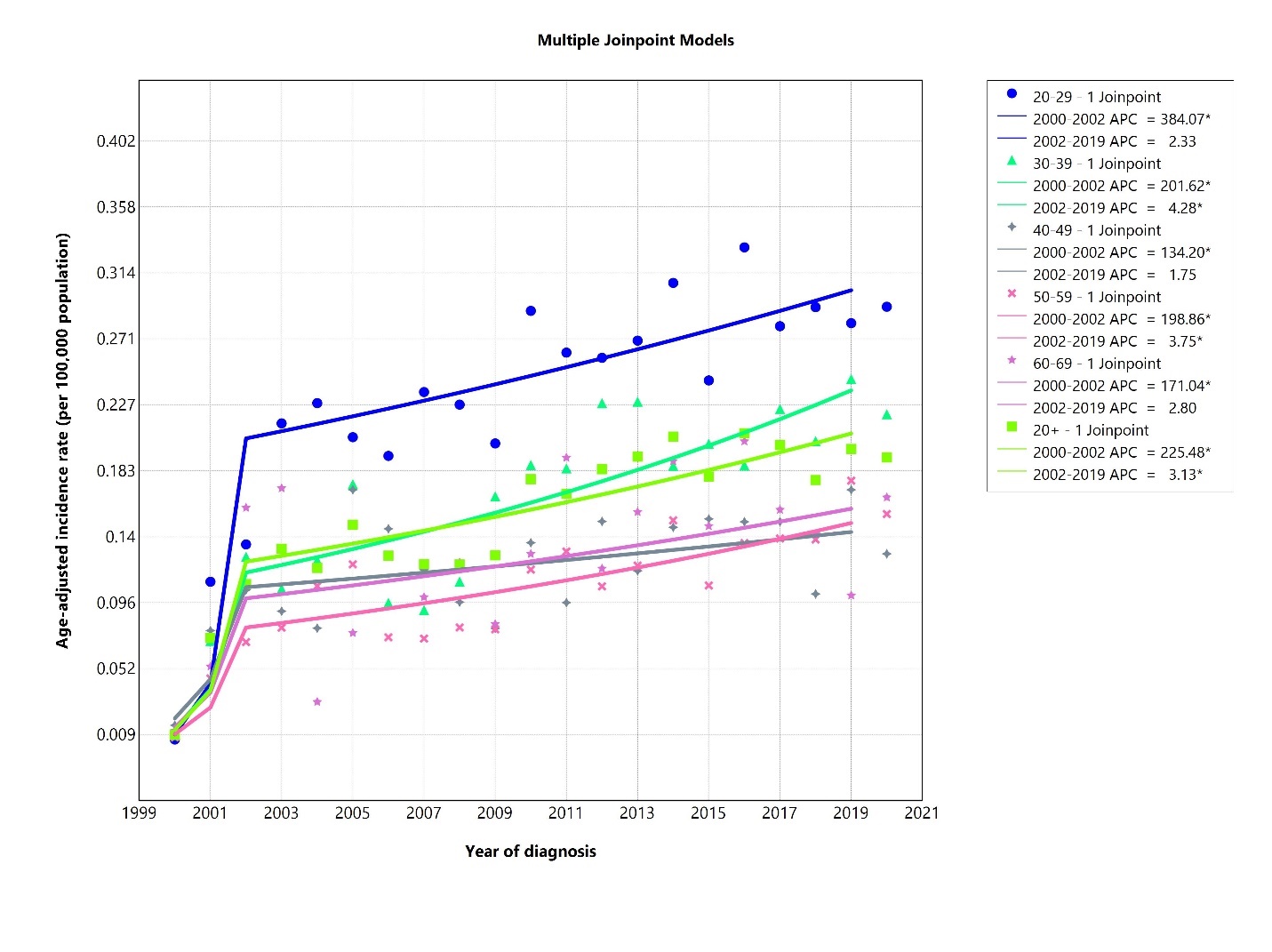


**Figure S23.** The age-adjusted incidence rate of precursor T-cell non-Hodgkin lymphoma in adults over 2000-2019 and 2020 in the United States, by age. APC: annual percent change. * Represents a p-value less than 0.05.


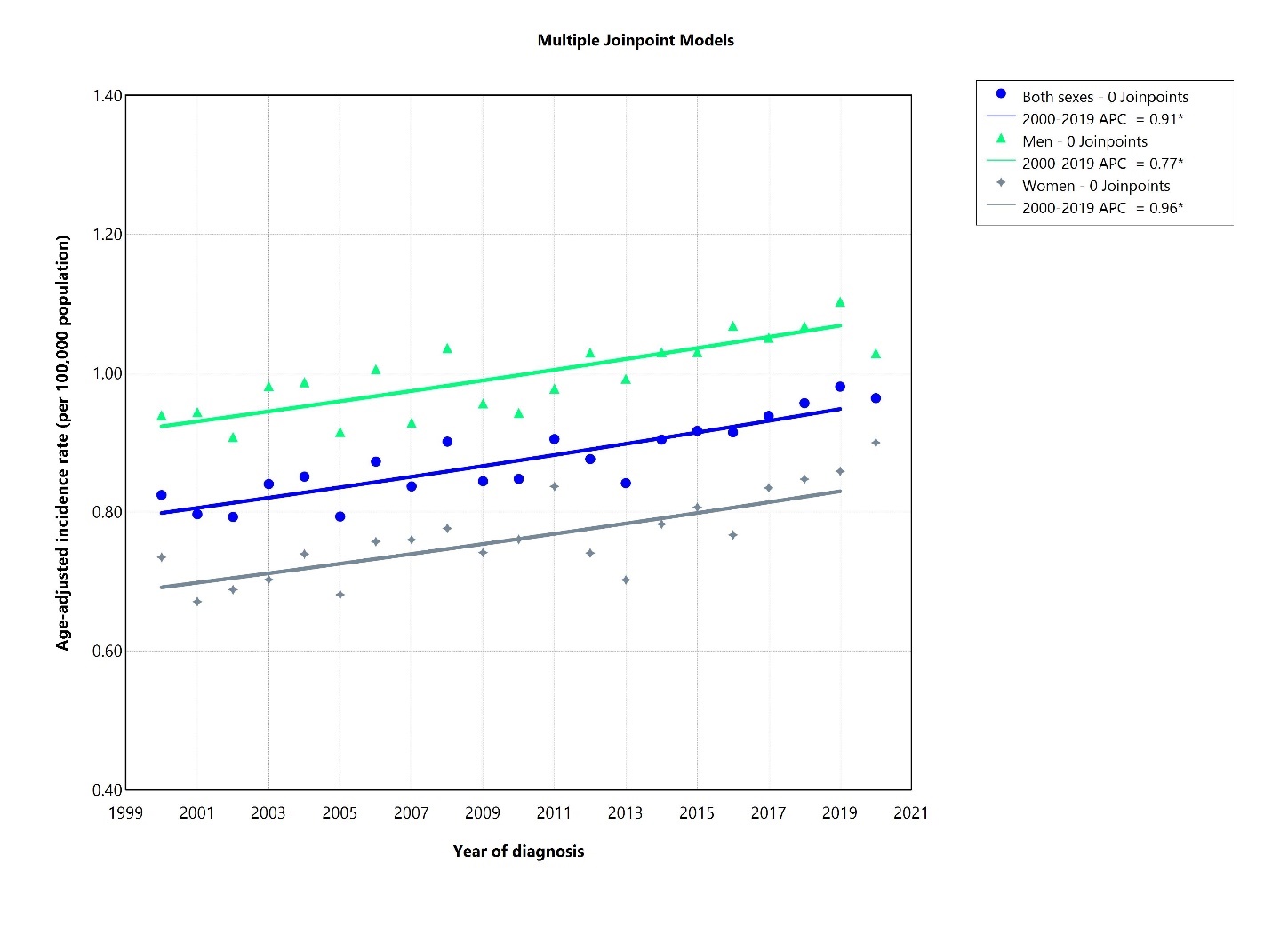


**Figure S24.** The age-adjusted incidence rate of precursor T-cell non-Hodgkin lymphoma in adults over 2000-2019 and 2020 in the United States, by sex. APC: annual percent change. * Represents a p-value less than 0.05.


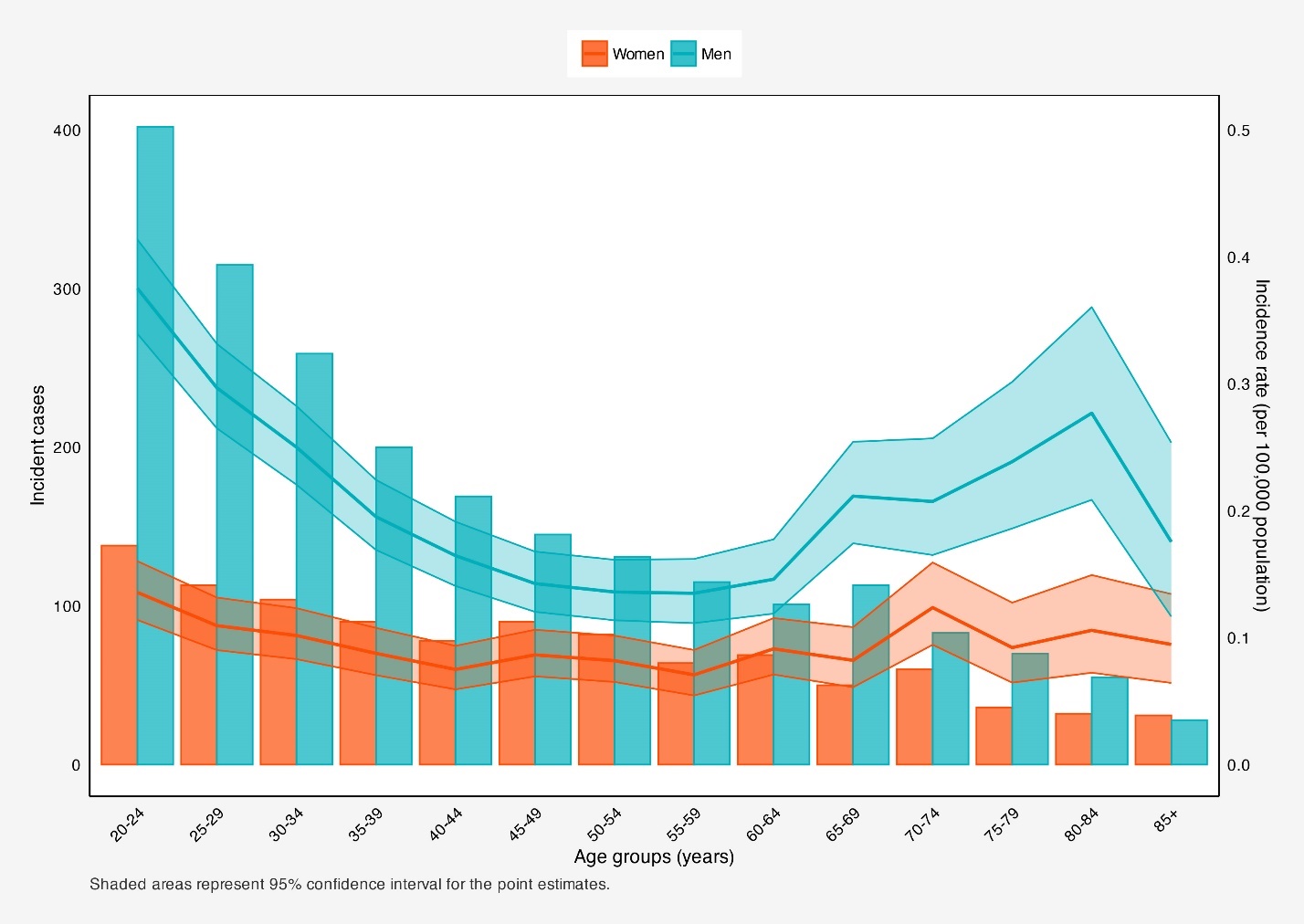


**Figure S25.**Incident cases and incidence rate of precursor T-cell non-Hodgkin lymphoma in the United States among males and females in each age group.


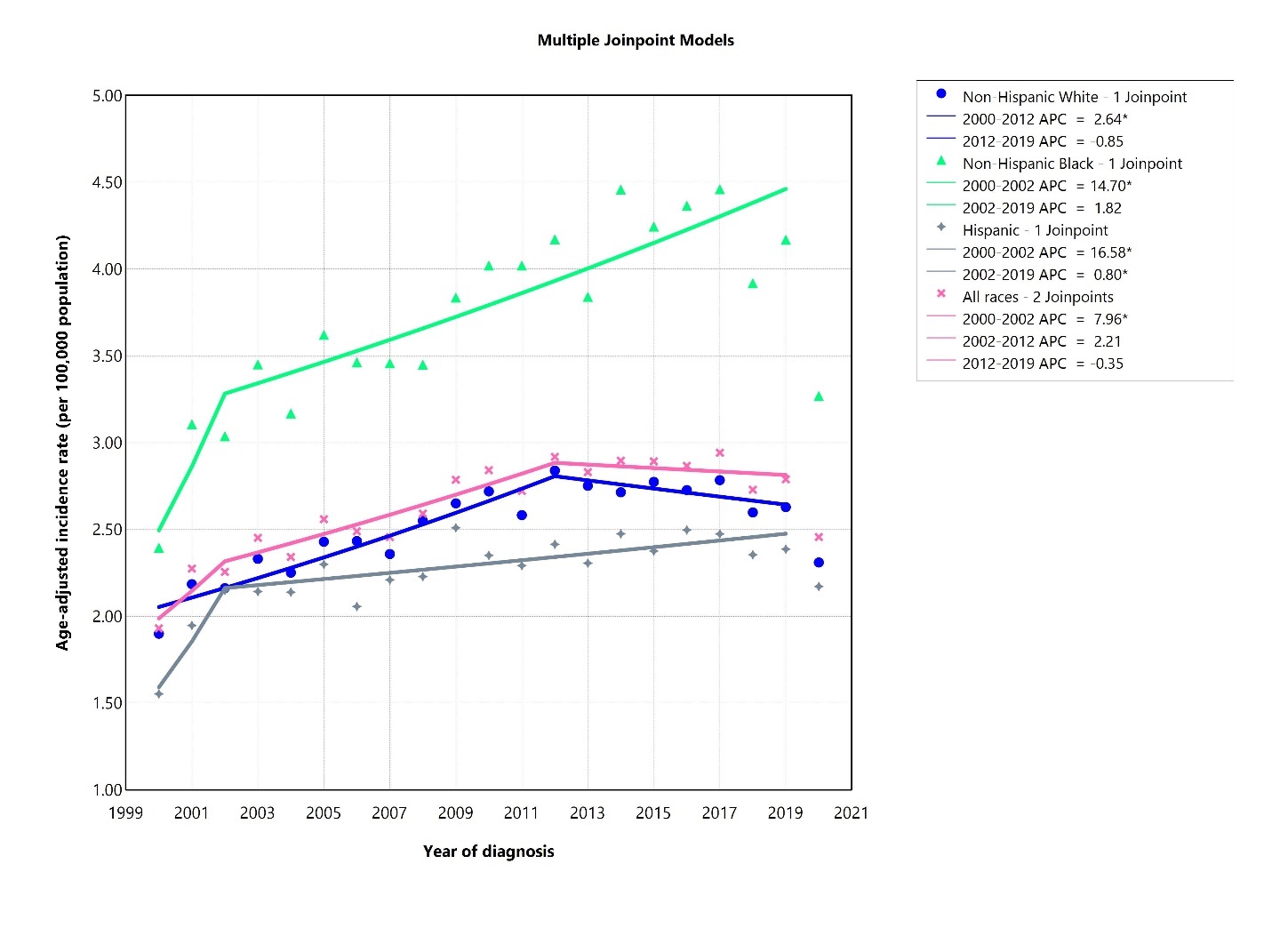


**Figure S26.** The age-adjusted incidence rate of mature T-cell non-Hodgkin lymphoma in adults over 2000-2019 and 2020 in the United States, by race. APC: annual percent change. * Represents a p-value less than 0.05.


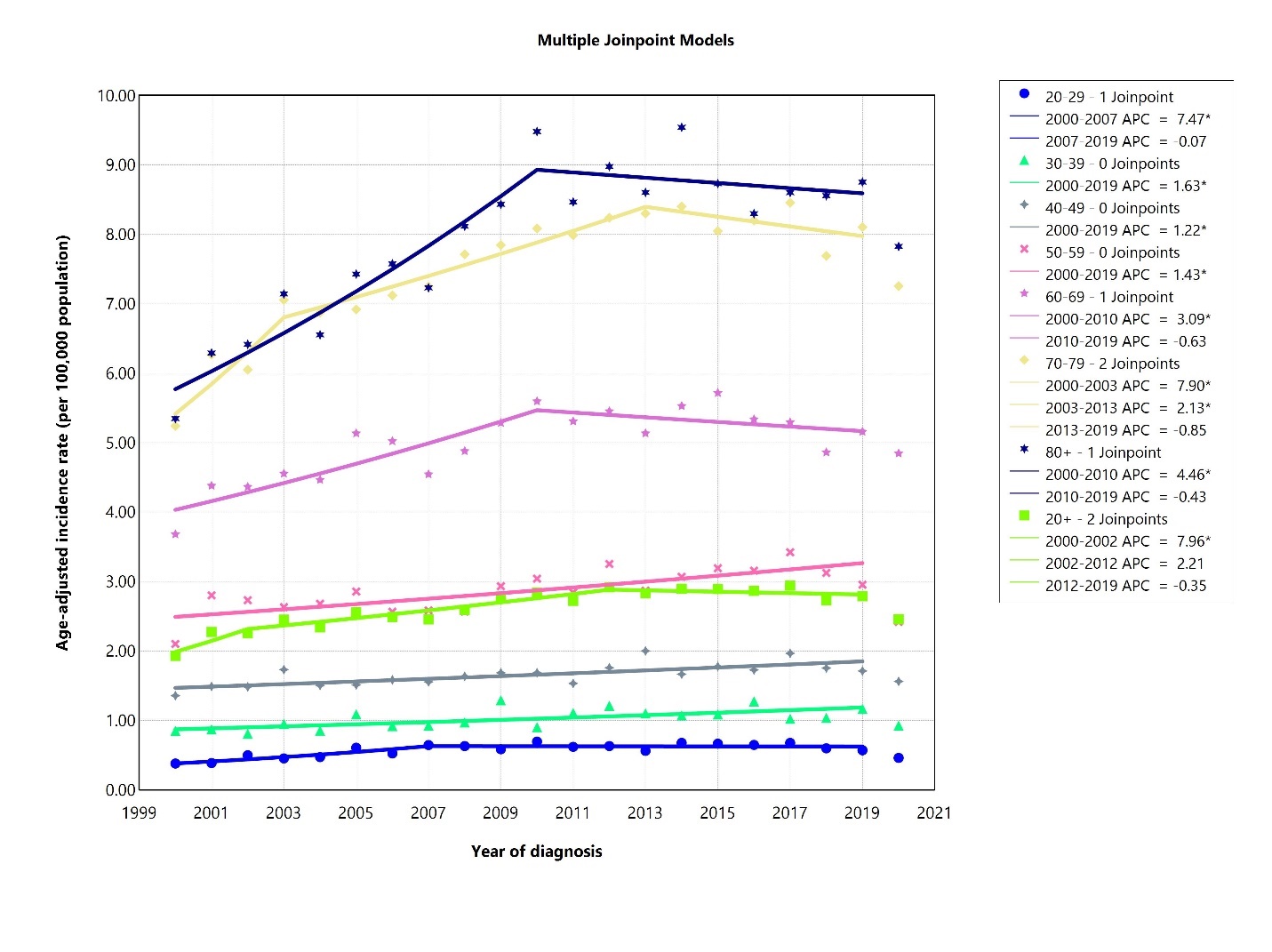


**Figure S27.** The age-adjusted incidence rate of mature T-cell non-Hodgkin lymphoma in adults over 2000-2019 and 2020 in the United States, by age. APC: annual percent change. * Represents a p-value less than 0.05.


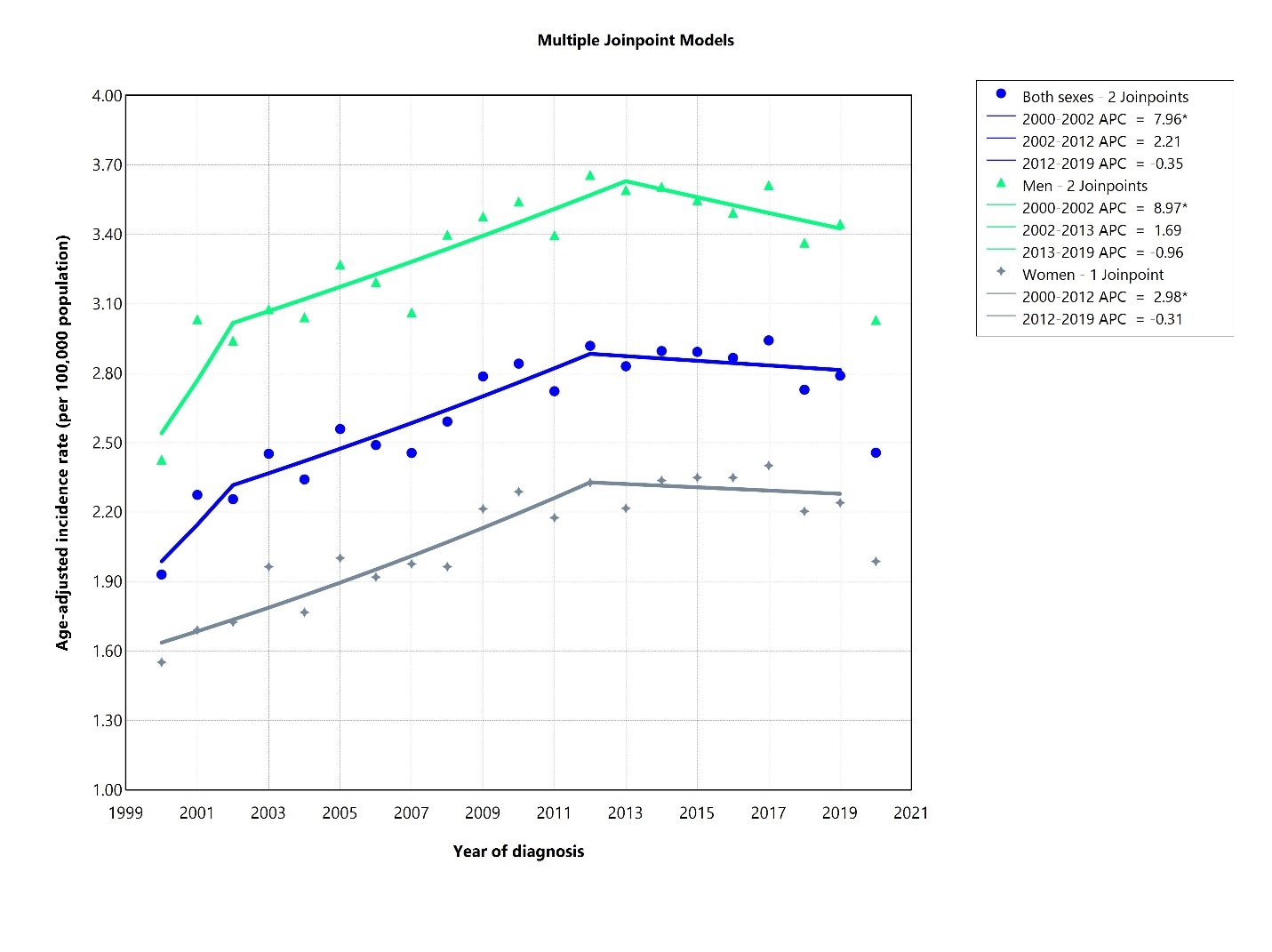


**Figure S28.** The age-adjusted incidence rate of mature T-cell non-Hodgkin lymphoma in adults over 2000-2019 and 2020 in the United States, by sex. APC: annual percent change. * Represents a p-value less than 0.05.


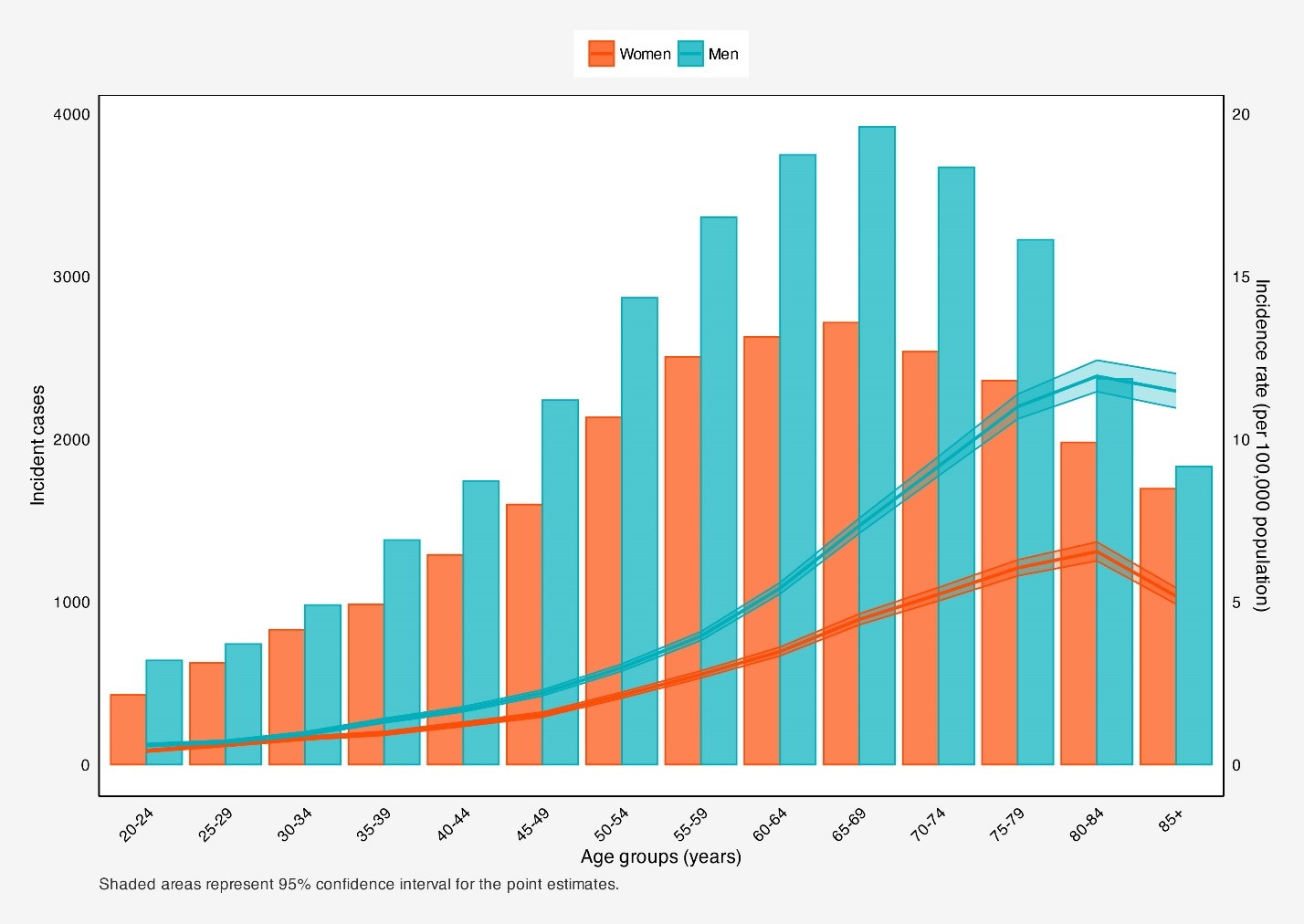


**Figure S29.** Incident cases and incidence rate of mature T-cell non-Hodgkin lymphoma in the United States among males and females in each age group.


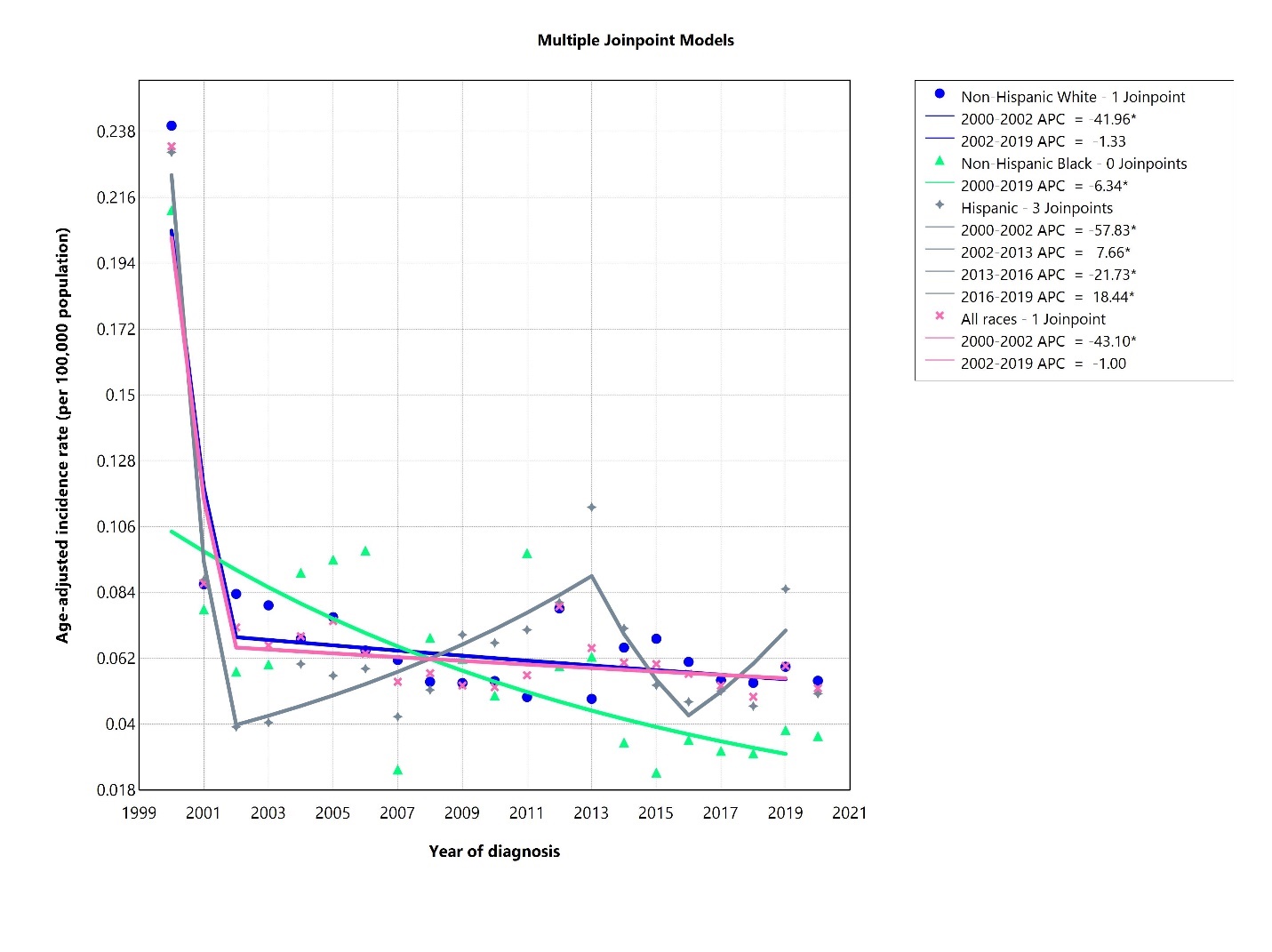


**Figure S30.** The age-adjusted incidence rate of non-Hodgkin lymphoma with unknown lineage in adults over 2000-2019 and 2020 in the United States, by race. APC: annual percent change. * Represents a p-value less than 0.05.


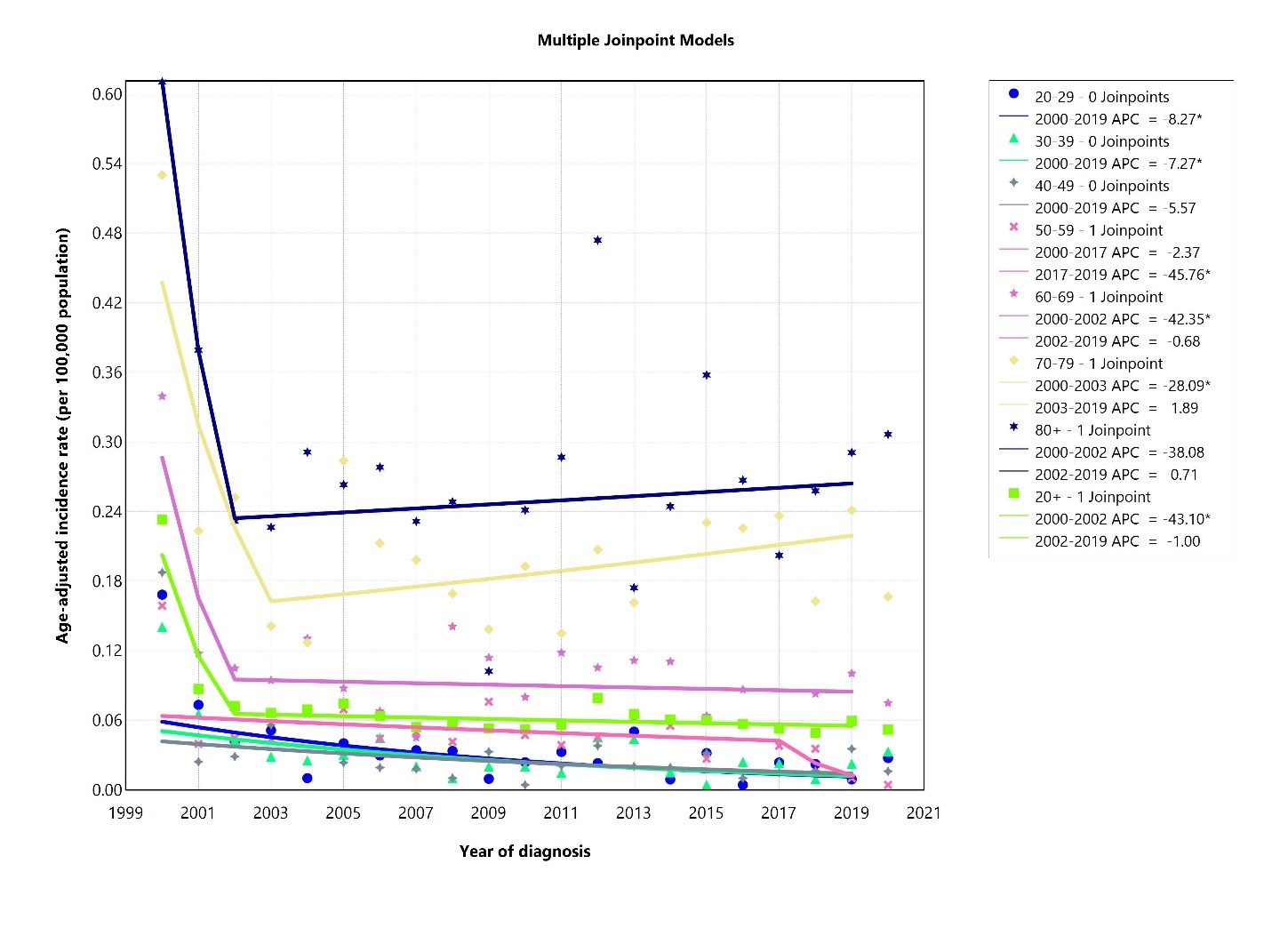


**Figure S31.** The age-adjusted incidence rate of non-Hodgkin lymphoma with unknown lineage in adults over 2000-2019 and 2020 in the United States, by age. APC: annual percent change. * Represents a p-value less than 0.05.


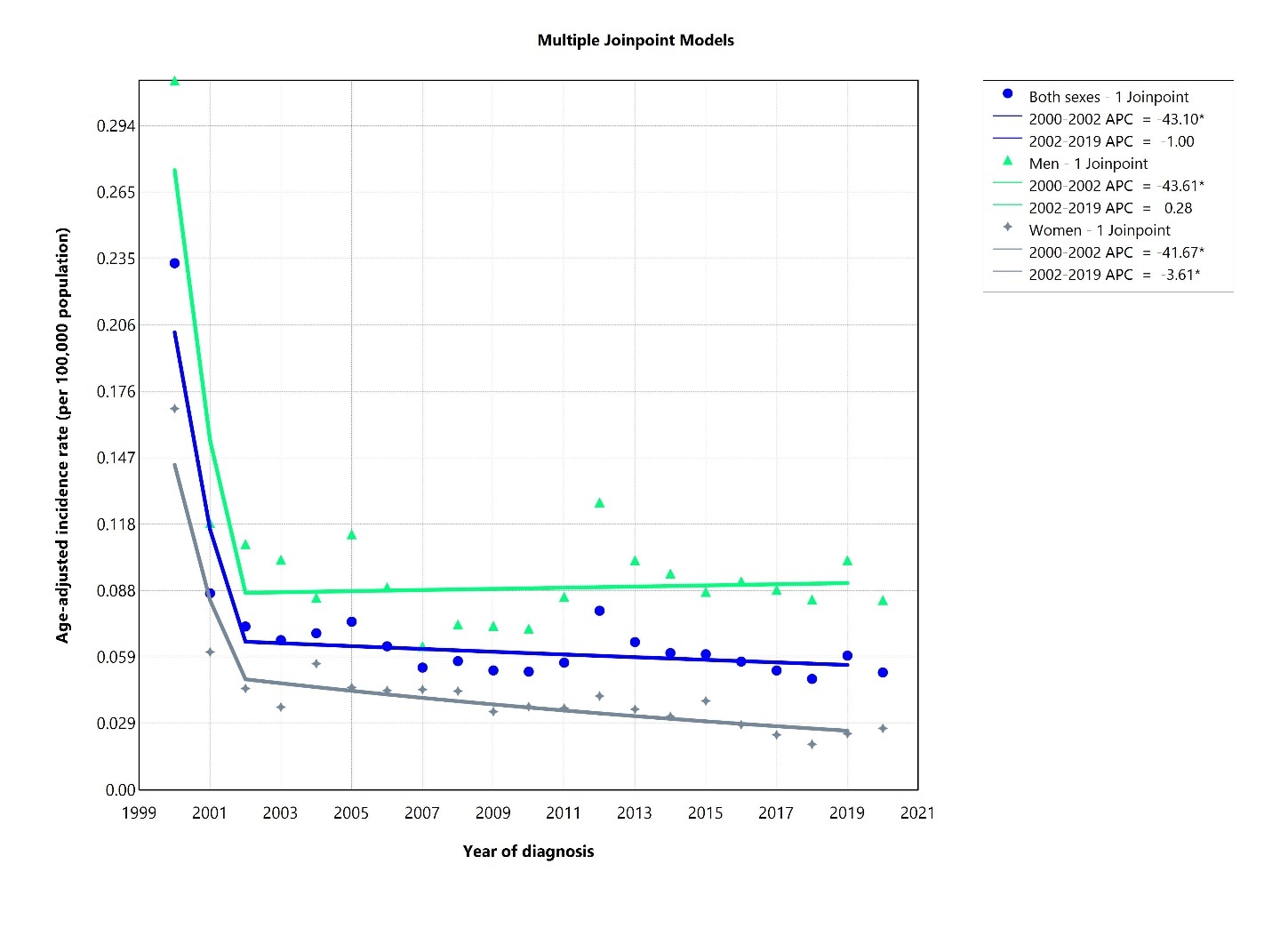


**Figure S32.** The age-adjusted incidence rate of non-Hodgkin lymphoma with unknown lineage in adults over 2000-2019 and 2020 in the United States, by sex. APC: annual percent change. * Represents a p-value less than 0.05.


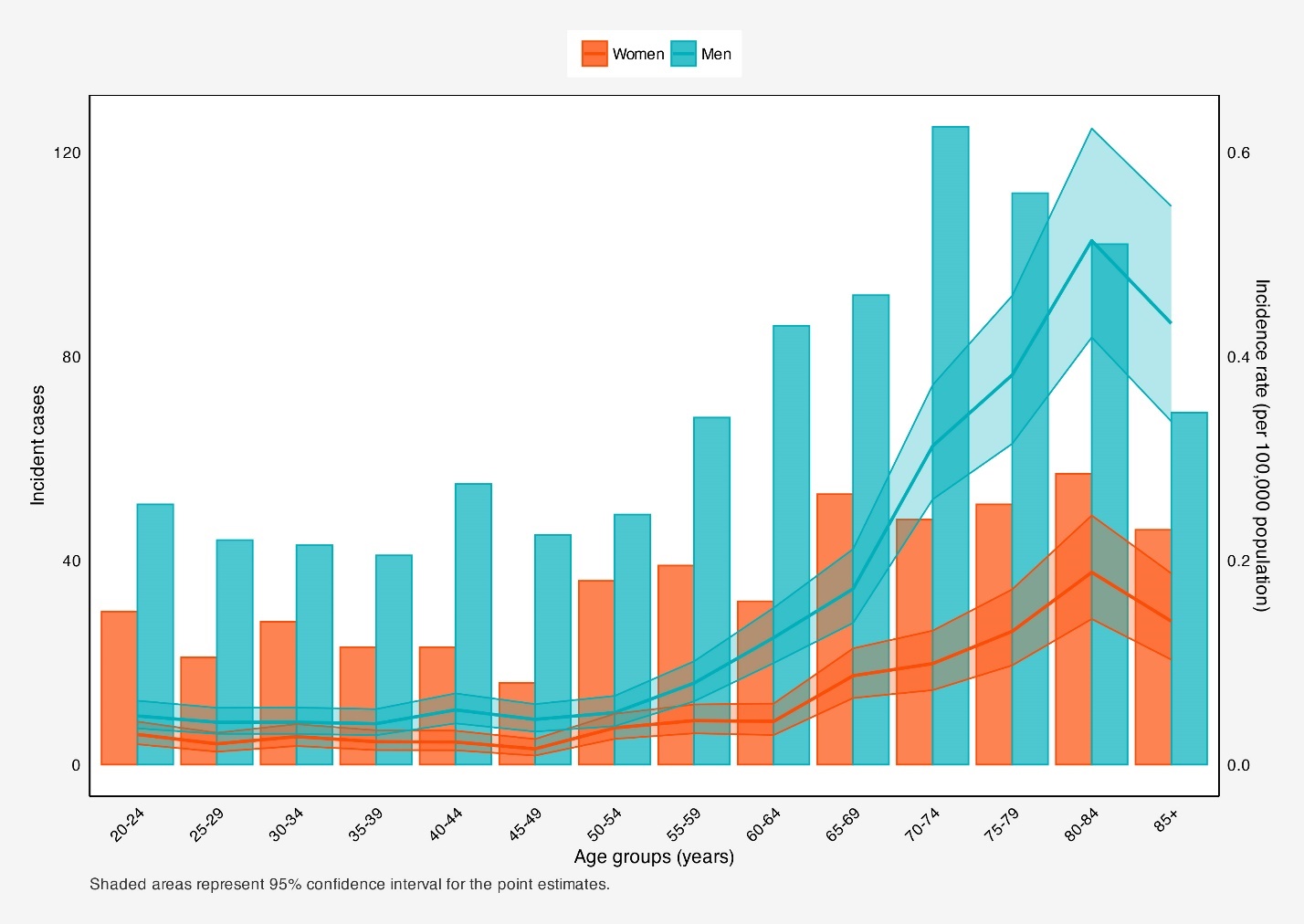


**Figure S33.** Incident cases and incidence rate of non-Hodgkin lymphoma with unknown lineage in the United States among males and females in each age group.
